# Supplementary material for: An open-source FACS automation system for high-throughput cell biology
Source: PLoS One. 2024 Mar 21;19(3):e0299402. doi: 10.1371/journal.pone.0299402 (PMC10956866; doi:10.1371/journal.pone.0299402)
Supplement: S2 File — (PDF) [file pone.0299402.s006.pdf]

Hardware BOM - FACS Automation

Part number	Quantity	Vendor	Name	Description
**1-0015-V1.1 FACS Assembly**			**FACS Automation project**	**Integration of automated software and hardware to the Sony Sorter SH800 instrument. A 3-axis stage with gripper is used to move sample tubes from a cooling chamber housing to the Sony Sorter instrument.**
SH800S	1	Sony	Sony SH800S Cell Sorter	Sony SH800 FACS instrument rendering; not the official CAD file.
LE-U3M004 SYX	1	Sony	Sony LE-U3M004 SYX SH800 Sample Holder (5mL)	Tube adapter for the Sony SH800. It magnetically seats into the instrument, and holds the sample tube. Not official CAD file.
3-0630	1	3D Printed (SLA)	3-0630 Sony Adapter Funnel	Funnel used for FACS automation so that the grippers can deliver the tube into the Sony 0.5 mL tube adapter.
352235	18	Falcon	Falcon 352235 Culture Test Tube	12x75 mm sample tube used with the Sony Sorter FACS instrument
3-0613	1	Laser Cut	3-0613 Sony Sorter Acrylic Fluidics Maintenance Door	Acrylic door to replace the factory door of the Sony Sorter for the FACS Automation project. This is to allow for the clearance within the biosafety cabinet of the door to access the fluidics inside of the Sony Sorter.
3-0757	1	Outside Machine Shop	3-0757 Sony Vacuum Plate Replacement	A replacement plate for the HEPA exhaust vent plate to add an L-shaped tube fitting. This allows for the Sony Sorter to be moved inside of the BSC so that the FACS automation hardware fits inside of the BSC.
93475A230	12	McMaster-Carr	Chamfered plain washer normal M4	Chamfered plain washer normal M4 Stainless Steel
91292A118	6	McMaster-Carr	Hex socket head cap screw M4x0.70 x 16	Hex socket head cap screw M4x0.70 x 16 Stainless Steel
91828A231	12	McMaster-Carr	Hex nut grade A & B M4x0.7	Hex nut grade A & B M4x0.7 Stainless Steel
LSQ450x E01T4A ENG2710	1	Zaber	Zaber LSQ450x E01T4A ENG2710 Linear Stage	Zaber Technologies 450 mm linear translation stage
LSQ075B-T4-ENG2690	1	Zaber	Zaber LSQ075B-T4-ENG2690	Zaber Technologies 75 mm linear stage
LSQ150B-T4A	1	Zaber	Zaber LSQ150B-T4A Customer Facing	Zaber Technologies 150 mm linear stage
X-GLP-E03	1	Zaber	Zaber X-GLP-E03	Zaber Technologies grippers. with 20 mm travel
3-0297-TBO	1	Outside Machine Shop	3-0297-TBO Top Claw Blunted Off-axis	Claw affixed to the Zaber gripper to grab and hold FACS sample tubes for the FACS automation project
3-0297-BBO	1	Outside Machine Shop	3-0297-BBO Bottom Claw Blunted Off-axis	Claw affixed to the Zaber gripper to grab and hold FACS sample tubes for the FACS automation project
3-0312-V1.1	1	Outside Machine Shop	3-0312-V1.1 Mounting plate	The plate for mounting all of the hardware for the FACS Automation project. Version 1.1
3-0310-V1.1	2	Outside Machine Shop	3-0310-V1.1 Primary clamp finger	Primary clamp finger to attach and align the automation rig to the Sony sorter foot. V1.1
3-0311-V1.1	2	Outside Machine Shop	3-0311-V1.1 Secondary clamp	Secondary clamp, version 1.1
92196A168	4	McMaster-Carr	Socket head cap screw #6-32 x 1.75	Socket head cap screw #6-32 x 1.75 Stainless Steel
3-0313-V1.1	1	Outside Machine Shop	3-0313-V1.1 Clamp mounting block	Block to align and mate between the Sony clamps and the automation plate, version 1.1
3-0574	1	3D printed (FFF)	3-0574 FACS tube holder adapter	Adapter part between mounting plate and tube holder to align the tube holder at the appropriate location for FACS automation
92196A148	4	McMaster-Carr	Socket head cap screw #6-32 x 0.5	Socket head cap screw #6-32 x 0.5 Stainless Steel
2471N2	2	McMaster-Carr	McMaster 2471N2 Pull Handle	McMaster-Carr pull handle with mounting hole center-to-center width of 3 15/16", fits M5 screws, and load capacity 220 lbs.
92196A272	4	McMaster-Carr	Socket head cap screw #10-32 x 0.75	Socket head cap screw #10-32 x 0.75 Stainless Steel
680	1	Vortec	Vortec 680 Mini Cold Air Gun and Filter	Vortec Mini Air Gun and Filter
3-0541	1	3D printed (FFF)	3-0541 Air Gun Adapter	3D printed part to interface between the Vortec mini air gun exhaust and 1/4" quick release fittings
3-0314	1	3D printed (FFF)	3-0314 Air Gun Adapter Sleeve	The sleeve mates between the air gun and the air gun adapter to make a more rigid connection to them.
94180A331	7	McMaster-Carr	McMaster 94180A331 M3 threaded insert	M3 threaded insert
91217A070	4	McMaster-Carr	McMaster 91217A070 M3 Set Screw	M3x0.5x8 mm set screw
CL3	2	Thorlabs	Thorlabs CL3 Compact Variable Height Clamp	Thorlabs CL3 Compact Variable Height Clamp for Optical Tables and 1/4"-20 screws
92196A542	2	McMaster-Carr	Socket head cap screw 1/4-20 x 1	Socket head cap screw 1/4-20 x 1 Stainless Steel
92196A537	10	McMaster-Carr	Socket head cap screw 1/4-20 x 0.5	Socket head cap screw 1/4-20 x 0.5 Stainless Steel
3-0628	1	Outside Machine Shop	3-0628 L-bracket clamp to plate	L-bracket to align and secure the 1/4" mounting plate to the bracket clamped to the Sony Sorter for the FACS Automation project.
92196A146	2	McMaster-Carr	Socket head cap screw #6-32 x 0.375	Socket head cap screw #6-32 x 0.375 Stainless Steel
3-0627	1	3D printed (FFF)	3-0627 Air Filter Holder Base	Base of housing for the air filter suppled with the Vortec cold air gun to keep the filter upright, and provide mounting locations.
AR30-N03BG-Z-B	1	SMC	SMC AR30-N03BG-Z-B Pressure Regulator	Air regulator
97395A451	4	McMaster-Carr	McMaster 97395A451 0p125in dia. x 0p75in long SS Dowel Pin	Dowel pin, 1/8" dia, 3/4" long, 316 stainless steel
2313N793	4	McMaster-Carr	McMaster 2313N793 CORNER MACHINE BRACKET	L-bracket using 1/4" screws. Used in the FACS automation project to screw the electronics box to the mounting plate off of the side of the plate.
92196A535	6	McMaster-Carr	Socket head cap screw 1/4-20 x 0.375	Socket head cap screw 1/4-20 x 0.375 Stainless Steel
92196A534	2	McMaster-Carr	Socket head cap screw 1/4-20 x 0.3125	Socket head cap screw 1/4-20 x 0.3125 Stainless Steel
3-0674	1	3D printed (FFF)	3-0674 Solenoid Adapter Plate	Plate to mount the solenoid valve to a bracket that affixes to the mounting plate. The hole spacing for the solenoid is unique such that a standard L-bracket will not suffice without the adapter.
8262H022	1	Asco Red Hat	Asco Red Hat 8262H022 Solenoid Valve	Solenoid valve, normally close, 12 V DC, Cv 0.35.
91292A126	2	McMaster-Carr	Hex socket head cap screw M5x0.80 x 16	Hex socket head cap screw M5x0.80 x 16 Stainless Steel
93365A160	10	McMaster-Carr	McMaster 93365A160 Heat-Set Inserts For Plastics	1/4"x20 threaded insert
3-1095	1	3D printed (FFF)	3-1095 FACS Electronics Box Case	Case to hold the Arduino Mega and FACS PCB
3-1096	1	3D printed (FFF)	3-1096 FACS Electronics Box Lid	Lid to case to hold the Arduino Mega and FACS PCB
99461A825	24	McMaster-Carr	McMaster 99461A825 - SS thread-forming screw, round head, PH drive, #3 - 3/8" long	Stainless steel thread-forming screw for plastics, round head, Phillips drive, #3 - 3/8" long. Drill diameter: 0.081"
93655A220	3	McMaster-Carr	McMaster-Carr 93655A220 Male-Female Threaded Hex Standoff M3	18-8 Stainless Steel, 4.500 mm Hex, 11 mm Long, M3 x 0.50 mm Thread
91292A110	3	McMaster-Carr	Hex socket head cap screw M3x0.50 x 5	Hex socket head cap screw M3x0.50 x 5 Stainless Steel
A08000	1	Digikey	Arduino Mega	The 8-bit board with 54 digital pins, 16 analog inputs, and 4 serial ports
3-1209	1	Laser Cut	3-1209 Electronics Box Adapter Strip	Adapter strip for 1/4"-20 screws to fasten the electronics box to the L-brackets on the FACS automation plate due to the limited length of the ribbon cables
5-2004	1	OSH Park	5-0004 FACS PCB v2	Main PCB: H-bridge motor drivers, a transistor for solenoid valve, and circuitry for thermistors
3-0722	1	3D printed (FFF)	3-0722 Air Filter Holder Top	Top of housing for the air filter suppled with the Vortec cold air gun to keep the filter upright, and provide mounting locations.
99461A150	4	McMaster-Carr	McMaster 99461A150 - SS thread-forming screw, round head, PH drive, #4 - 3/4" long	Stainless steel thread-forming screw for plastics, round head, Phillips drive, #4 - 3/4" long. Drill diameter: 0.086"
3-1116	2	Laser Cut	3-1116 Tube housing attachment bracket	Bracket to attach the tube housing to the the tube holder cantilever that attaches to the automation alignment plate
3-0281	2	3D printed (FFF)	3-0281 Motor plate	FACS automation plate interface for DC motors to seat within the housing
3-0282-18T	1	3D printed (FFF)	3-0282-18T Housing	FACS automation housing for the sample tubes, DC motors, coupling parts and cooling. This configuration holds 18 tubes.
3-0283	2	3D printed (FFF)	3-0283 Test tube guide	FACS automation part to support test tubes within the tube housing
3-0284	2	Laser Cut	3-0284 Rubber insert	FACS automation rubber gasket for tube holder to reduce the heat transfer between the cooled tube holder, and ambient conditions
3-0285	2	3D printed (FFF)	3-0285 Top plate	FACS automation top plate to close the tube housing and clamp the test tube guide and rubber insert in place.
DB25-SF-M1	2	Digikey	Digikey DB25-SF-M1	DB25 connector from Adam Tech
3-0713	1	Laser Cut	3-0713 Rear Gasket	A gasket between the rear access plate and the tube housing to limit any cold air from escaping.
3-0712	1	3D printed (FFF)	3-0712 Rear Access Plate	A plate to provide access to the back of the tube holder for the FACS automation project
3-0280	18	Outside Machine Shop	3-0280 Test Tube Socket	Socket interface for the DC motor to rotate test tubes for the FACS automation instrument.
3038	18	Pololu	Pololu Micro Metal Gearmotor HPCB	High-power carbon brush motor
92000A116	18	McMaster-Carr	PH Pan head screw M3x0.50 x 6 mm	PH Pan head screw M3x0.50 x 6 Stainless Steel
98689A112	18	McMaster-Carr	Chamfered plain washer normal M3	Chamfered plain washer normal M3 Stainless Steel
97259A101	18	McMaster-Carr	McMaster 97259A101 M3 Low-Strength Steel Thin Square Nut	M3 Square Nut Stainless Steel
91292A260	36	McMaster-Carr	Hex socket head cap screw M1.6x0.35 x 3 mm	Hex socket head cap screw M1.6x0.35 x 3 Stainless Steel
99461A815	20	McMaster-Carr	McMaster 99461A815 - SS thread-forming screw, round head, PH drive, #3 - 1/4" long	Stainless steel thread-forming screw for plastics, round head, Phillips drive, #3 - 1/4" long. Drill diameter: 0.081"
92196A108	6	McMaster-Carr	Pan head machine screw #4-40 x 0.375	Pan head machine screw #4-40 x 0.375 Stainless Steel
99461A130	14	McMaster-Carr	McMaster 99461A130 - SS thread-forming screw, round head, PH drive, #4 - 1/2" long	Stainless steel thread-forming screw for plastics, round head, Phillips drive, #4 - 1/2" long. Drill diameter: 0.086"
5-2007	2	OSH Park	5-0007 FACS Photointerruptor Breakout Board	The photointerruptor board used in the FACS Automation tube housing to stop the motors at a specific location for ease of tube grabbing.
3-0614	8	Laser Cut	3-0614 Motor Encoder PCB Spacer	Spacer to ensure the optical reflectors are within the distance of detection of the tube sockets to be used as motor encoders for the FACS Automation project.
92196A140	4	McMaster-Carr	Socket head cap screw #2-56 x 1	Socket head cap screw #2-56 x 1 Stainless Steel
91841A003	8	McMaster-Carr	Hex machine screw nut #2-56	Hex machine screw nut #2-56 Stainless Steel

92196A083	4	McMaster-Carr	Socket head cap screw #2-56 x 0.625	Socket head cap screw #2-56 x 0.625 Stainless Steel
215ME-ND	1	Digikey	Digikey 215ME-ND	15 Position D-Sub Plug, Male Pins Connector
91841A005	6	McMaster-Carr	Hex machine screw nut #4-40	Hex machine screw nut #4-40 Stainless Steel
92196A147	2	McMaster-Carr	Socket head cap screw #6-32 x 0.4375	Socket head cap screw #6-32 x 0.4375 Stainless Steel
92196A150	4	McMaster-Carr	Socket head cap screw #6-32 x 0.625	Socket head cap screw #6-32 x 0.625 Stainless Steel
3-1168	1	Laser Cut	3-1168 Tube housing front plate	A front cross plate to keep the tube housing mounted to the tube holder adapter
92196A533	4	McMaster-Carr	Socket head cap screw 1/4-20 x 0.25	Socket head cap screw 1/4-20 x 0.25 Stainless Steel
X-MCC3	1	Zaber	Zaber X-MCC3 Universal Motor Controller	Motor controller for Zaber stages.
3-0694	1	3D printed (FFF)	3-0694 Motor controller plate	Plate to mount the Zaber motor controller and hang from the sidewall of the FACS automation BSC.
3013T45	1	McMaster-Carr	McMaster 3013T45 Steel Eyebolt Without Shoulder	Steel eyebolt for lifting up to 500 lbs.
5648K69	12 ft	McMaster-Carr	Firm Polyurethane Tubing for Air and Water, 5/32" ID, 1/4" OD	Tubing for compressed air supply to cold air gun
5779K669	1	McMaster-Carr	Push-to-Connect Tube Fitting for Air, Inline Tee Reducer, for 1/4" x 3/8" Tube OD	Reducing fitting for compressed air tubing
5779K34	2	McMaster-Carr	Push-to-Connect Tube Fitting for Air, Tee Connector, for 1/4" Tube OD	T connector fitting for compressed air tubing
5779K44	1	McMaster-Carr	Push-to-Connect Tube Fitting for Air, Wye Connector, for 1/4" Tube OD	W connector fitting for compressed air tubing
5779K24	4	McMaster-Carr	Push-to-Connect Tube Fitting for Air, 90 Degree Elbow Connector, for 1/4" Tube OD	90 deg connector fitting for compressed air tubing
45975K45	1	McMaster-Carr	Gray PVC On/Off Valve, Straight, Push-to-Connect for 1/4" Tube OD, Full Port	On-Off valve for compressed air tubing
5779K109	7	McMaster-Carr	Push-to-Connect Tube Fitting for Air, Straight Adapter, for 1/4" Tube OD x 1/4 NPT Male	NPT to tubing connector for compressed air tubing
IPAPT03838	4 ft	Armacell	AP Armaflex Insulation, ⅜” wall thickness, 1/4" Copper	Insulation for cold air tubing
8908K38	1 pack	McMaster-Carr	304 Stainless Steel Wire, 0.037” dia x 12”	2 wires needed from pack
5777T11	1	McMaster-Carr	Silicone rubber sheet with crisscross texture, 50A durometer, 1/32” thick, 12" x 12"	Silicone sheet for tube housing sample tube gasket
3788T22	1	McMaster-Carr	Silicone rubber sheet with smooth texture, 50A durometer, 1 mm thick, 12" x 12"	Silicone sheet for gaskets
8589K62	1	McMaster-Carr	Clear Scratch- and UV-Resistant Acrylic Sheet, 12" x 24" x 3/16"	Acrylic sheet stock for laser-cut acrylic door
RS-F2-DUCL-02	1 L	Formlabs	Durable Resin	3D printing resin
M-KVD-VTPW	0.75 kg	MatterHackers	Ultimaker PVA Filament - 2.85mm	3D printing material
HTP32830-BLK	3 kg spool	Protopasta	Black Opaque HTPLA Filament	3D printing material
7468A62	8 fl oz	McMaster-Carr	Pliobond 25	Rubber cement for claws and tube sockets
402026-WHT-12x2	2	Cable Matters	Outlet Surge Protector Power Strip with USB, 12 ft long Extension Cord with Low Profile Plug	Surge protector for computer and automation hardware
HB-UM43	1	Sabrent	4 Port uSB 3.0 Hub with Individual LED Power Switches	USB hub for automation hardware
90086	1	Velcro	Velcro Sticky Back Tape, 5’ x ¾”	Velcro strips for cable management / other device management
70215K101	1	McMaster-Carr	170-Piece UV-Resistant Cable Tie Assortment	ZIp-ties for cable management
6334K41	1	McMaster-Carr	Heat Shrink Tubing Kit	12-8 AWG (3/8" Expanded), 2 pieces for the aluminum claws
458742928317	1	Reynolds Wrap	Aluminum Foil 200 sq ft.	Foil for tube sockets reflective strips
