# Supplementary material for: An open-source FACS automation system for high-throughput cell biology
Source: PLoS One. 2024 Mar 21;19(3):e0299402. doi: 10.1371/journal.pone.0299402 (PMC10956866; doi:10.1371/journal.pone.0299402)
Supplement: S3 File — (PDF) [file pone.0299402.s007.pdf]

Electronics BOM

Item	Quantity	Part number	Vendor	Name	Description
SMD291SNL	1	SMD291SNL-ND	Digikey	SOLDER PASTE NO-CLEAN LF 5CC SYR	High-temperature solder paste
SMDLTLFP10T5	1	SMDLTLFP10T5-ND	Digikey	SOLDER PASTE LOW TEMP LF T5 10CC	Low-temperature solder paste
**5-0004 FACS Automation PCB**	1	**5-0004**	**OSH Park**	**FACS Automation PCB**	PCB for controlling motors, encoders, cooling, and thermistors.
5-0004 FACS Automation PCB Stencil	1	--	OSH Stencil	FACS Automation PCB Stencil	Solder paste stencil for FACS Automation PCB
C1, C2, C3, C4, C9, C10, C11, C12, C17, C18, C19, C	18	1276-1869-1-ND	Digikey	CAP CER 10UF 25V X5R 0603	General 10uF capacitors
C5, C6, C7, C8, C13, C14, C15, C16, C21, C22, C23,	18	1276-1935-1-ND	Digikey	CAP CER 0.1UF 50V X7R 0603	General 0.1uF capacitors
D1	1	US1A-FDICT-ND	Digikey	DIODE GEN PURP 50V 1A SMA	Diode
J5, J6	2	A1411-ND	Digikey	CONN PLUG HSG 2POS 5.08MM	Power + valve connector
#J5, #J6	2	A14367-ND	Digikey	CONN HEADER VERT 2POS 5.08MM	Power + valve header
#J5, #J6	4	A25555-ND	Digikey	CONN SOCKET 18-24AWG CRIMP TIN	Power + valve crimps
#J6	1	839-1291-ND	Digikey	CONN PWR JACK 2.1X5.5MM SOLDER	Power plug
J7, J8, J10, J11, J13, J14	6	732-5443-ND	Digikey	CONN RCPT 6POS IDC 28AWG GOLD	2x03 headers
#J7, #J8, #J10, #J11, #J13, #J14	6	732-5394-ND	Digikey	CONN HEADER VERT 6POS 2.54MM	2x03 connectors
J9, J12	2	732-5444-ND	Digikey	CONN RCPT 8POS IDC 28AWG GOLD	2x04 headers
#J9, #J12	2	732-5395-ND	Digikey	CONN HEADER VERT 8POS 2.54MM	2x04 connectors
J15	1	S9171-ND	Digikey	CONN HEADER VERT 16POS 2.54MM	2x08 headers
#J15	1	S9288-ND	Digikey	CONN HEADER R/A 16POS 2.54MM	2X08 connectors
P1	1	SAM15923-ND	Digikey	CONN HEADER SMD 36POS 2.54MM	2x18 header
P2, P3, P4, P6, P7	5	WM14828-ND	Digikey	CONN HEADER SMD 8POS 2.54MM	1x8 header
P5	1	WM8617-ND	Digikey	CONN HEADER SMD 4POS 2.54MM	1x4 header
Q1	1	IRLML2803PBFCT-ND	Digikey	MOSFET N-CH 30V 1.2A SOT23	Solenoid valve MOSFET
TP1, TP2, TP3	3	36-5006-ND	Digikey	PC TEST POINT COMPACT BLACK	Test points
R1	1	RMCF0805FT330RCT-ND	Digikey	RES 330 OHM 1% 1/8W 0805	330 Ohm resistor
R2, R3, R4, R5, R6, R7, R8, R9, R10, R11, R12, R13,	20	RNCP0603FTD10K0CT-ND	Digikey	RES 10K OHM 1% 1/8W 0603	10k Ohm resistor
R21, R22, R23, R25, R33, R34, R35, R36, R45, R46,	18	YAG1235CT-ND	Digikey	RES SMD 100K OHM 0.1% 1/10W 0603	100k Ohm resistor
R29, R30, R31, R32, R41, R42, R43, R44, R53, R54, R55, R56, R63, R64, R65, R72, R73, R74	18	CSR0603FKR500CT-ND	Digikey	RES 0.5 OHM 1% 1/8W 0603	500mOhm resistor
U1, U2, U3, U4, U5, U6, U7, U8, U9, U10, U11, U12, U	18	MAX14870ETC+CT-ND	Digikey	IC MOTOR DRIVER 4.5V-36V 12TDFN	Motor drivers
DB25-1, DB25-2	2	2057-DB25-SF-M1-ND	Digikey	CONN D-SUB RCPT 25POS IDC	DSUB25 connectors
#DB25-1, #DB25-2	2	2057-DB25-PD-ND	Digikey	2057-DB25-PD-ND	DSUB25 headers
DB15	1	215ME-ND	Digikey	CONN D-SUB PLUG 15POS PNL MNT	DSUB15 connectors
#DB15	1	AFR15B-ND	Digikey	CONN D-SUB RCPT 15POS IDC	DSUB15 headers
--	3	3M157833-1-ND	Digikey	CBL RIBN 26COND 0.05 GRAY 1'	Shielded ribbon cables
#TH1, #TH2, #TH3, #TH4, #TH5, #TH6	6	615-1146-ND	Digikey	THERMISTOR NTC 10KOHM 3575K BEAD	Thermistors
--	1	839-1291-ND	Digikey	CONN PWR JACK 2.1X5.5MM SOLDER	Power barrel jack connector
**5-0007 FACS Photointerruptor Breakout Board**	1	**5-0007**	**OSH Park**	**FACS Photointerruptor Breakout Board**	PCB for socket encoders
5-0007 FACS Photointerruptor Breakout Board	1	--	OSH Stencil	FACS Photointerruptor Breakout Board Ster	Solder paste stencil for photointerruptor breakout board
U1, U2, U3, U4, U5, U6, U7, U8, U9	18	516-2467-1-ND	Digikey	SENSOR OPT REFLECTIVE 2MM 6SMD	Optical encoders
