# Supplementary material for: An open-source FACS automation system for high-throughput cell biology
Source: PLoS One. 2024 Mar 21;19(3):e0299402. doi: 10.1371/journal.pone.0299402 (PMC10956866; doi:10.1371/journal.pone.0299402)

FACS Automation Controller Software

Click through control of Sony Cell Sorter software

Last Updated: 2023-02-23

Startup sequence

General

Prepare the Cell Sorter Software GUI.

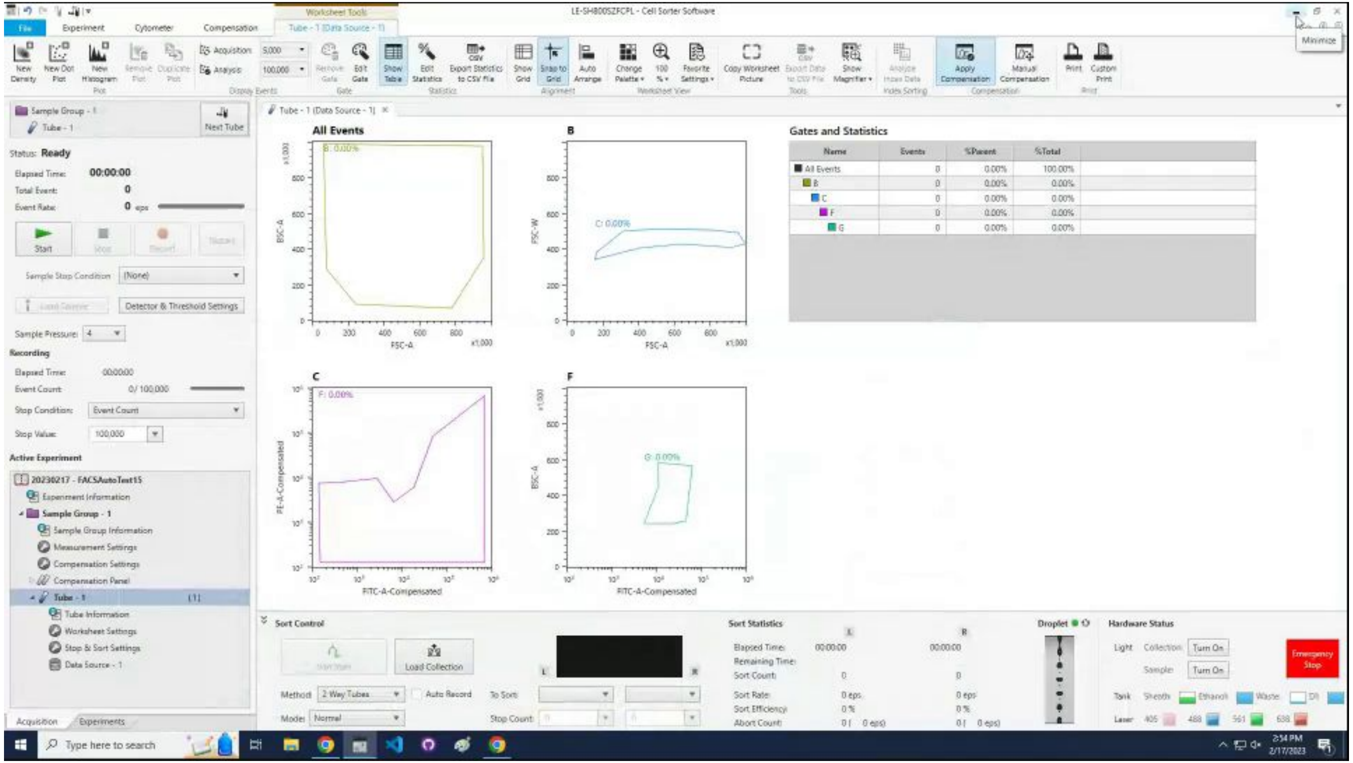

Open the solenoid valve by clicking `cool`.

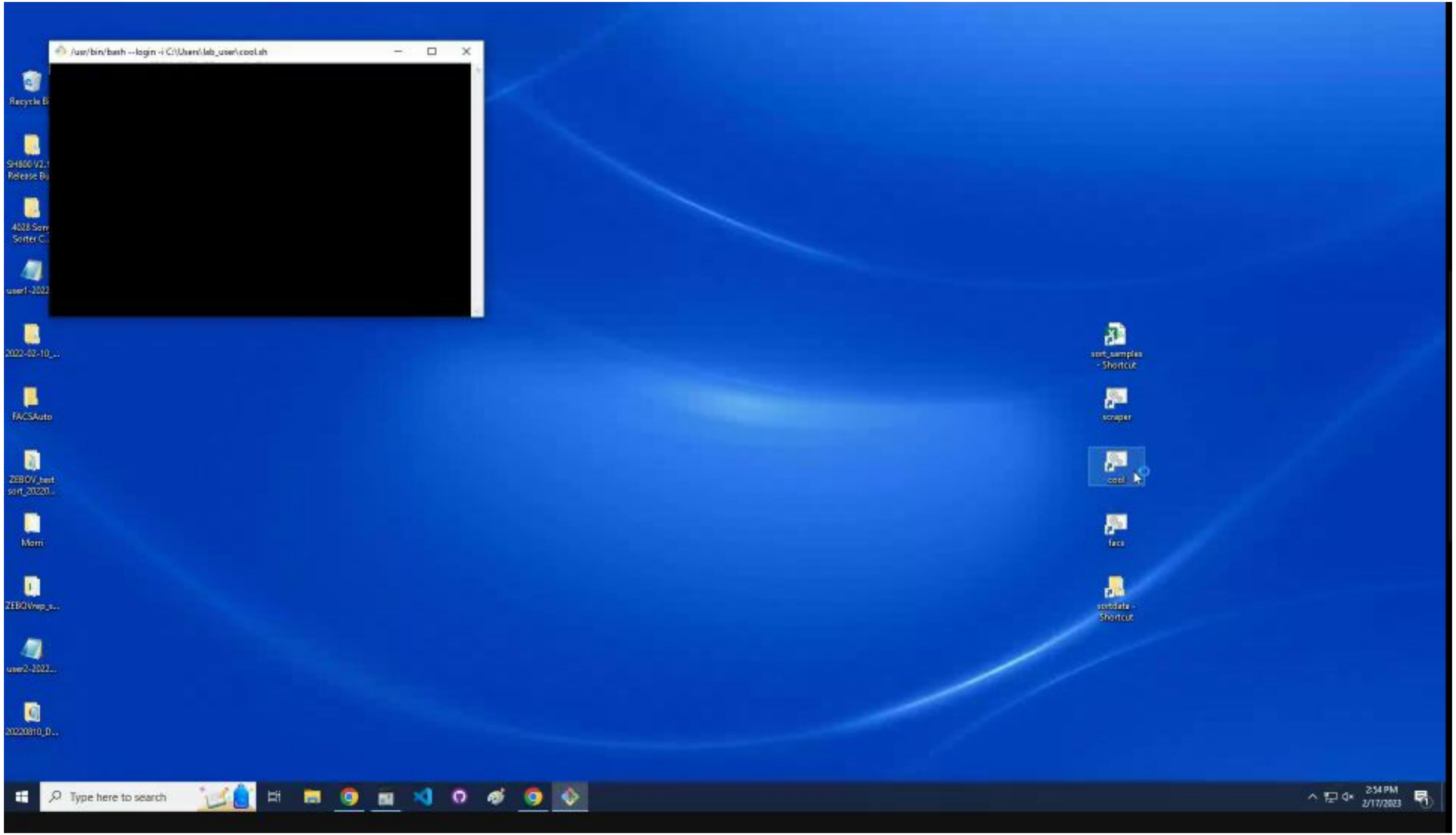

Open the worklist `sort_samples.csv` file.

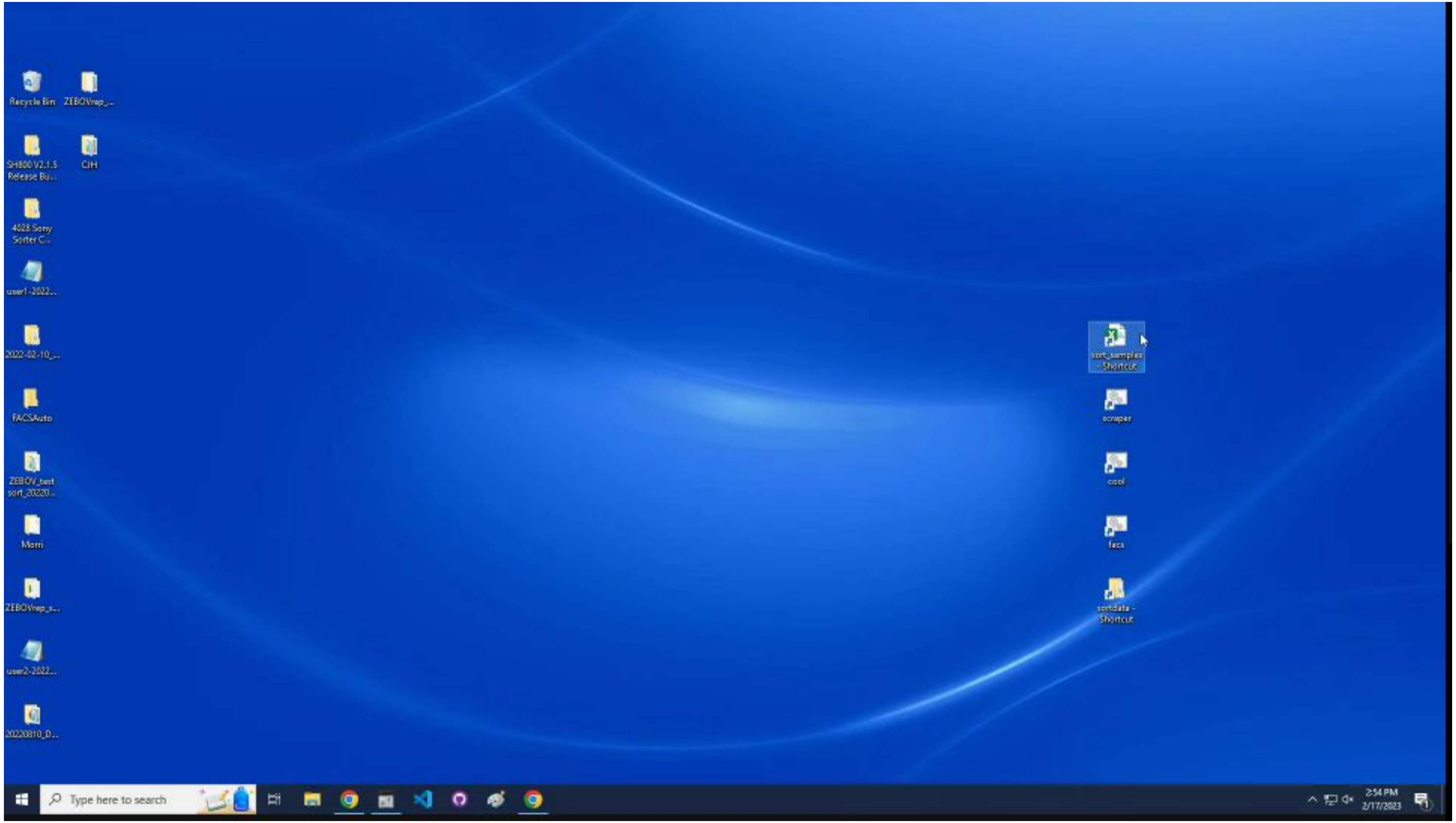

Fill in tube location, sample name, and well ID, and save.

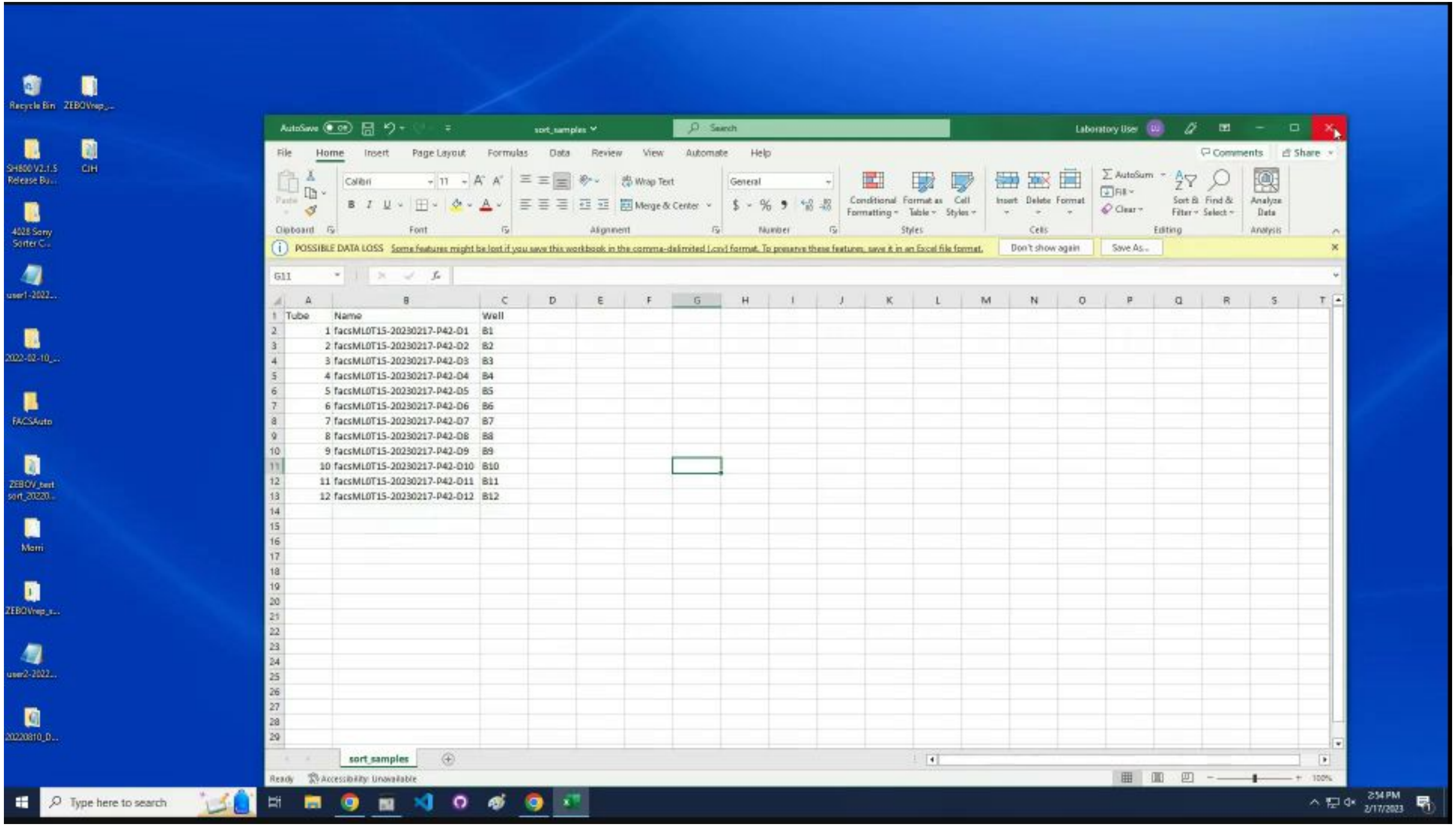

Start the automated sorting by clicking `facs`.

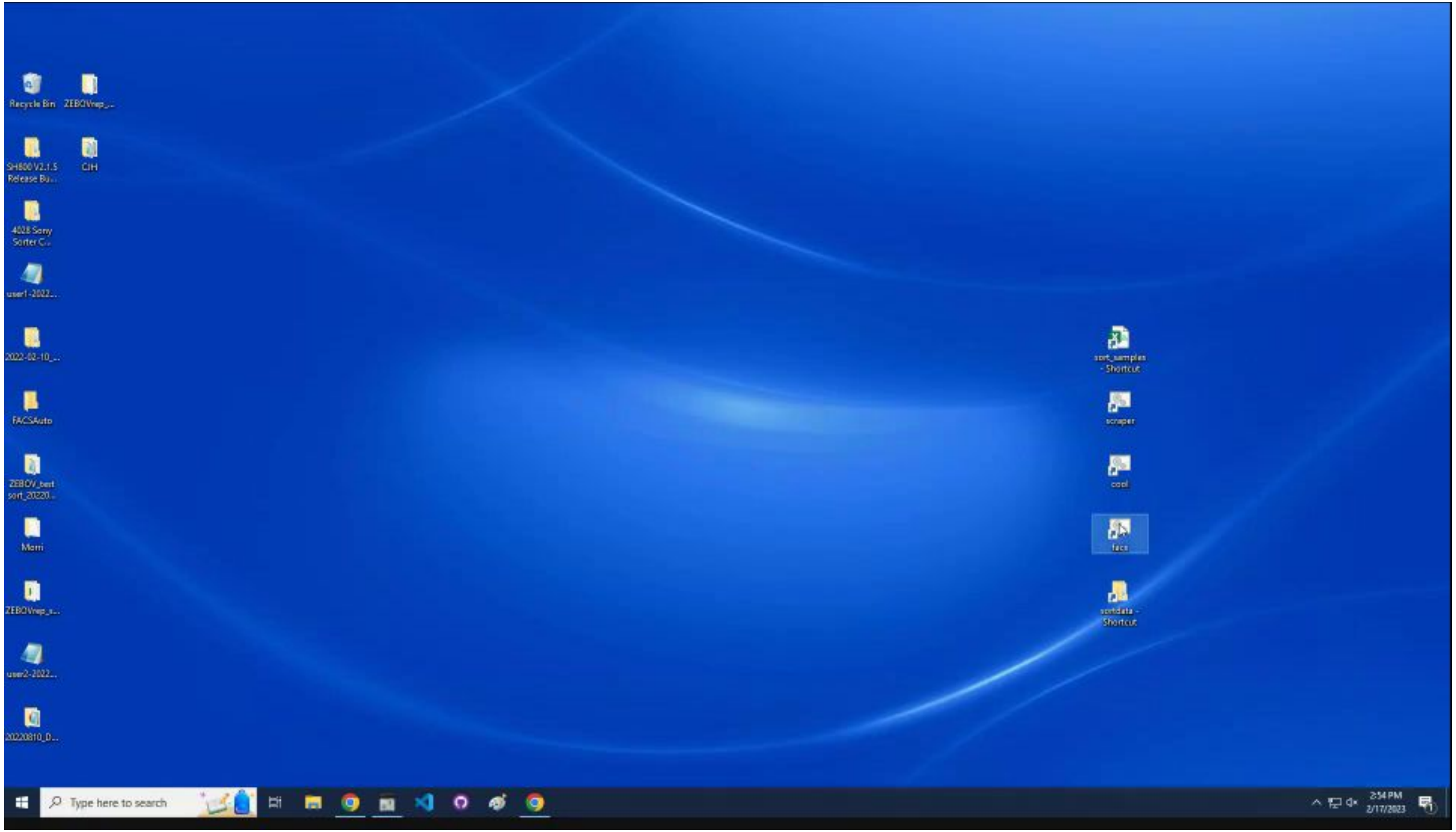

Startup sequence

Need to set up gates

Enter `y` to profile a sample to set up the gates.

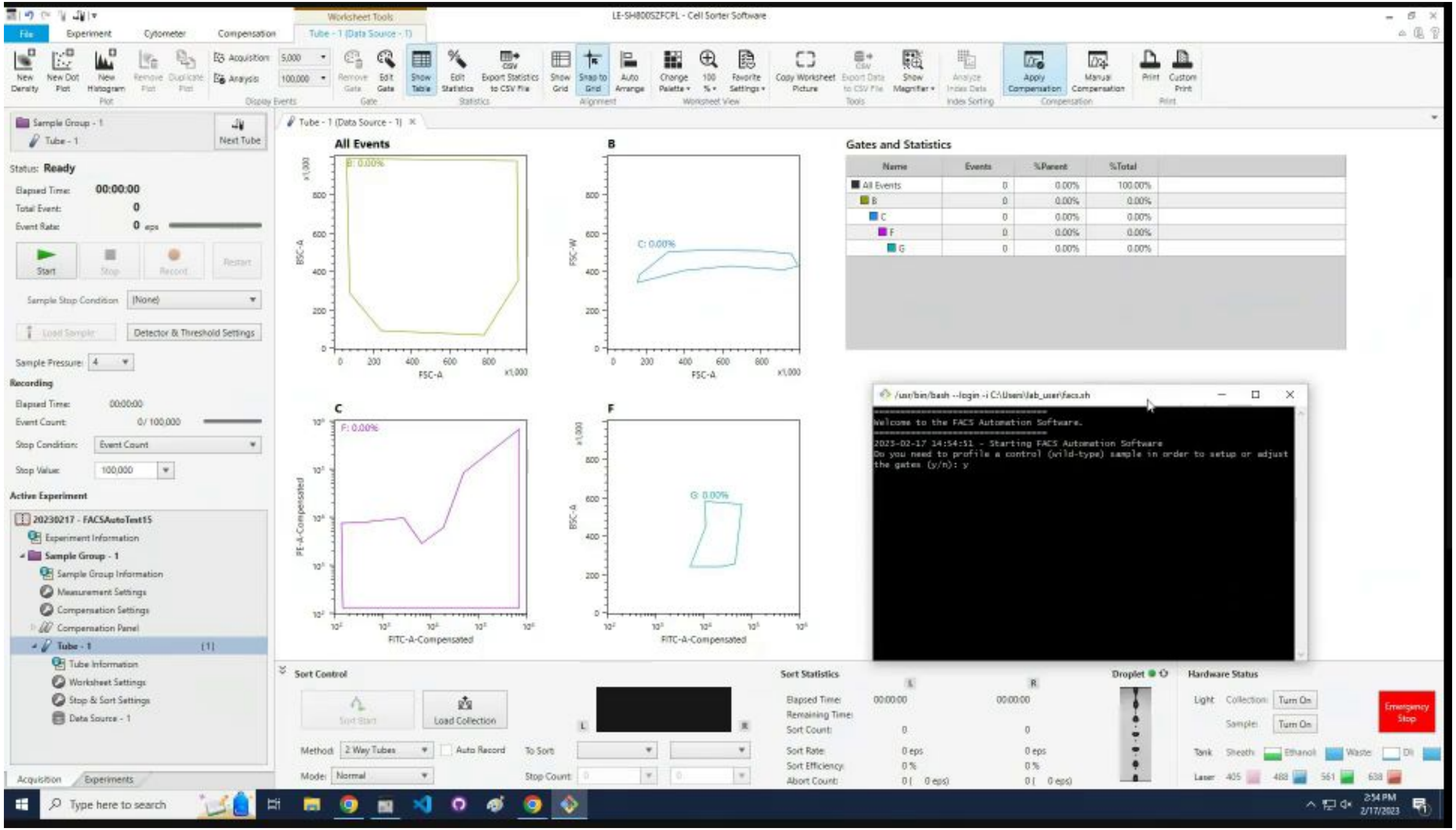

Enter `user name`.

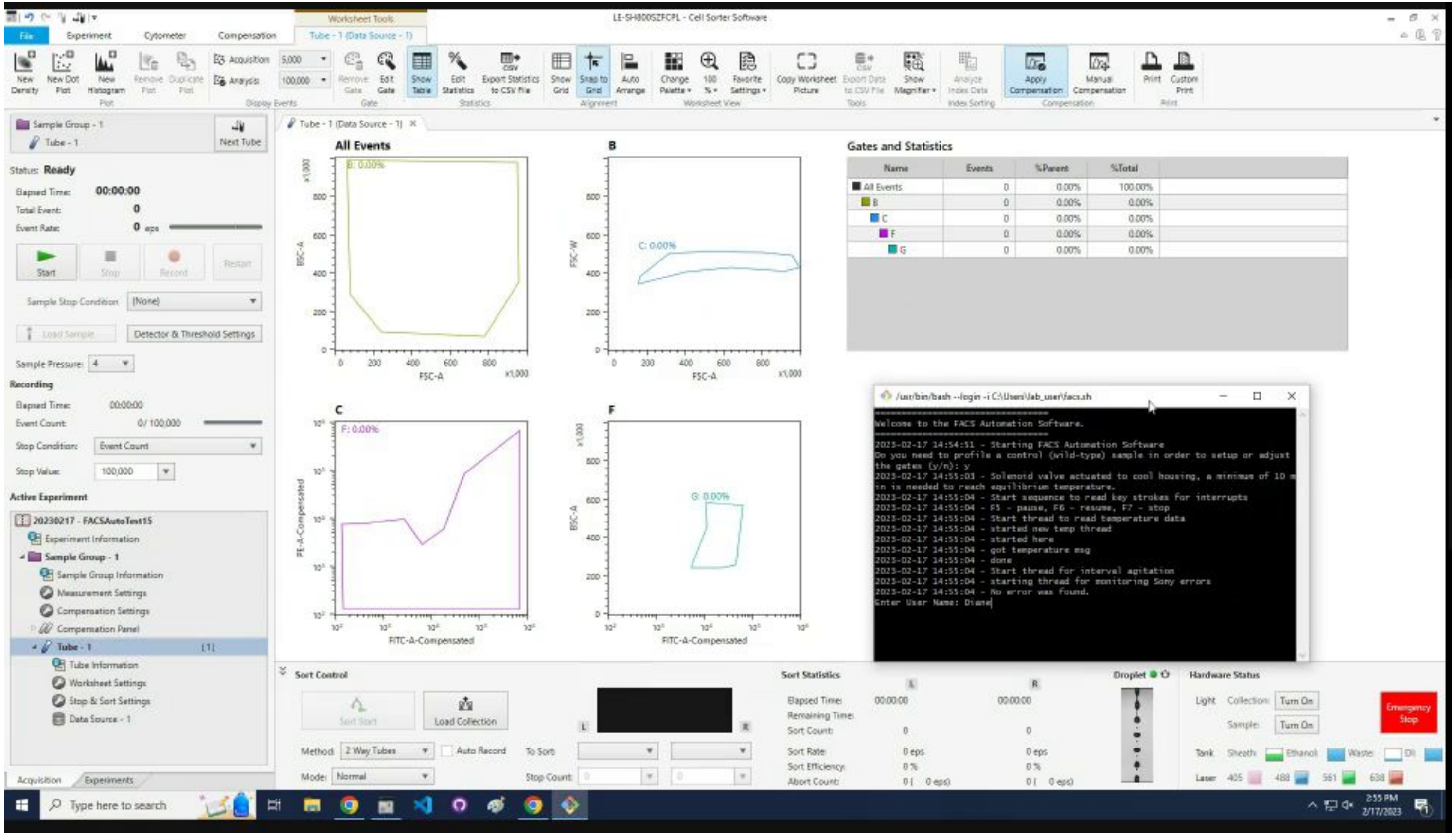

Find experiment name to enter into program.

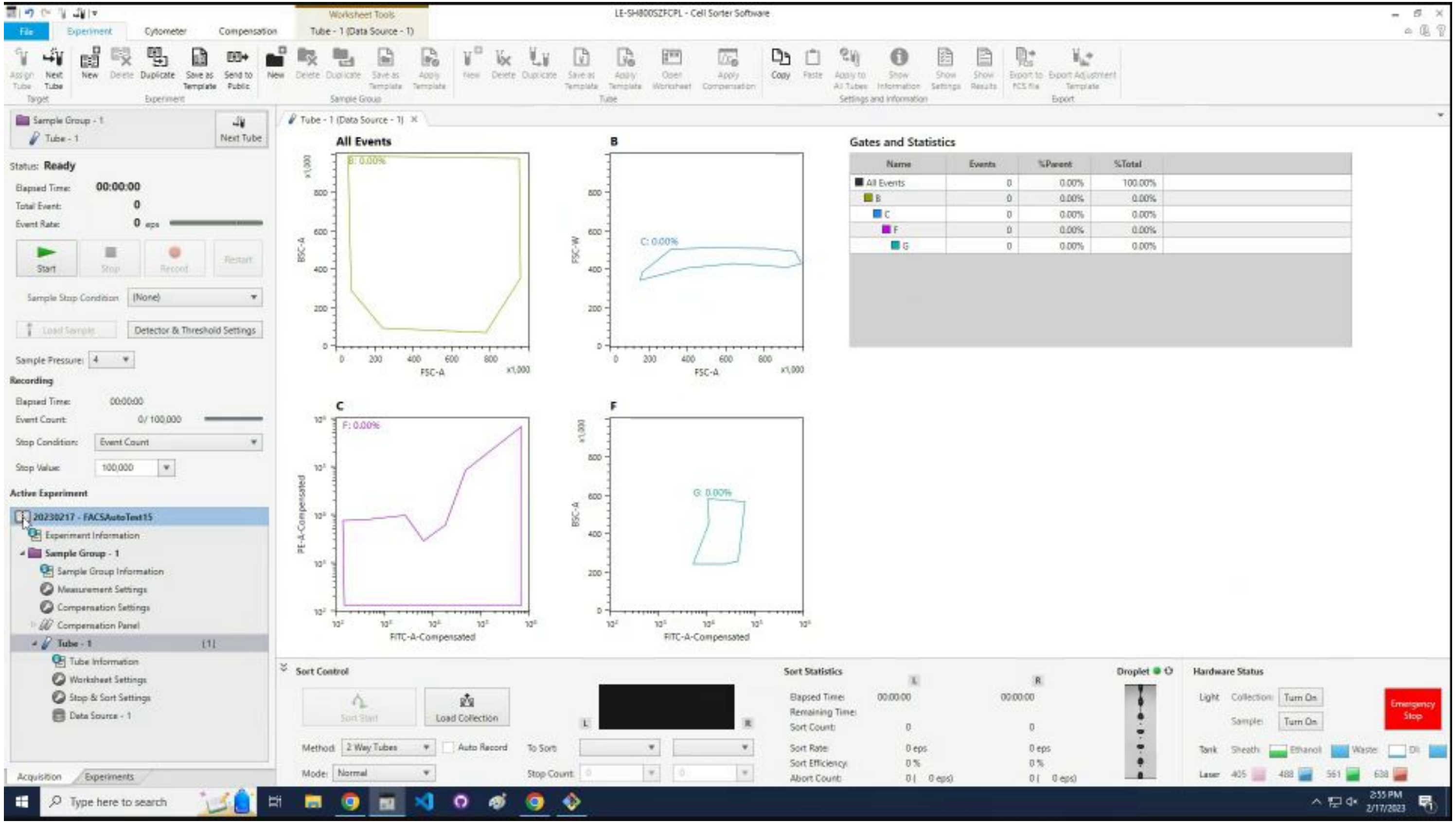

Confirm that the experiment name is correct.

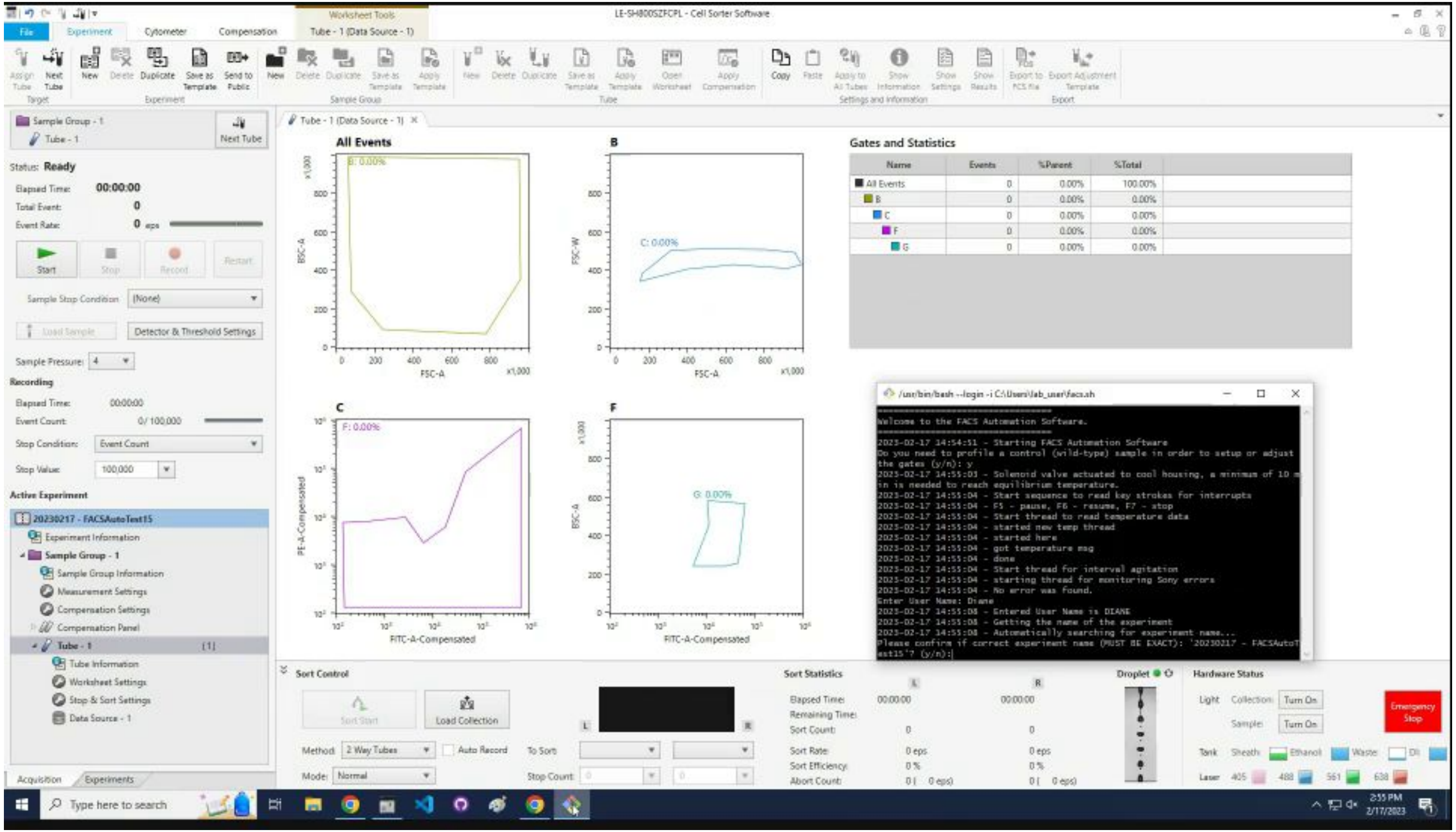

Enter the first tube number to start the sorting process.

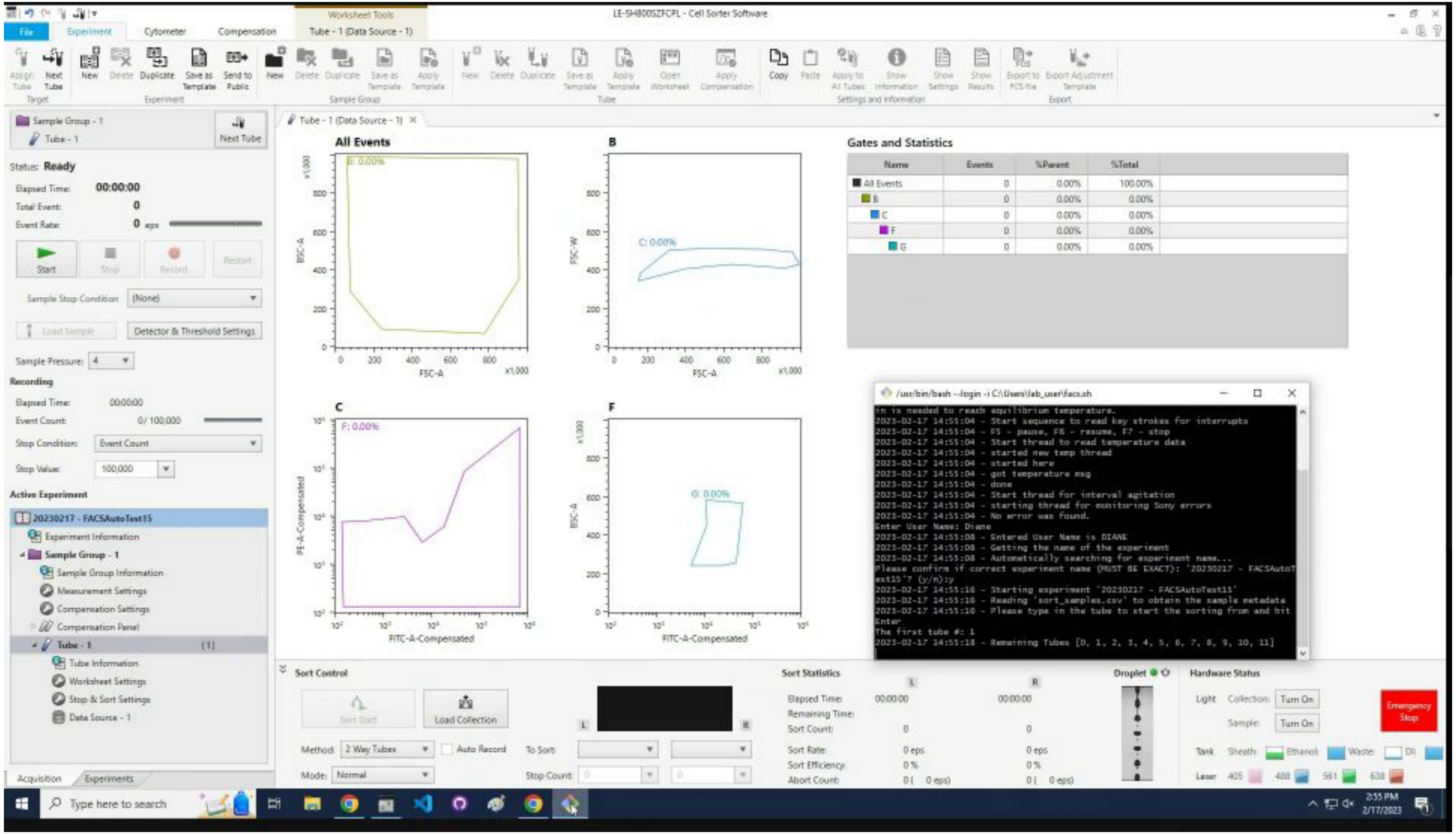

Automated sorting starts to set up the gates.

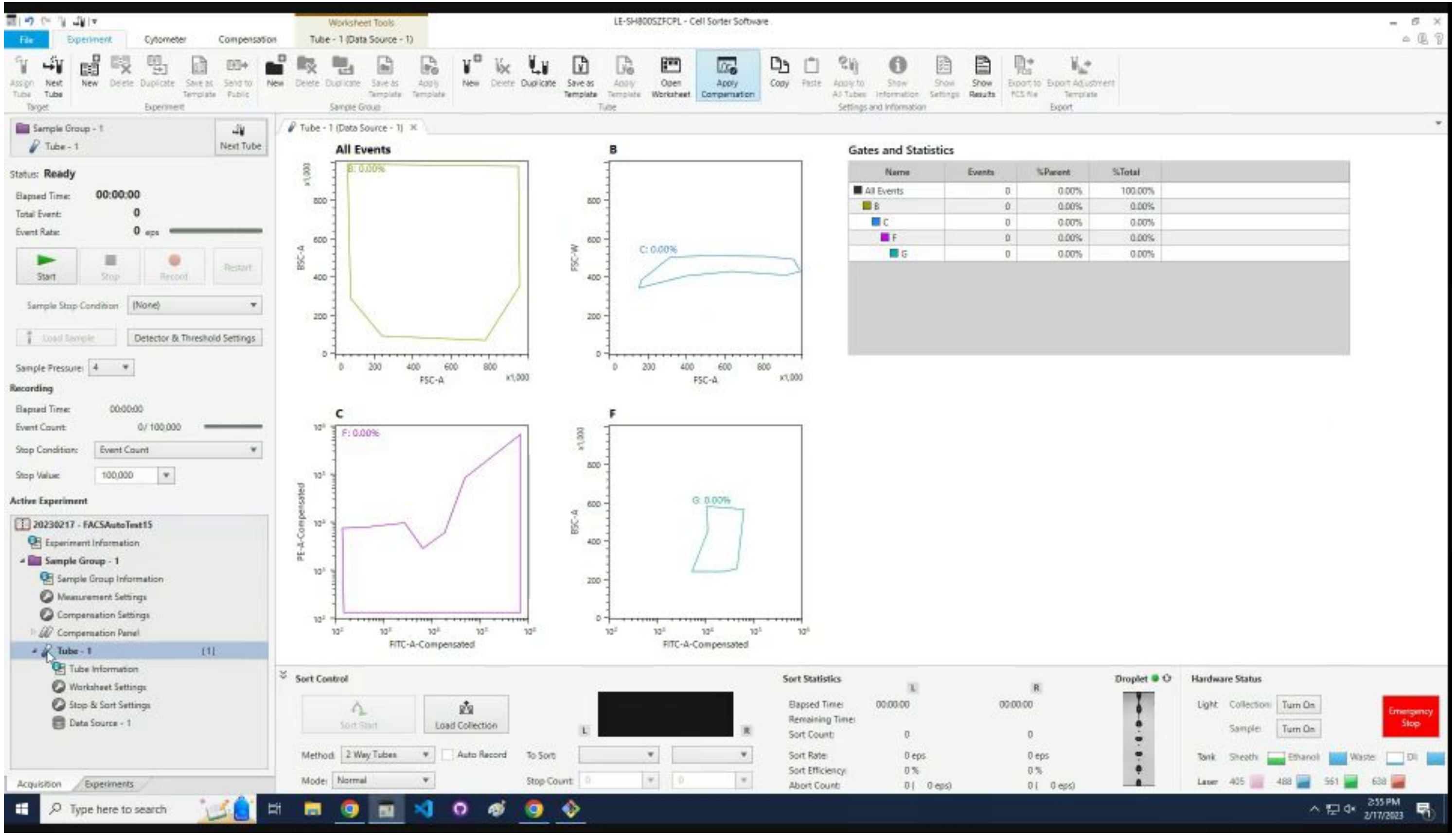

Change the `Stop Value` to `50,000`.

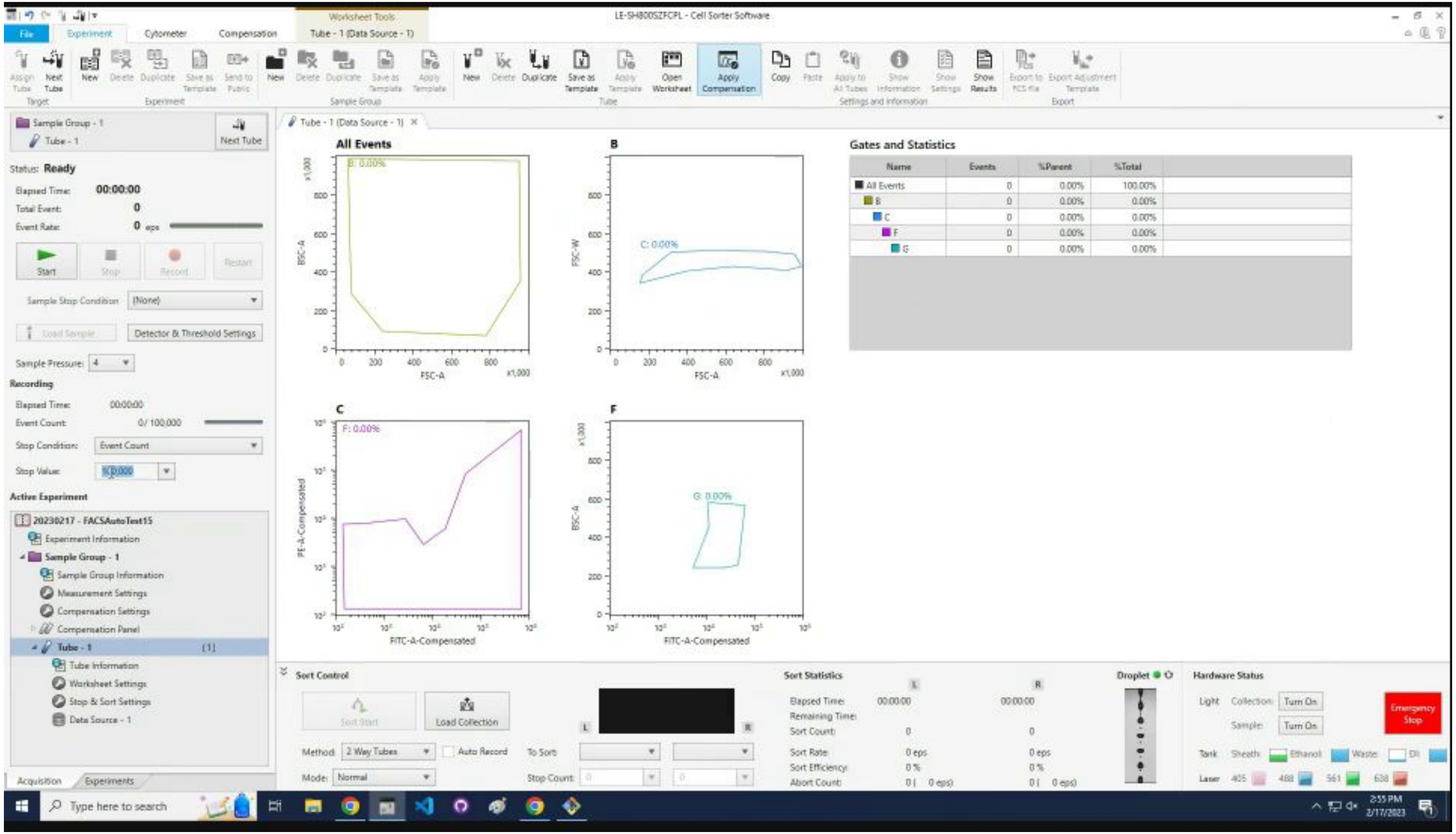

Change the sorting `Method` to `96 Well Plate`.

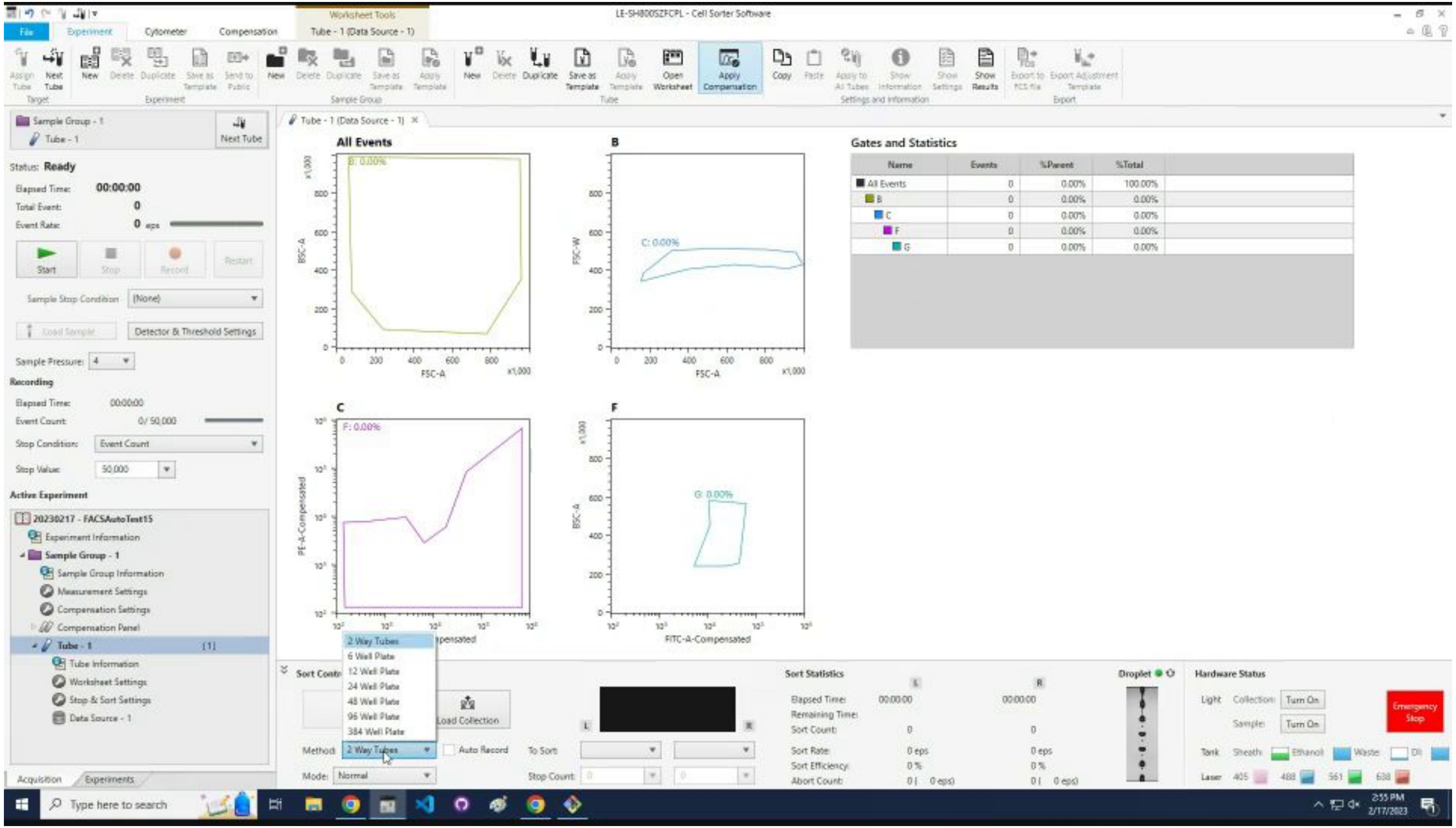

Change the `Stop Condition` to `Event Count`.

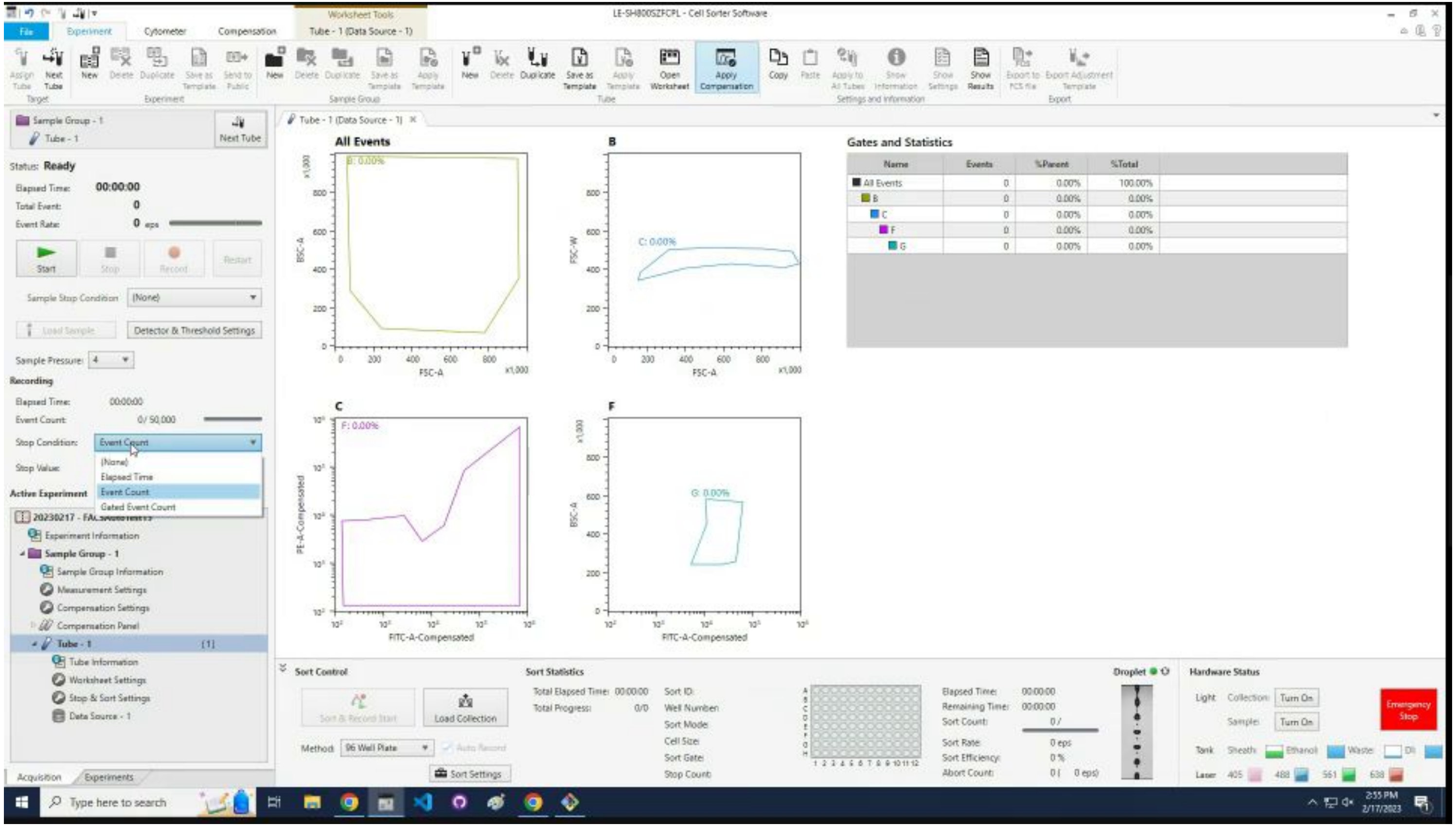

Change the `Sample Pressure` to 5.

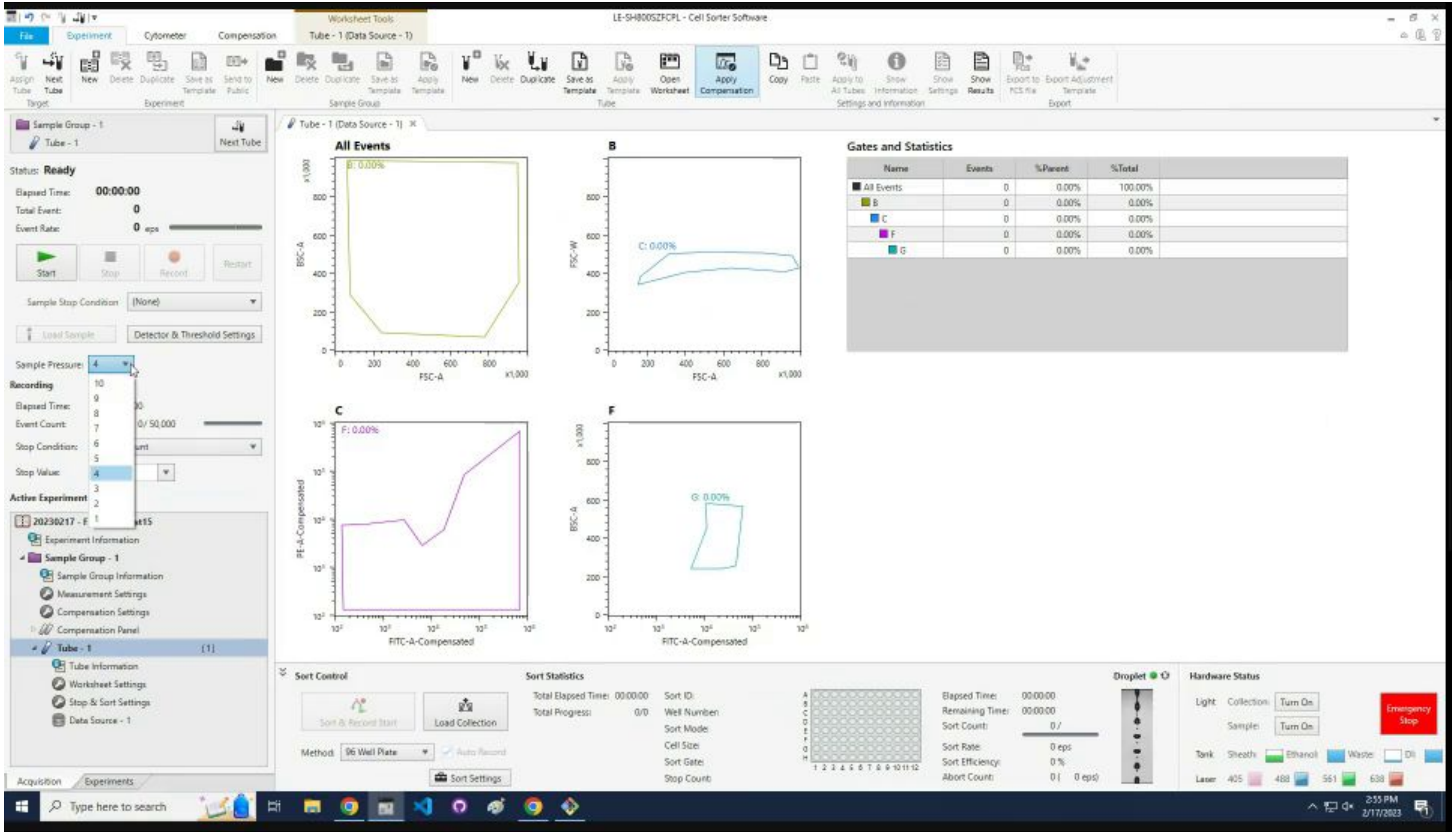

Click `Start` to begin the profile.

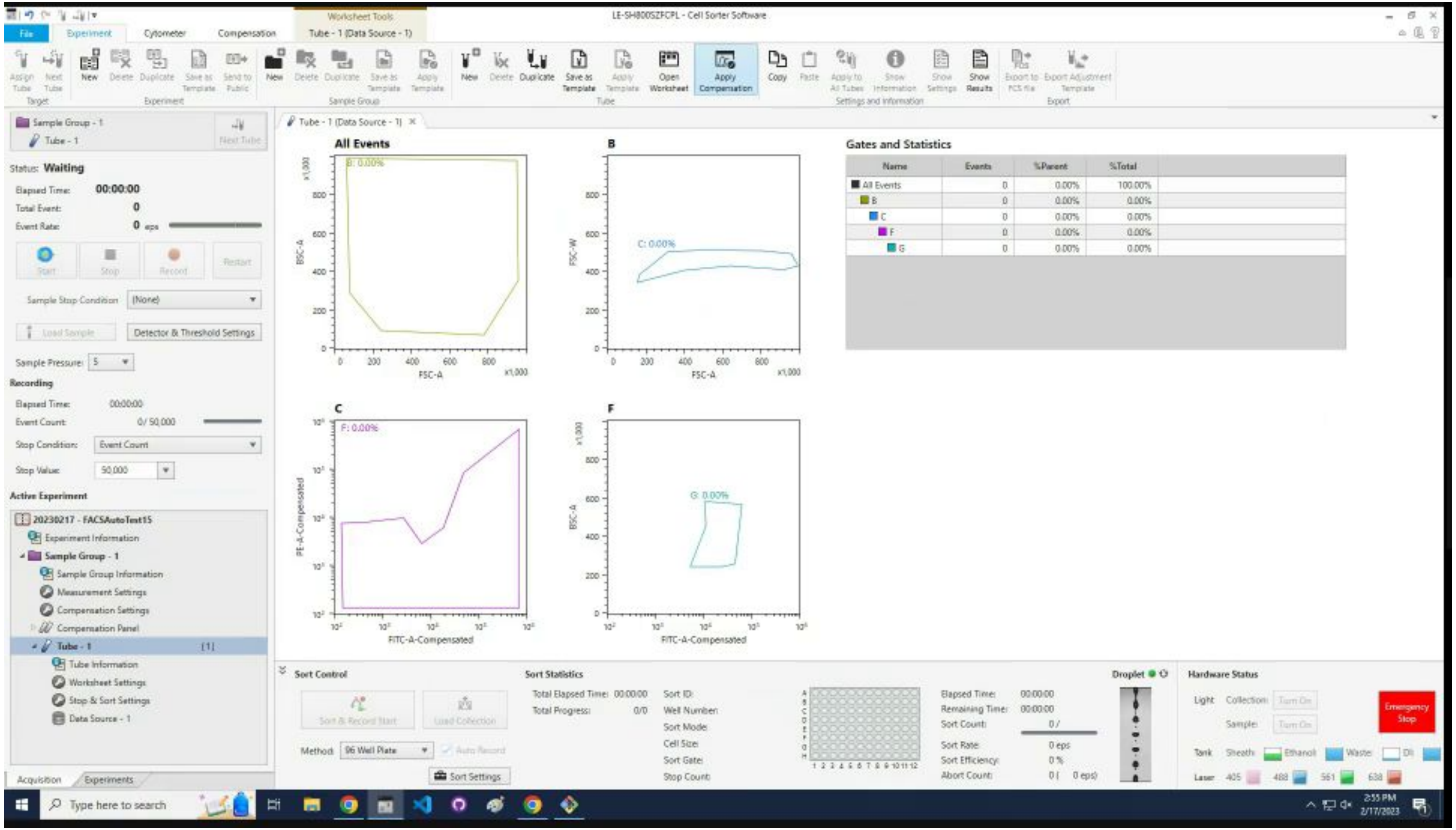

Wait ~20 seconds for cells to appear. Click `Record`.

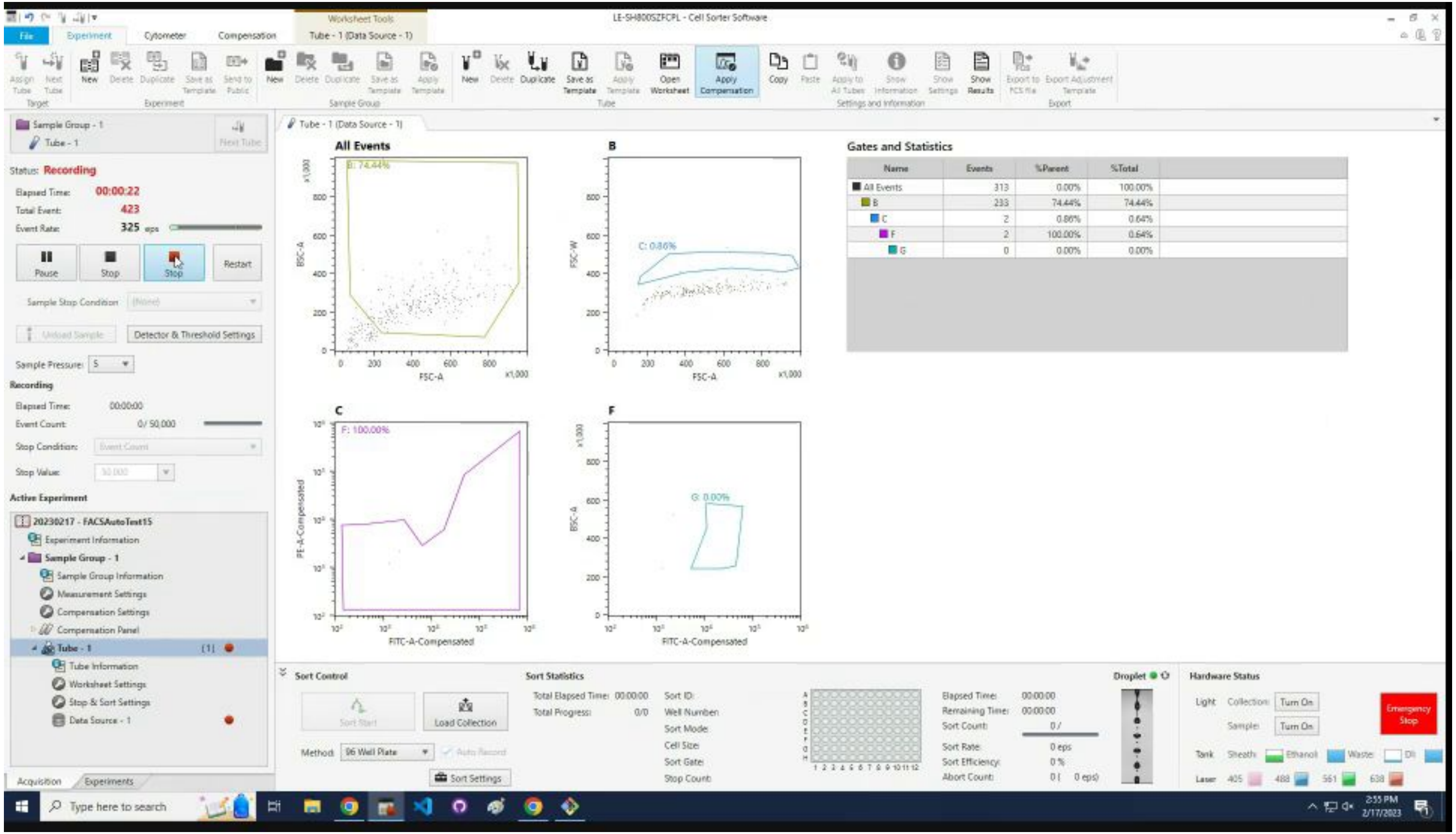

Wait for profiling to finish then click `OK`.

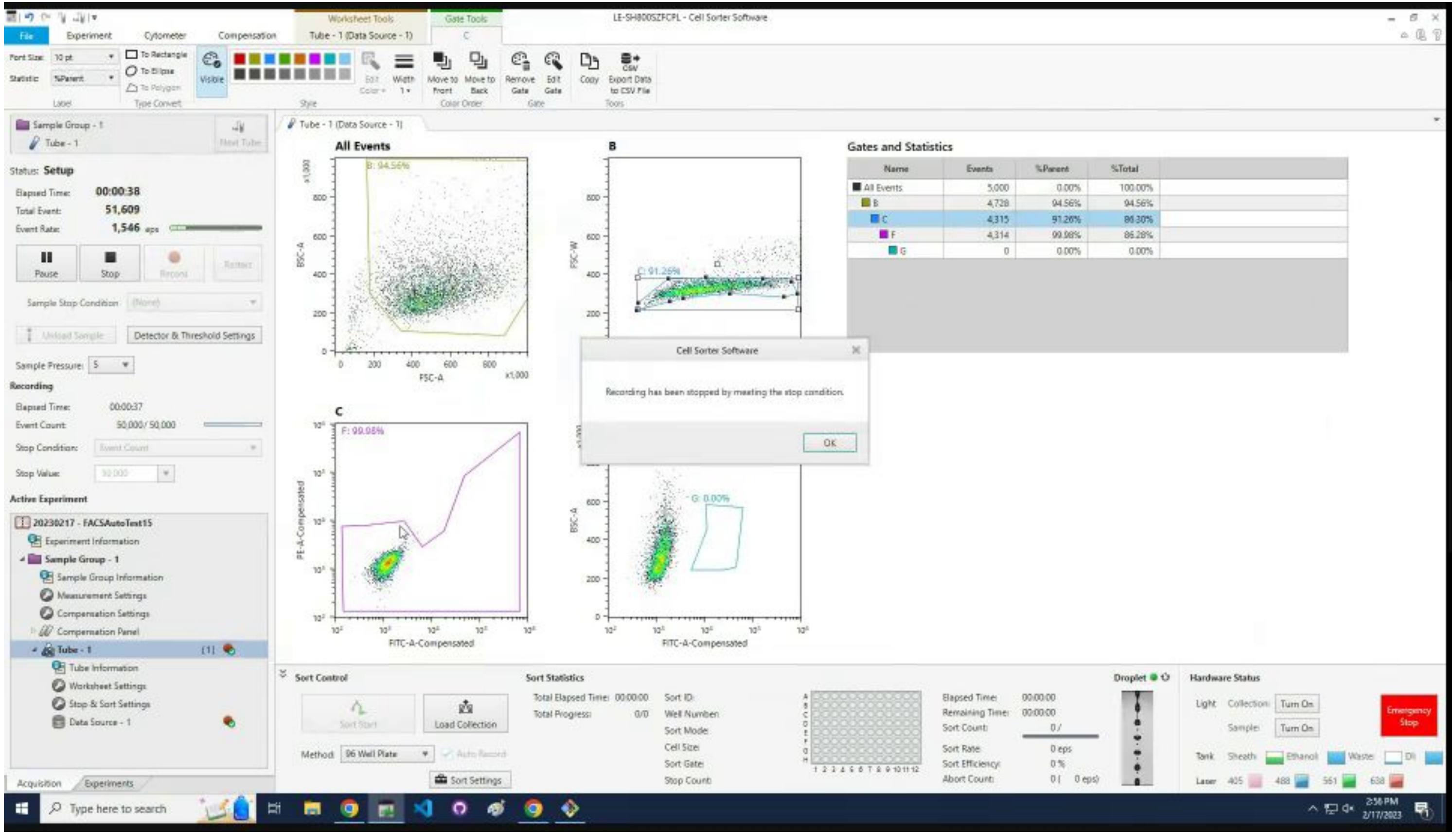

Click `Stop`.

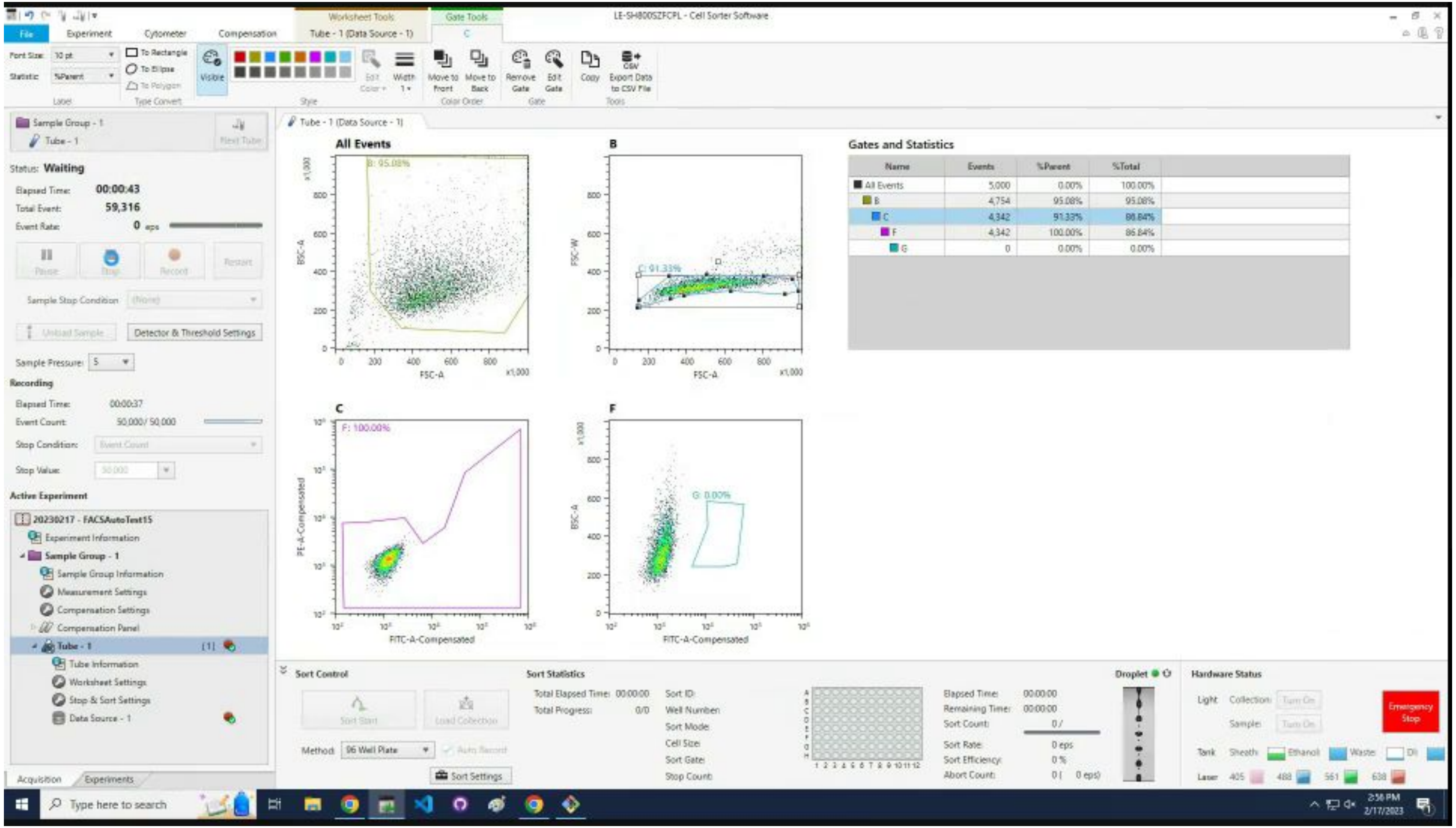

LE-SH800SZFCPL - Cell Sorter Software
Status: Waiting
Elapsed Time: 00:00:43
Total Event: 59,316
Pause
Stop
Record
Restart
Detector & Threshold Settings
Recording
Active Experiment
All Events
Gates and Statistics
Sort Control
Sort Statistics
Hardware Status
Emergency Stop

Adjust the first three gates.

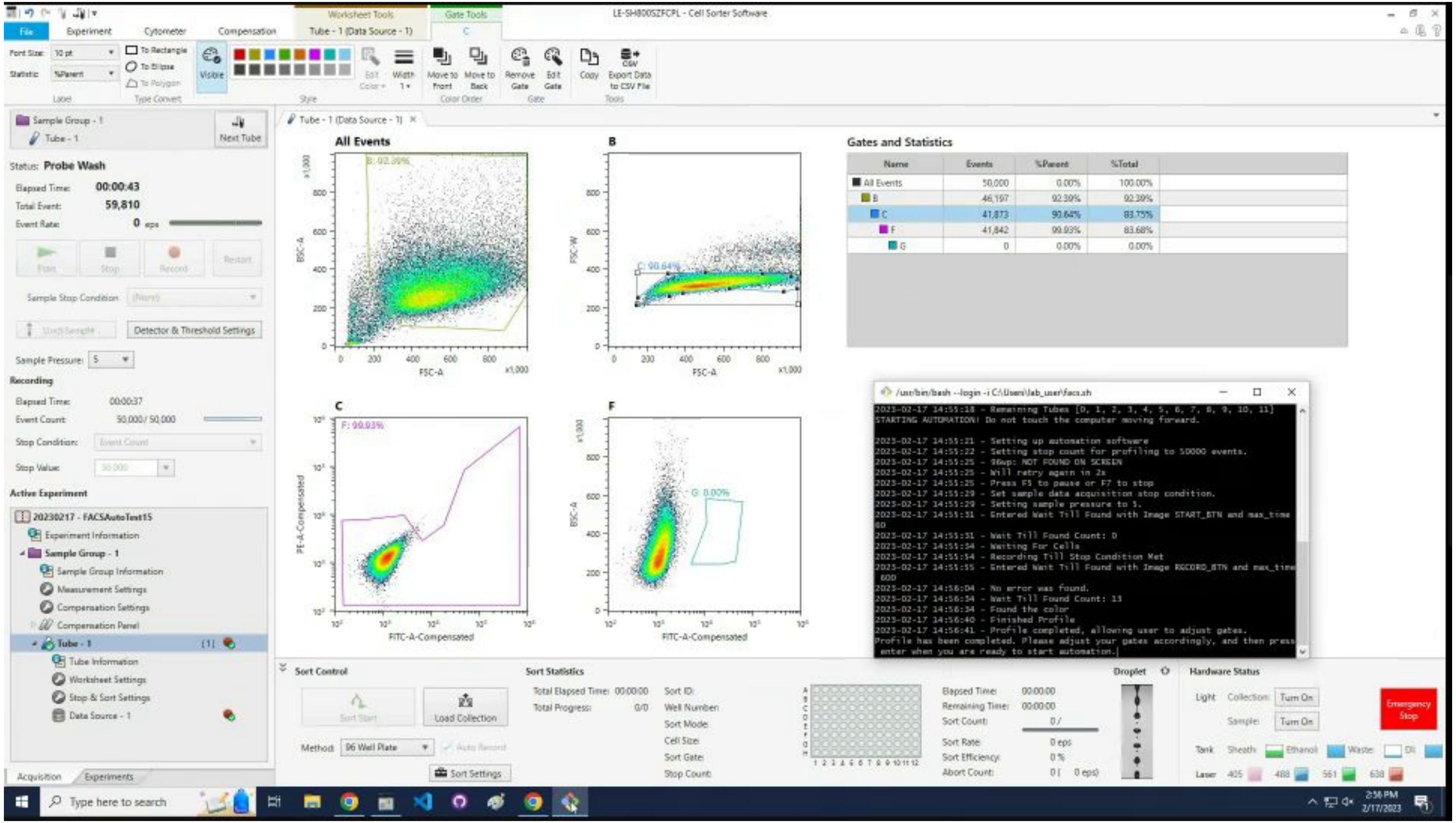

Click the X to close the tube tab.

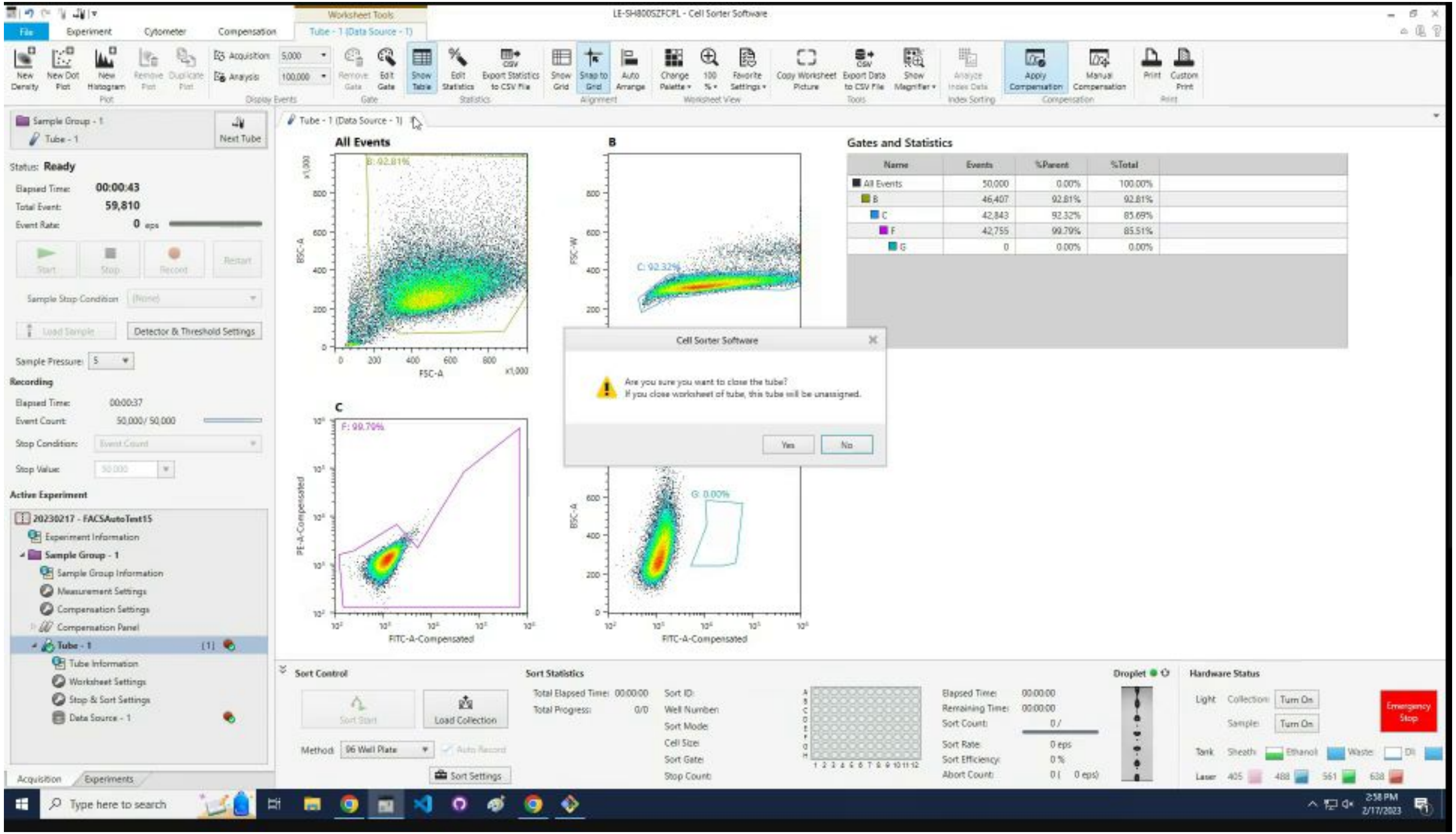

Click `Yes` to confirm closing the tube tab.

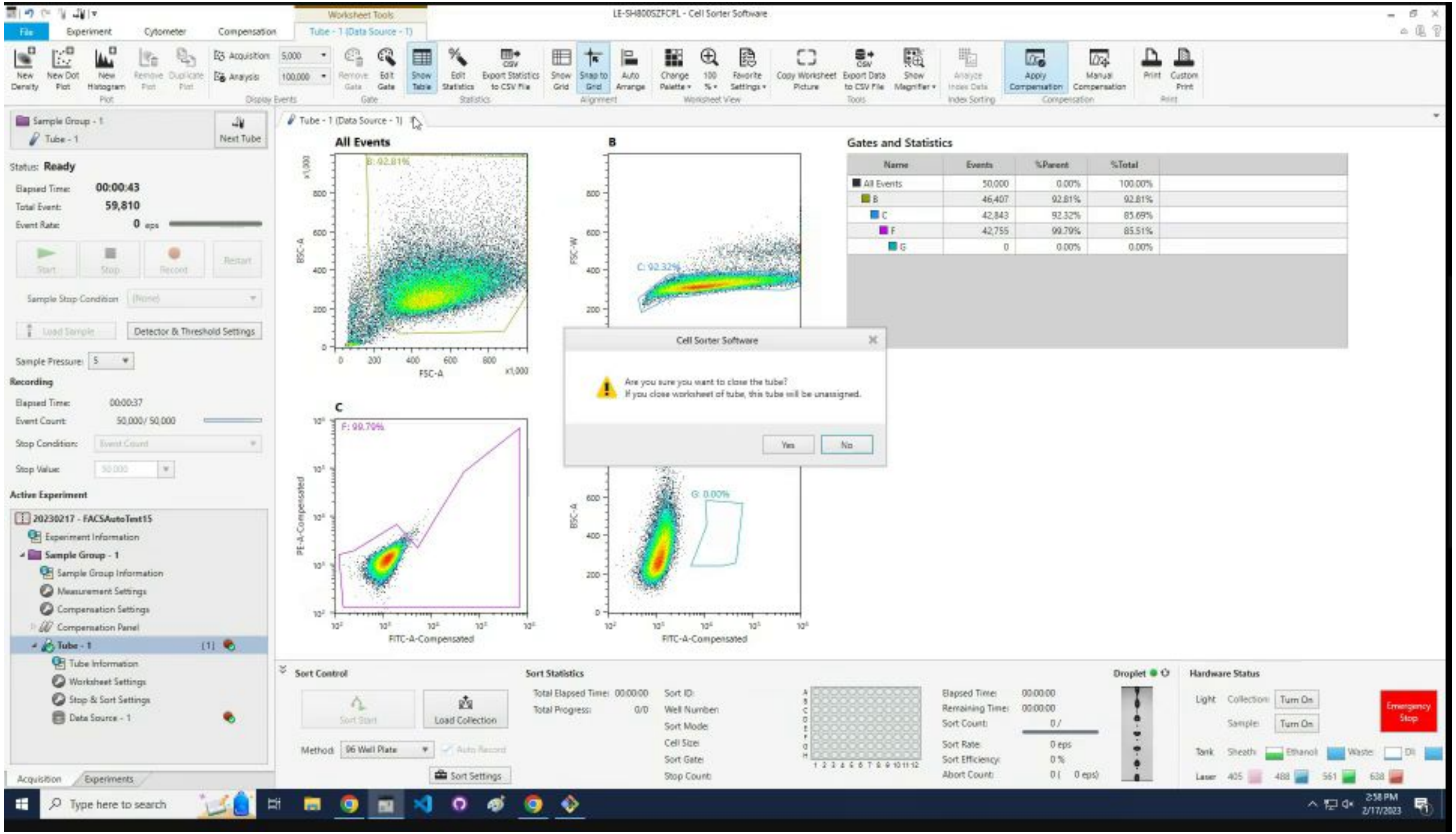

Click on the profile sample in the list.

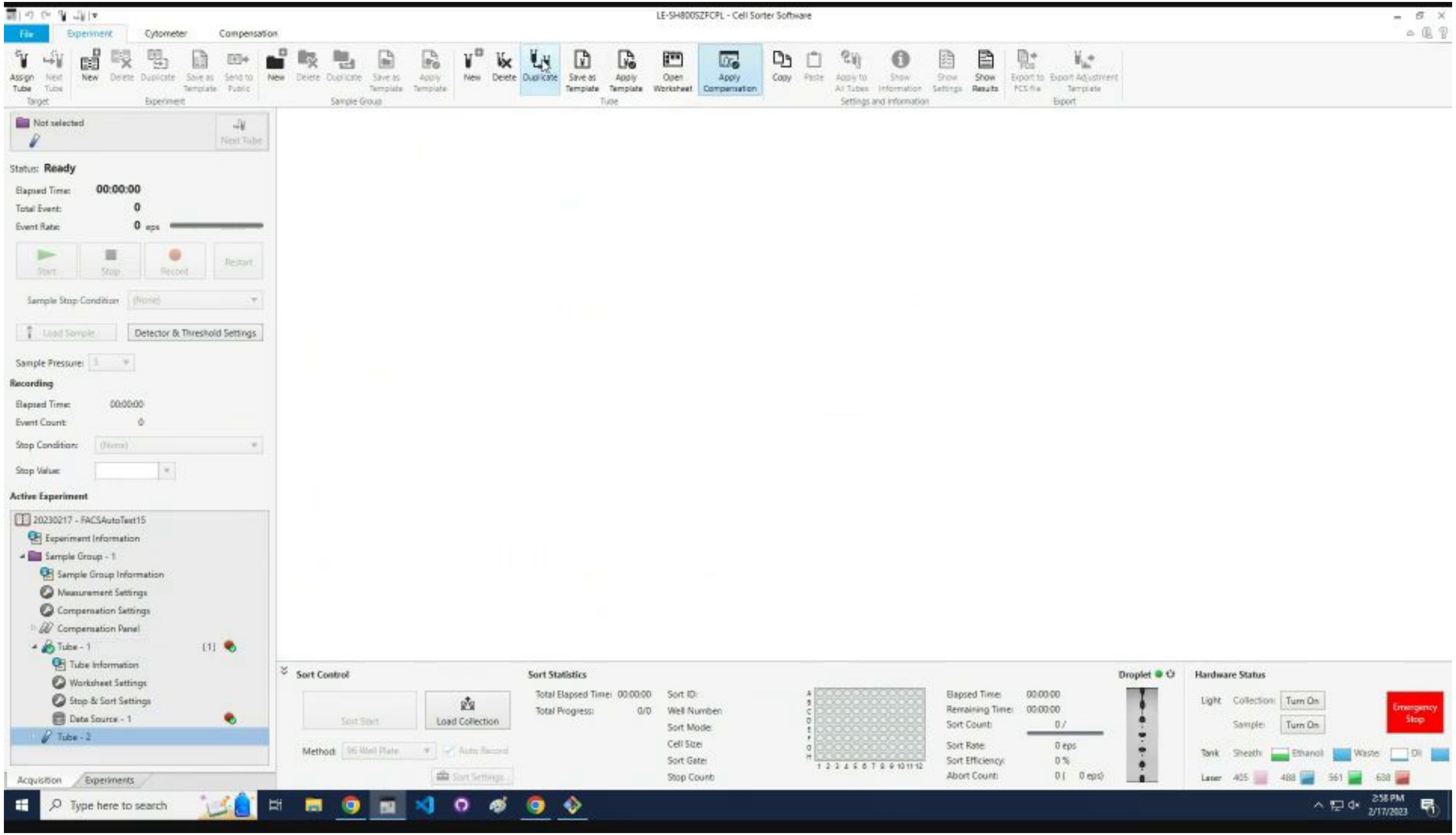

Click the `Duplicate` icon.

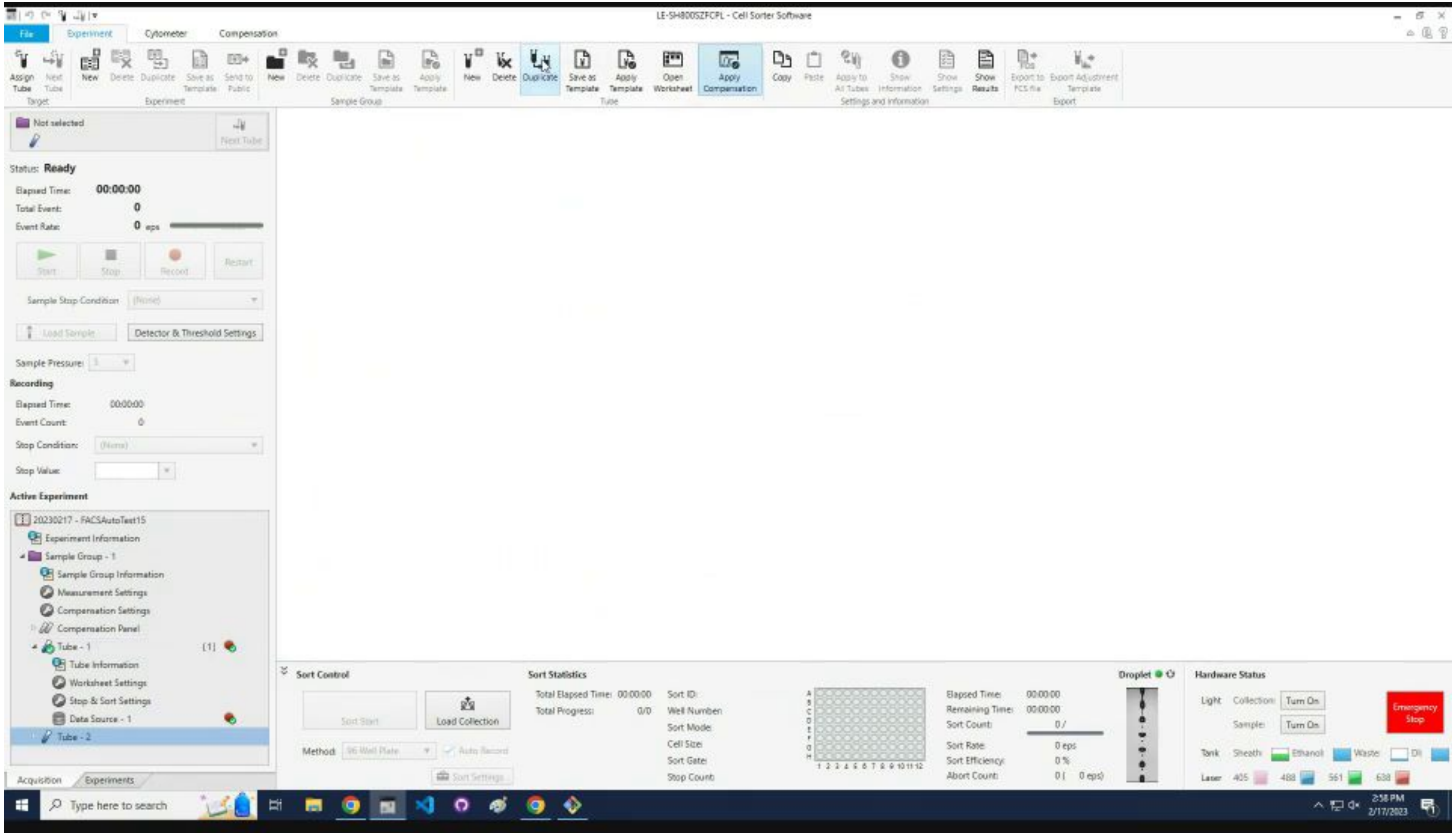

Click the `Assign Tube` icon.

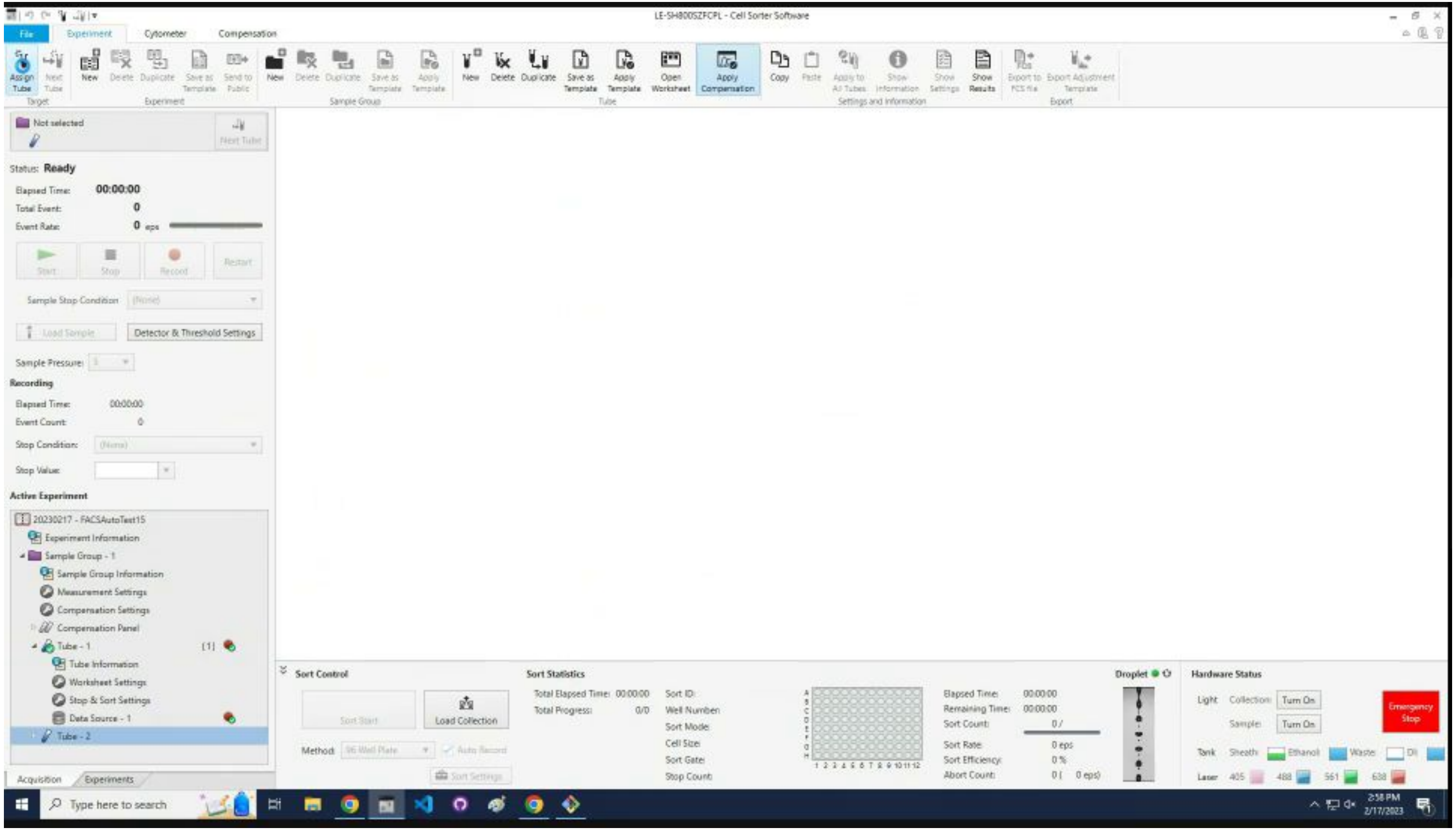

Goto [Iterate through samples] section.

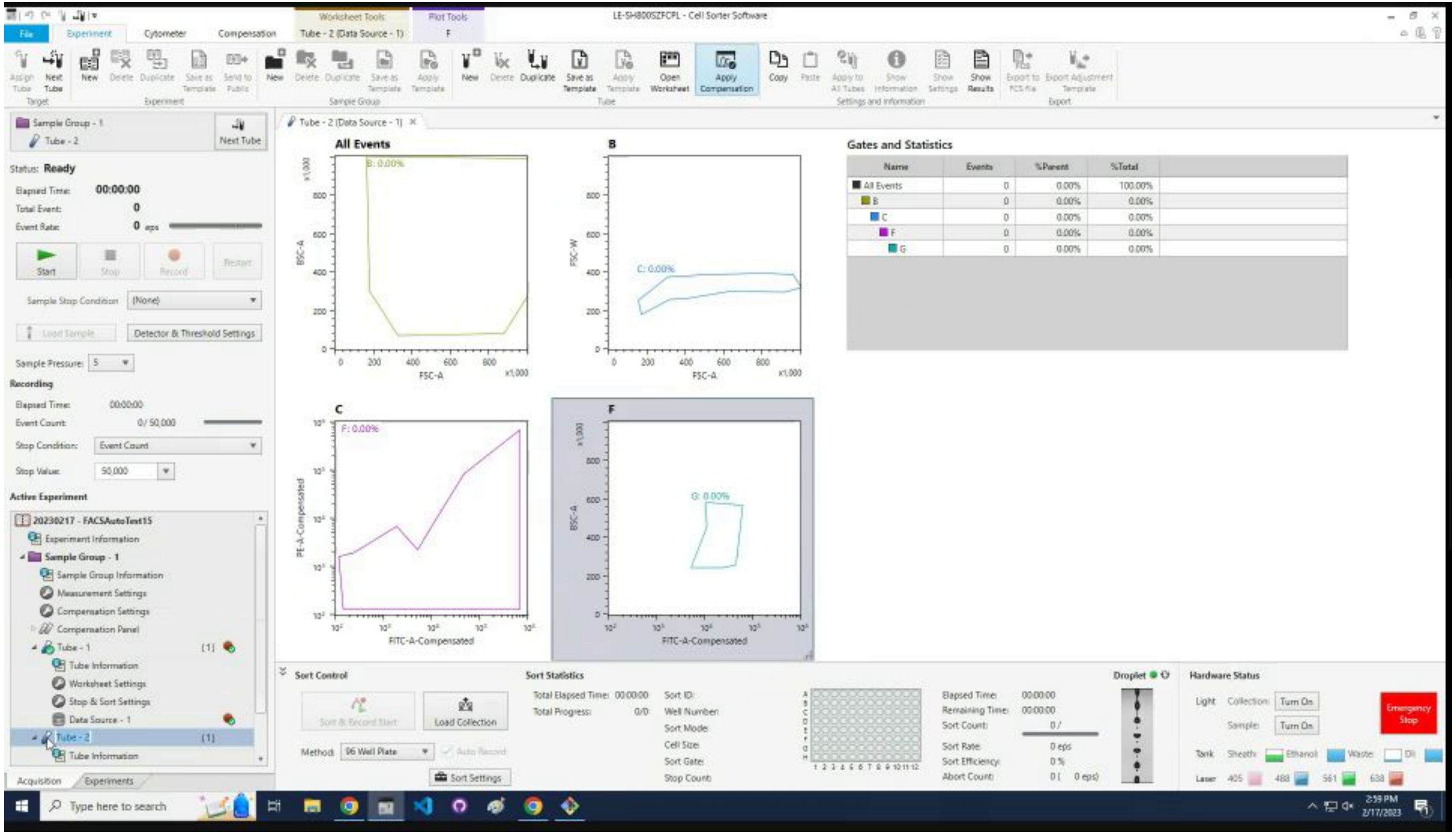

Startup sequence

Continuation from previous set of samples

Enter `n` to not set up the gates.

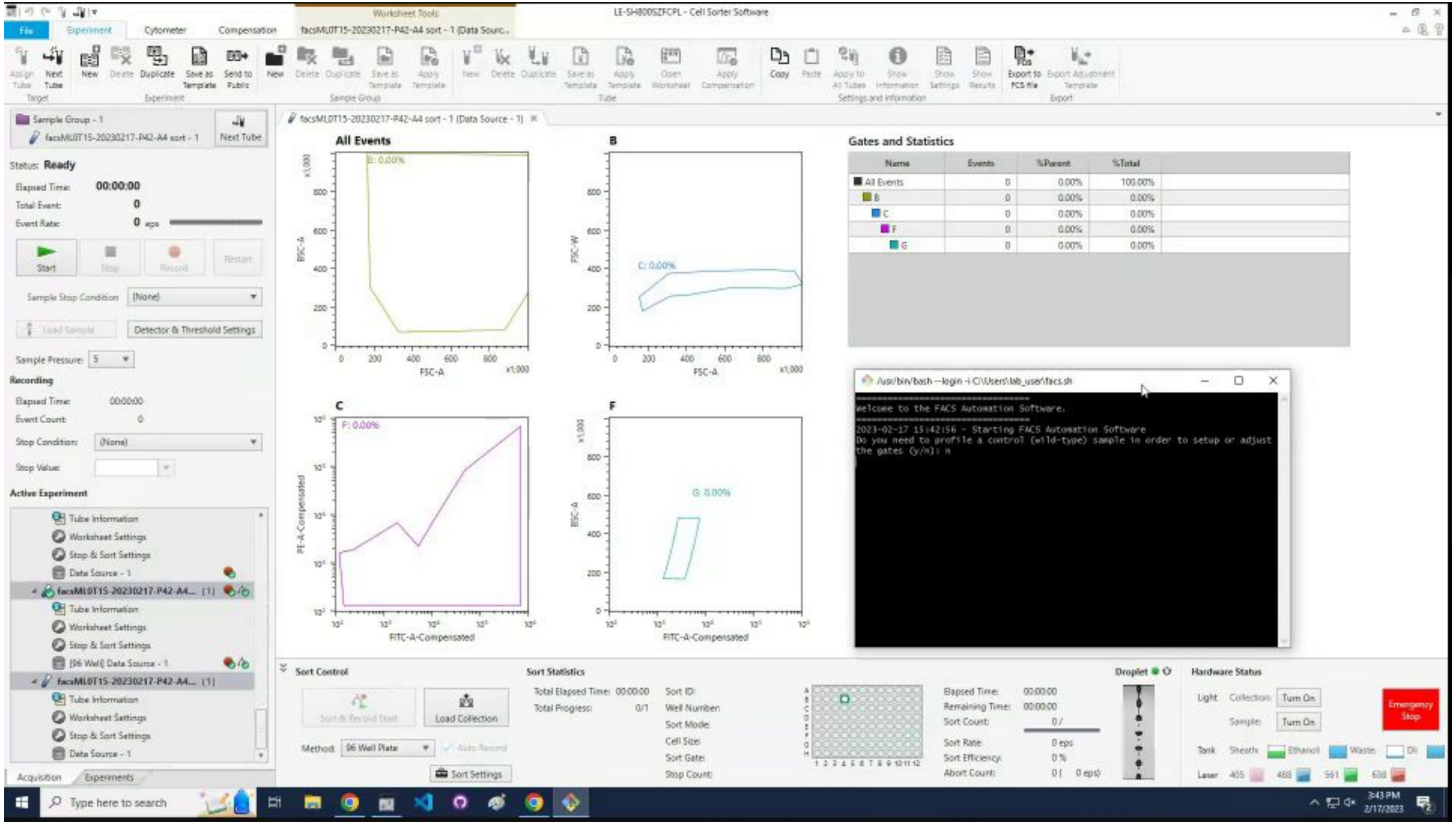

Enter `user name.`

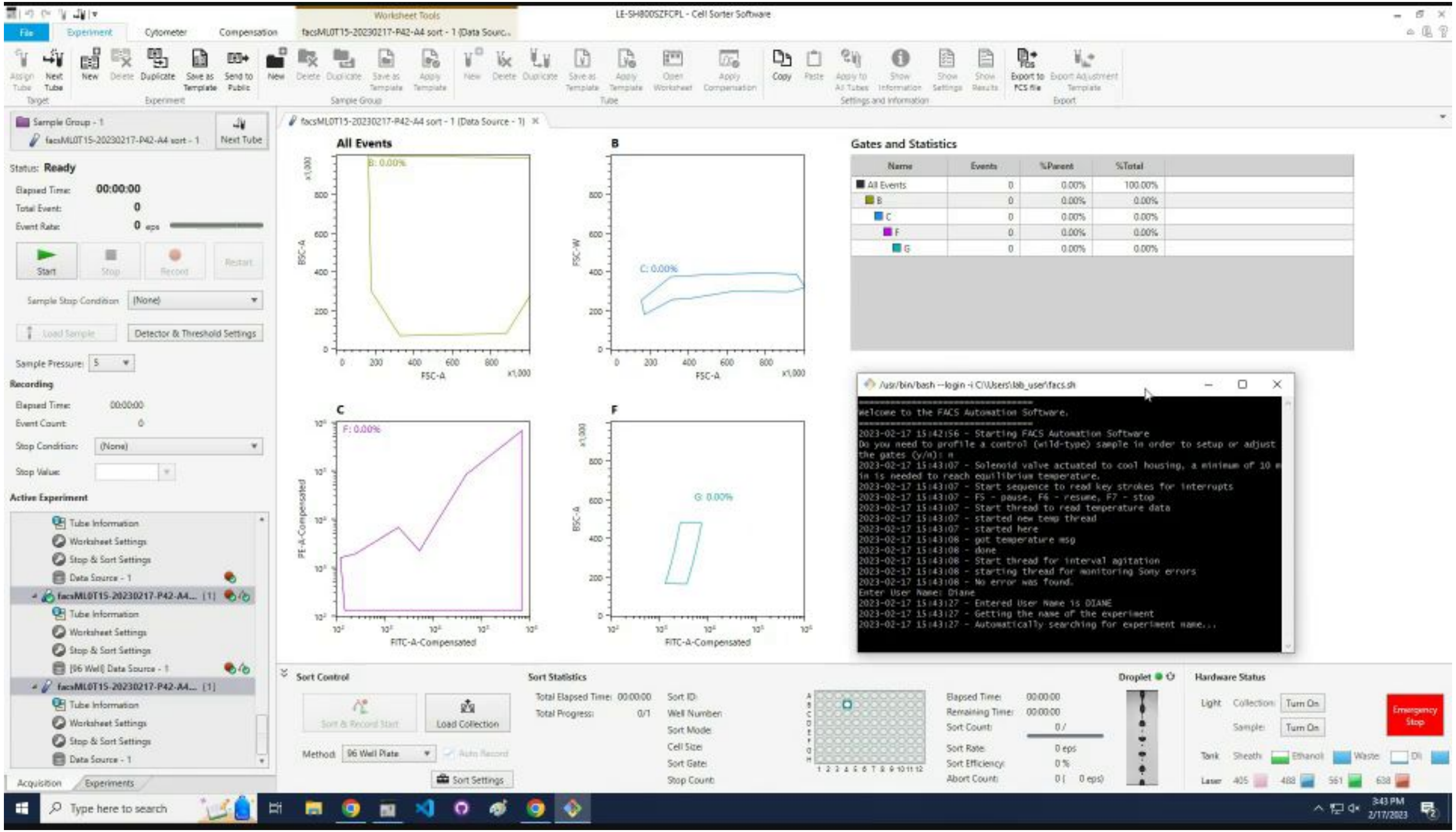

Find experiment name to enter into program.

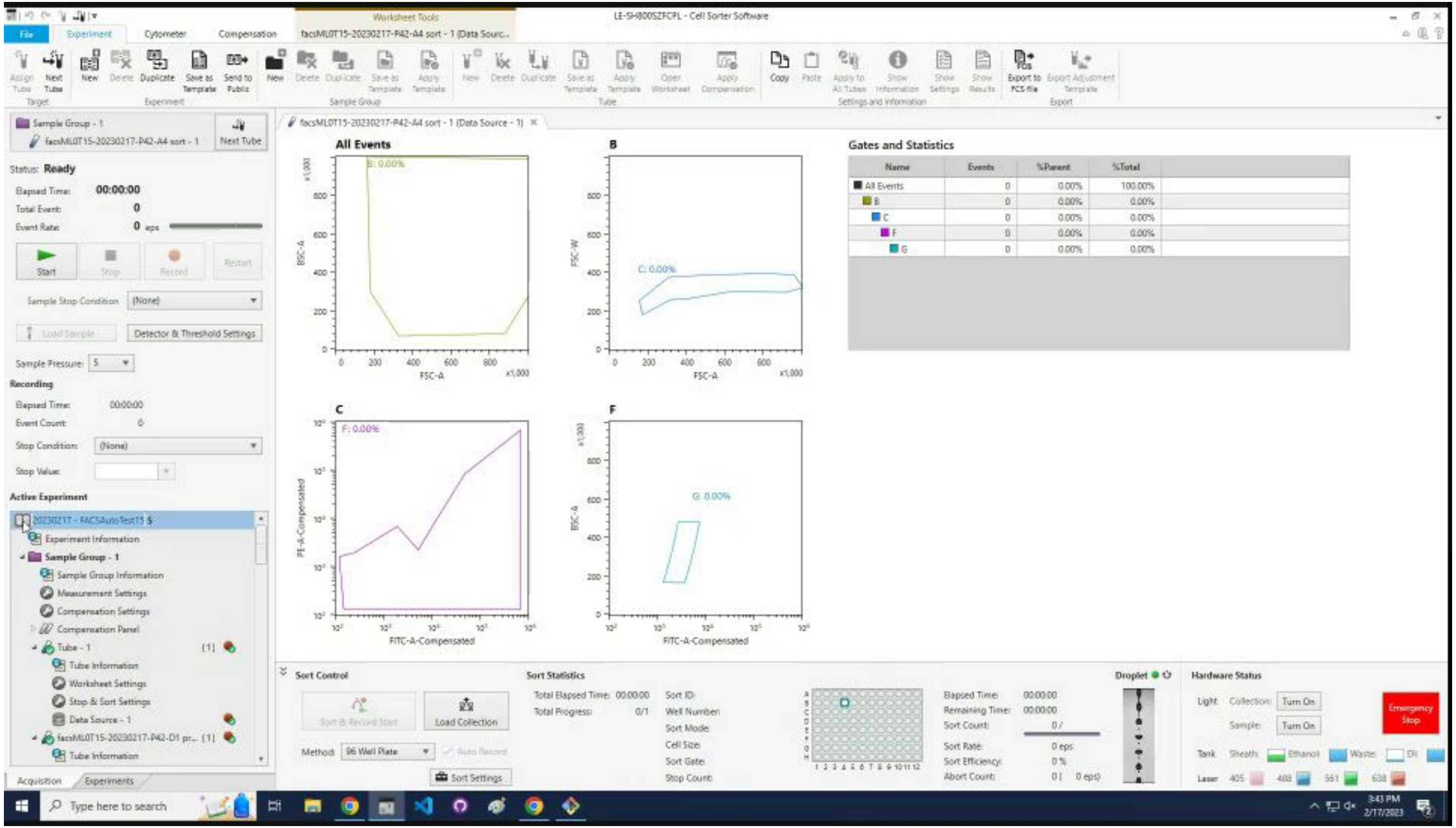

Confirm that the experiment name is correct.

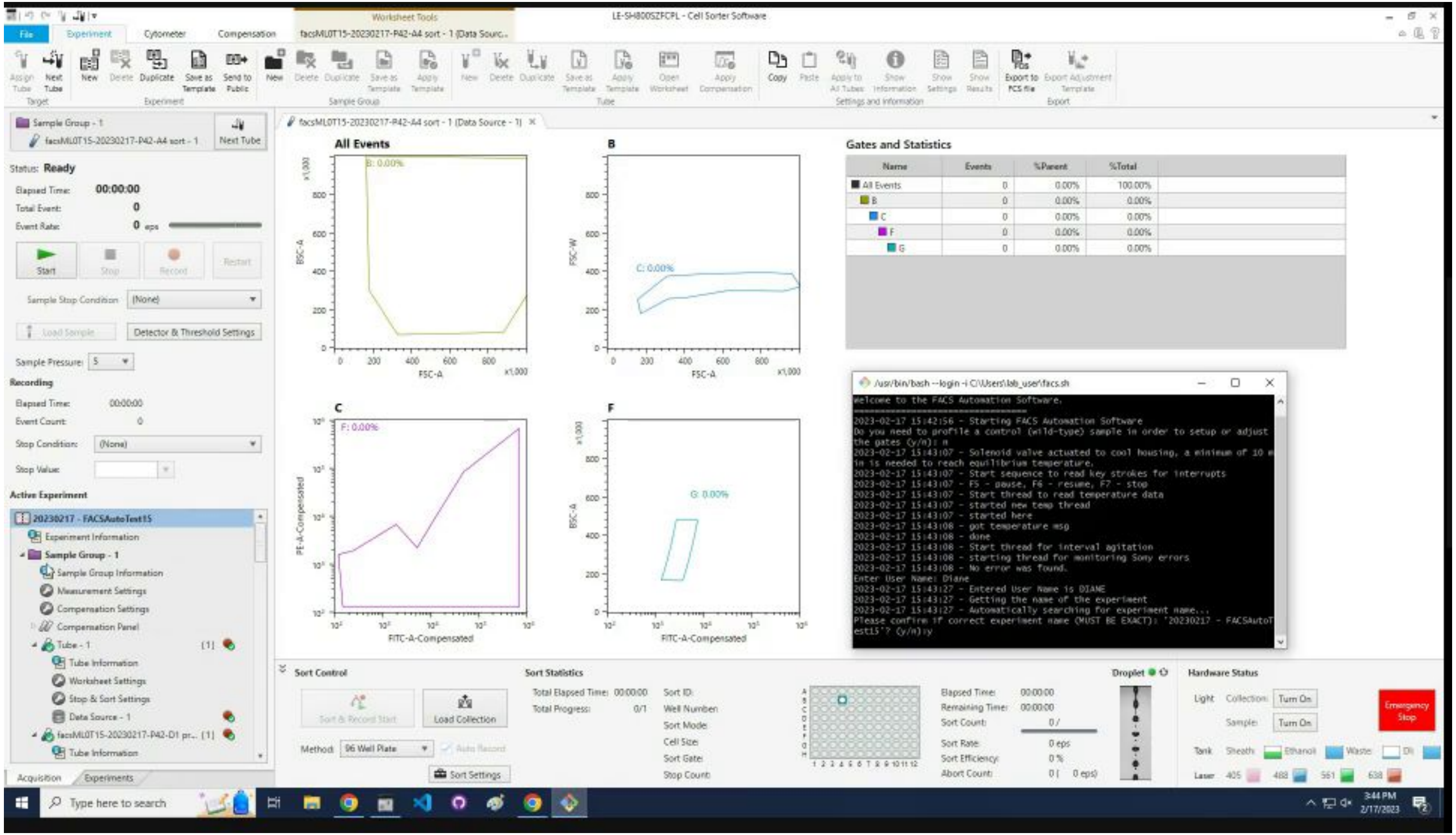

Enter the first tube number to start the sorting process.

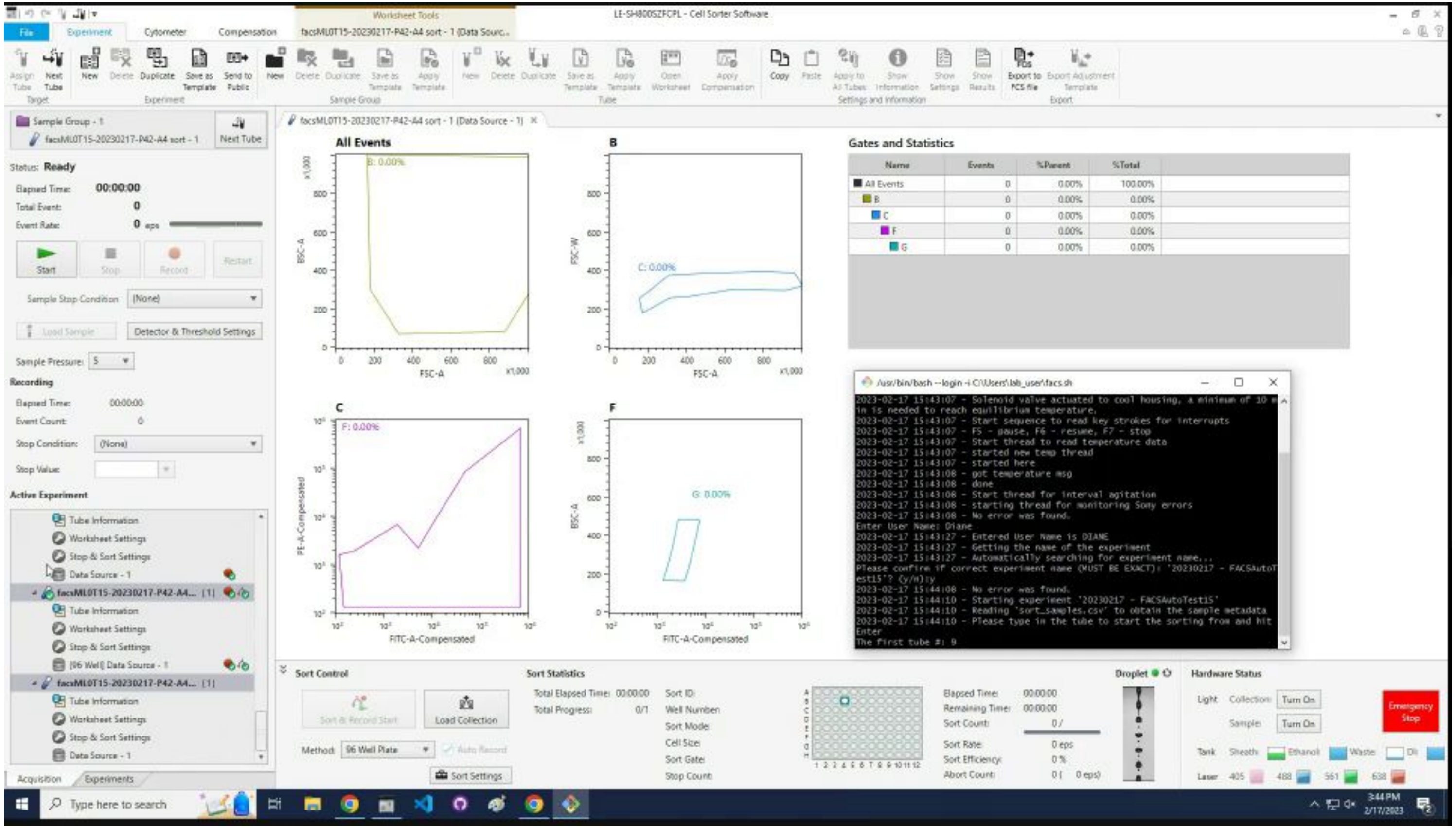

Click `Sort Settings.`

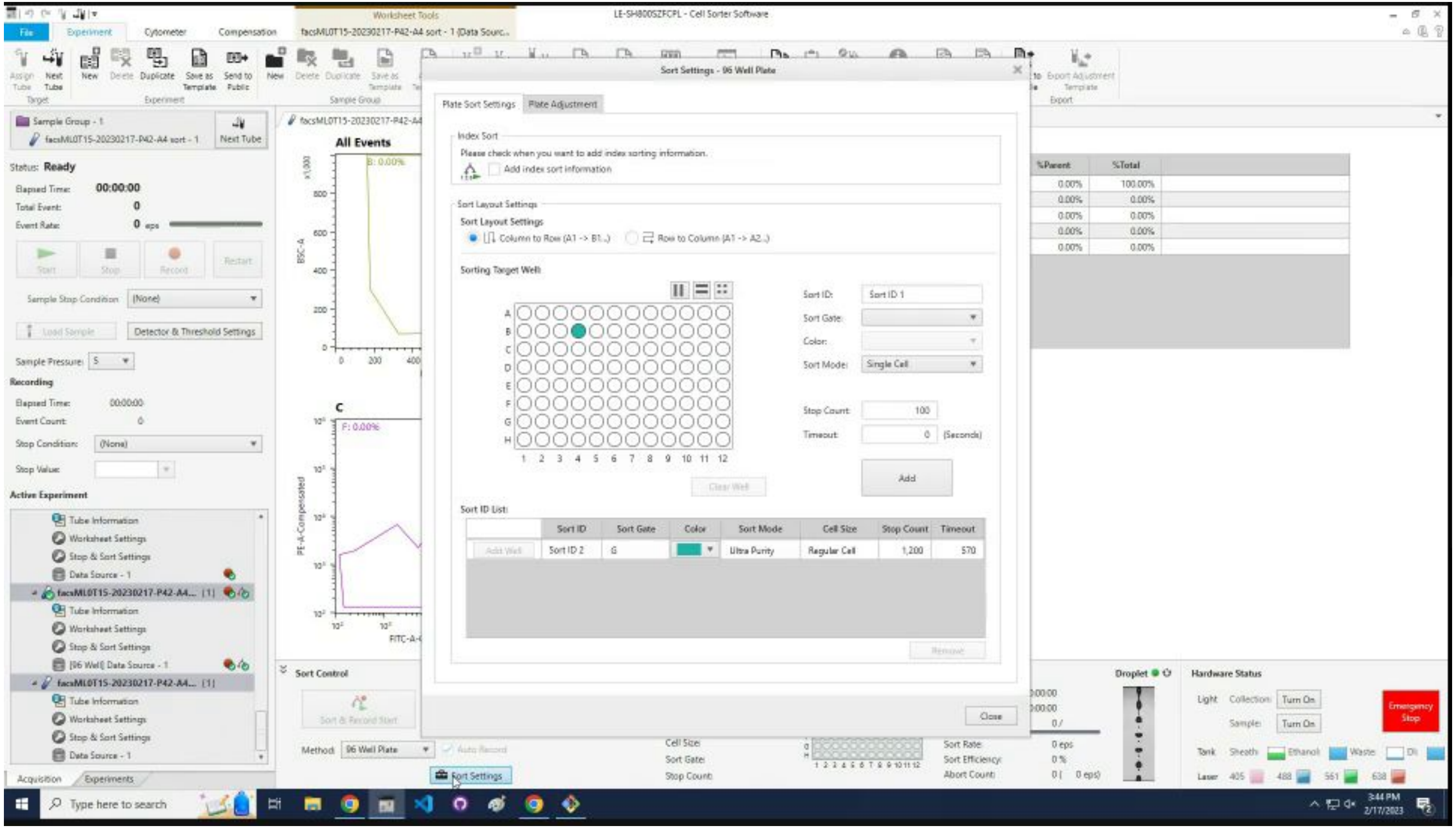

Select the first line and click `Remove`.

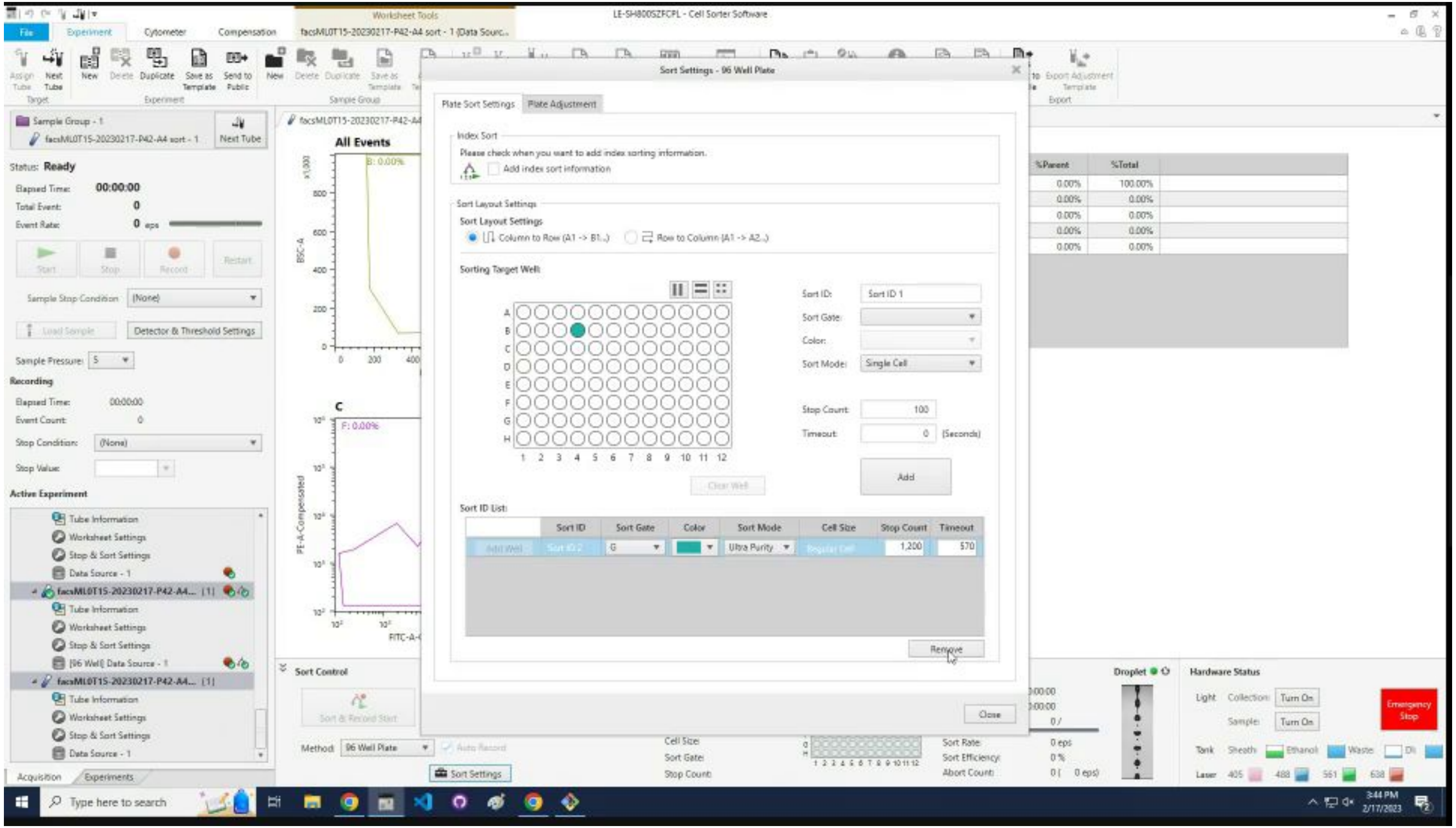

Click `Close`.

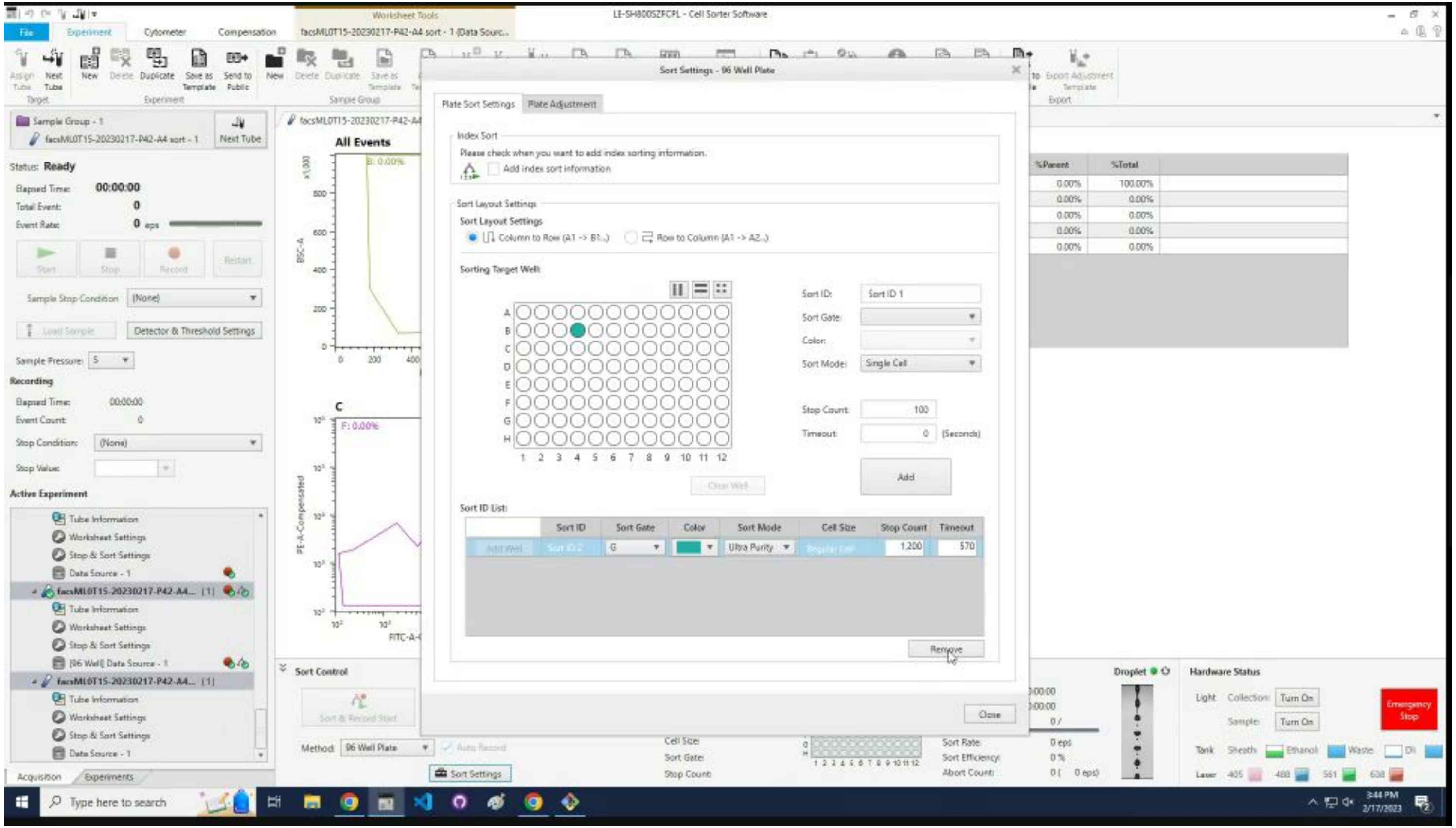

LE-SH800SZFCPL - Cell Sorter Software
Sort Settings - 96 Well Plate
Plate Sort Settings
Plate Adjustment
Index Sort
Please check when you want to add index sorting information.
Add index sort information
Sort Layout Settings
Column to Row (A1 -> B1..)
Row to Column (A1 -> A2..)
Sorting Target Well
Sort ID:
Sort Gate:
Color:
Sort Mode:
Stop Count:
Timeout:
Add
Clear Well
Sort ID List
Remove
Close

Click on the assigned tube in the list.

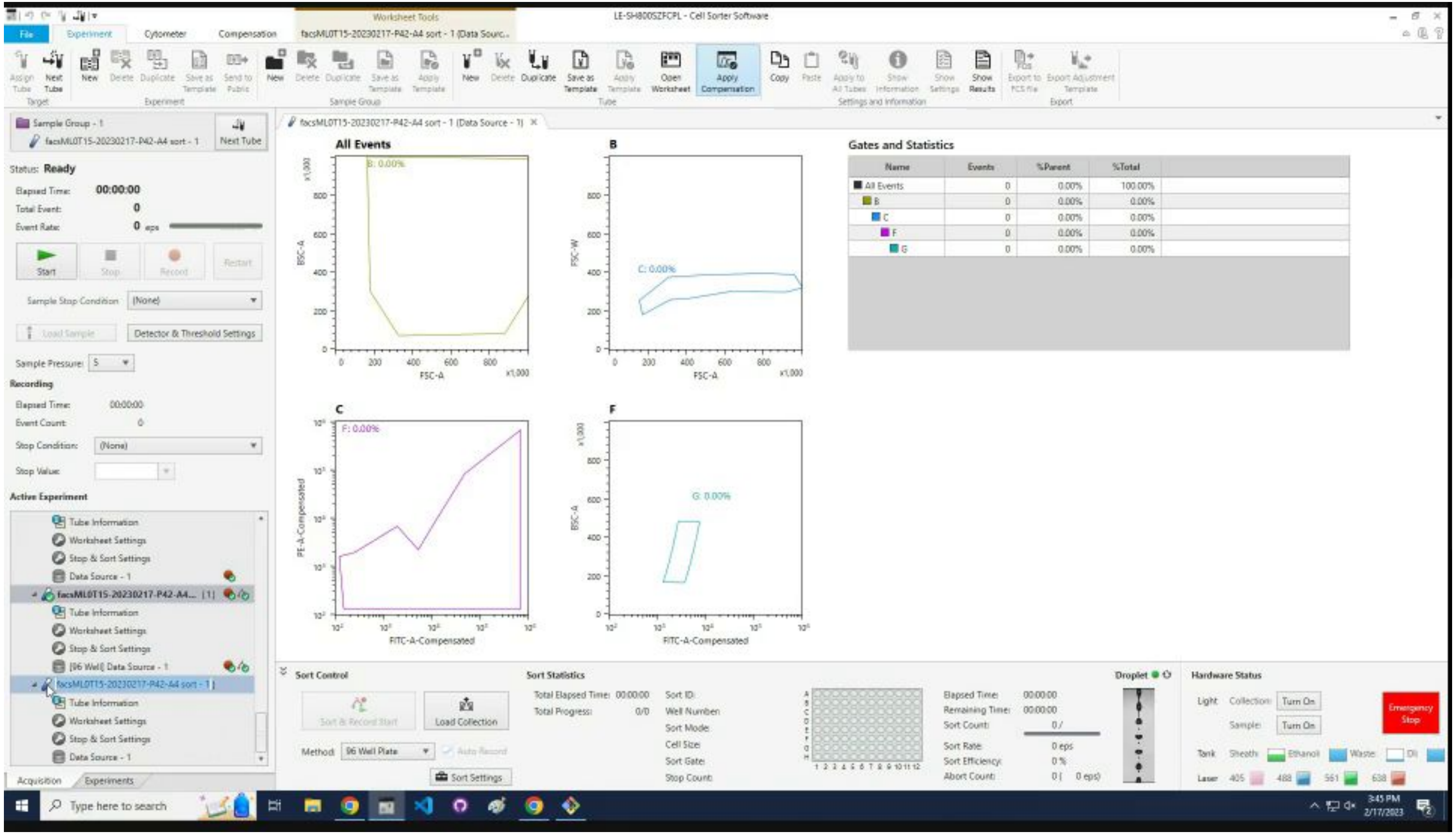

Goto `Iterate through samples` section.

Iterate through samples

Press `F2` to rename the active tube.

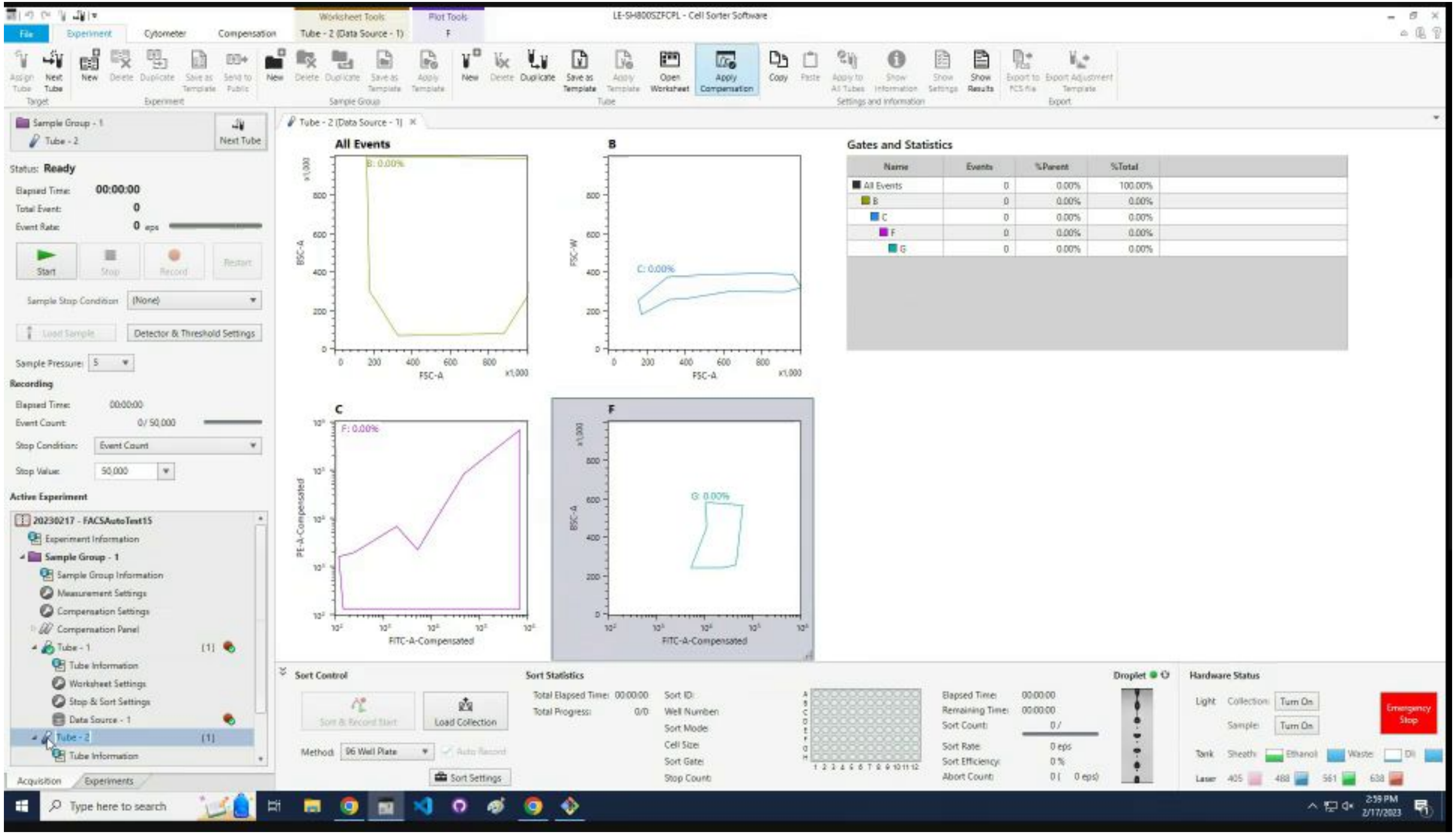

Rename to the sample name appended with profile.

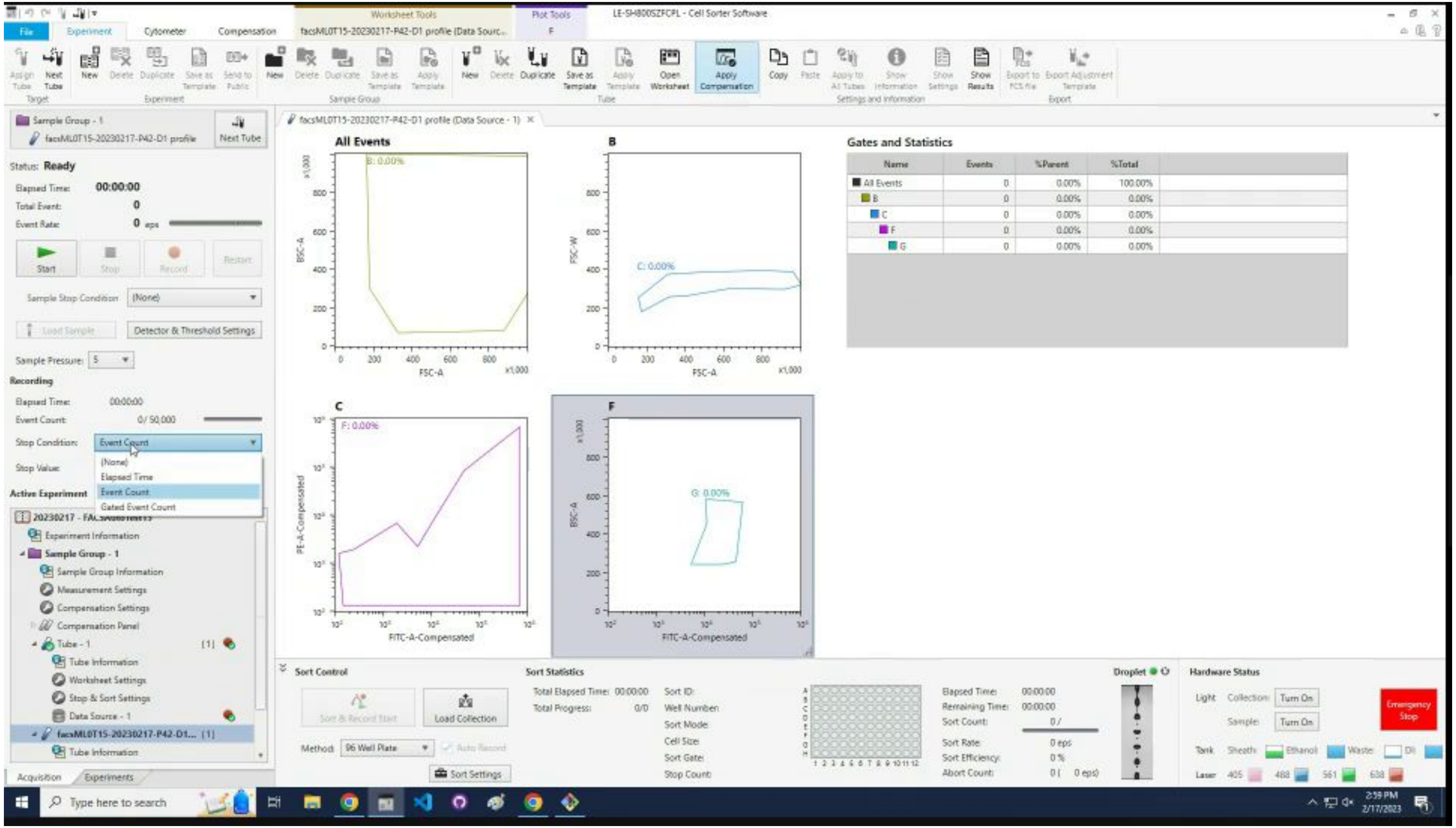

Change the `Stop Condition` to `Event Count.`

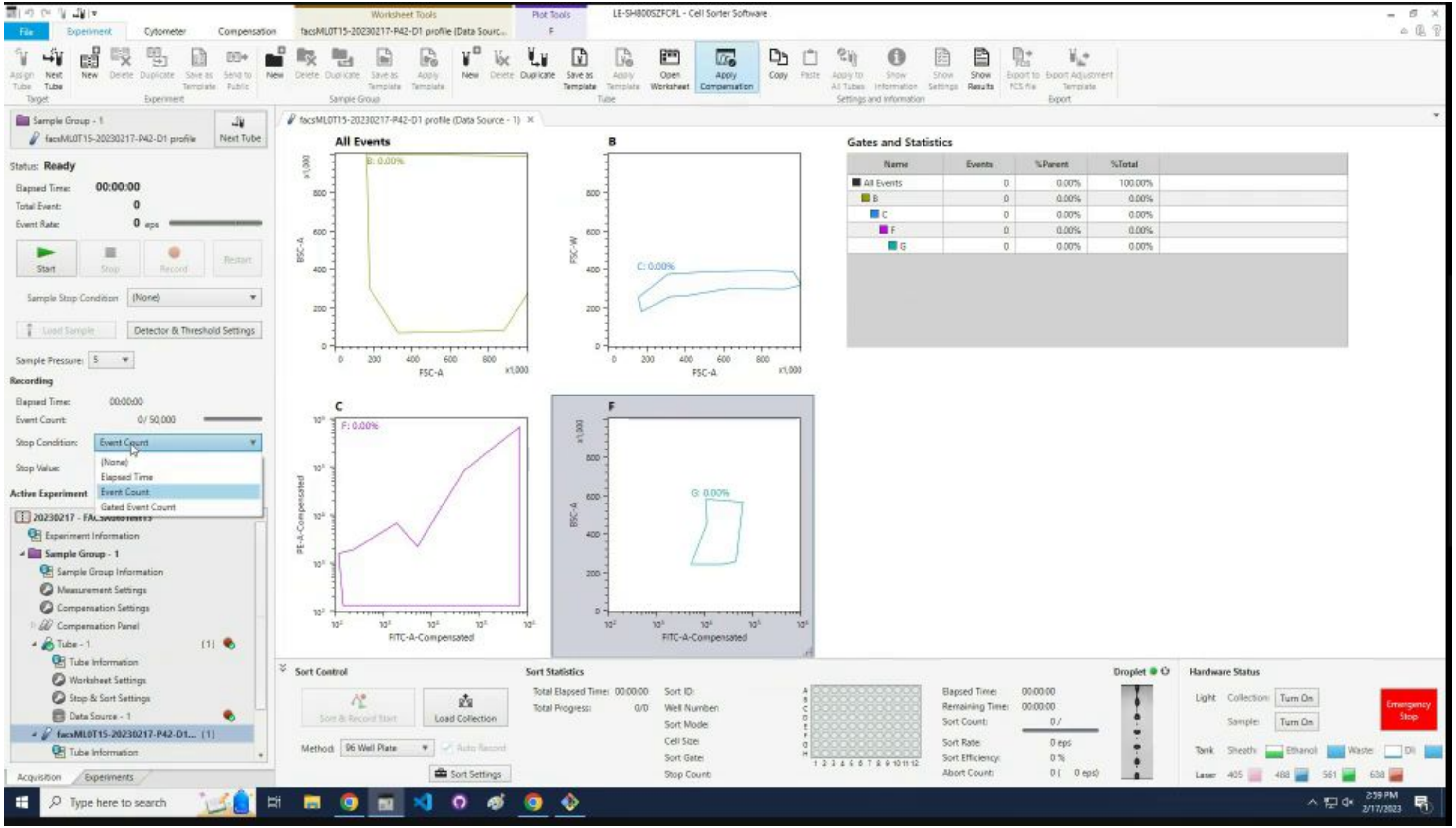

Change the `Sample Pressure` to 5.

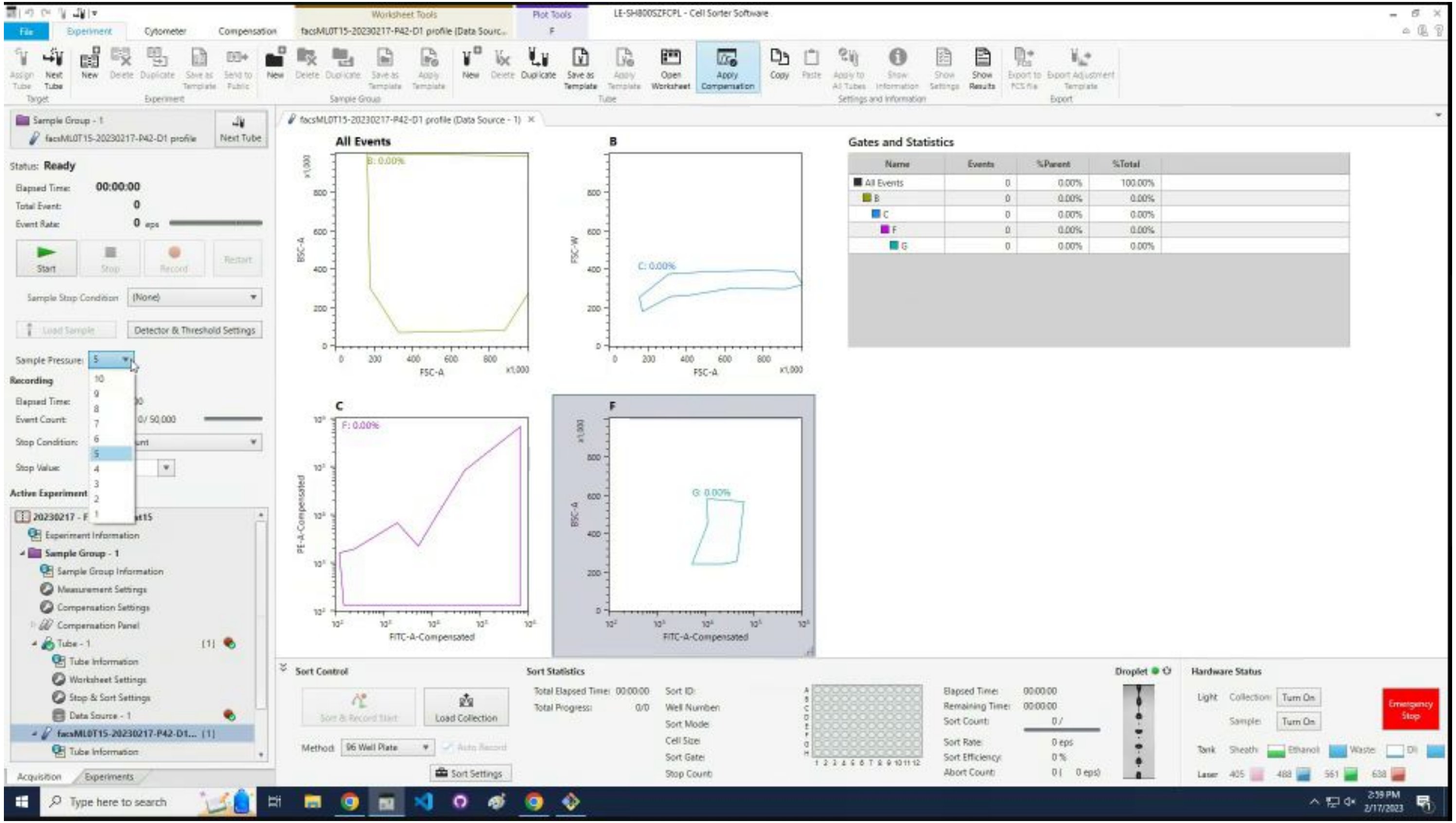

Press Start.

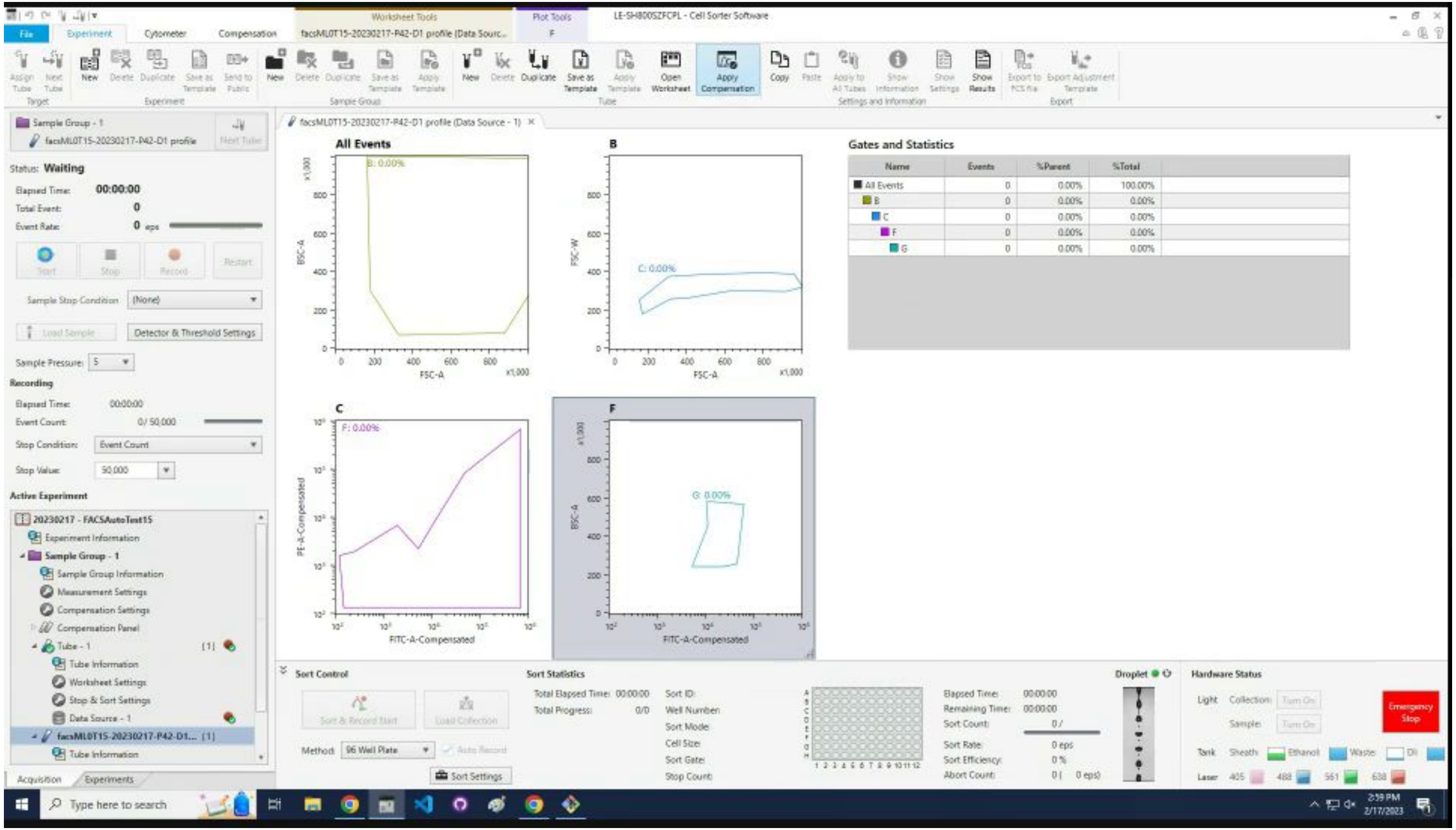

Wait ~20 seconds for cells to appear. Click `Record`.

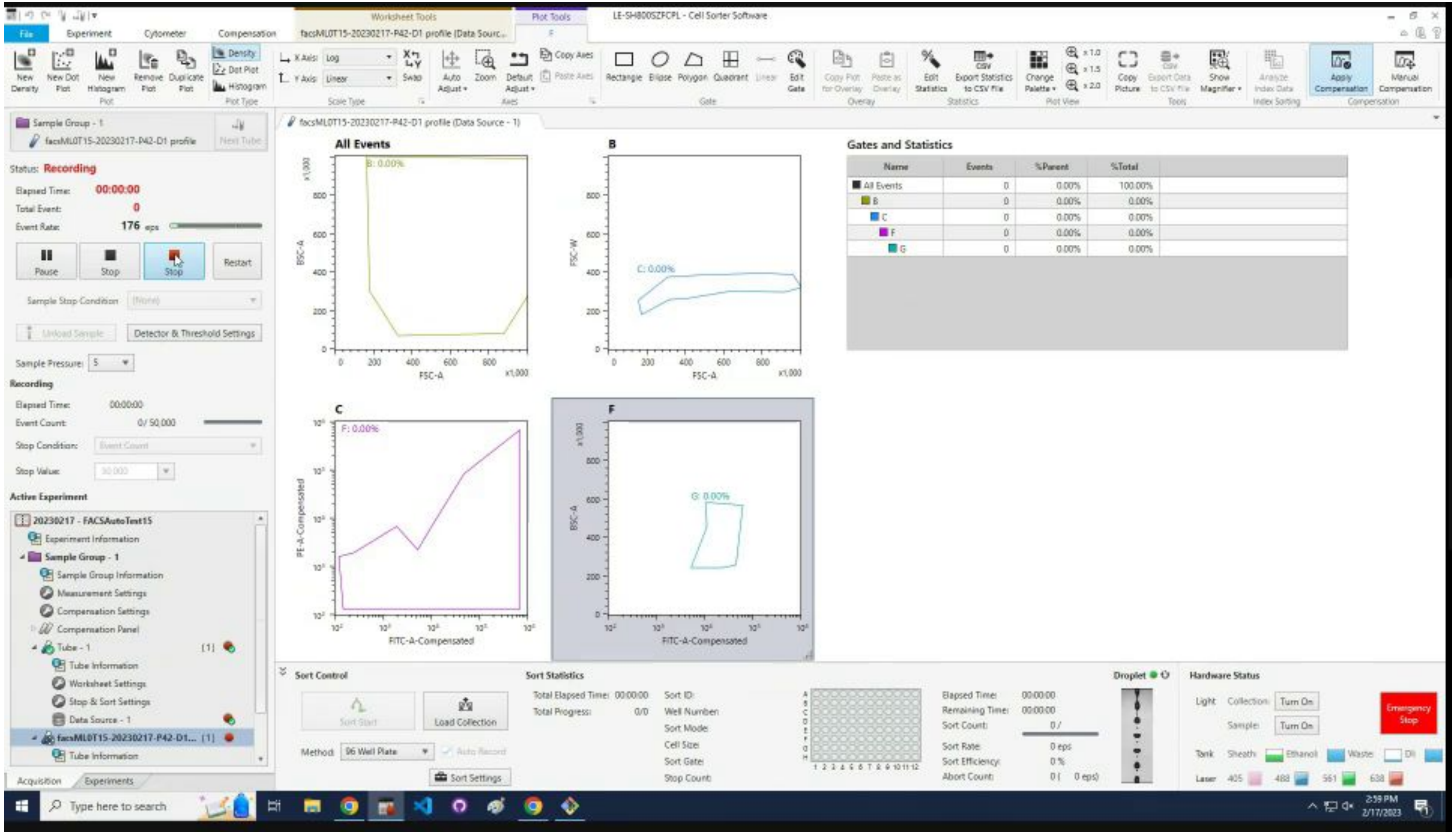

Wait for profiling to finish then click `OK`.

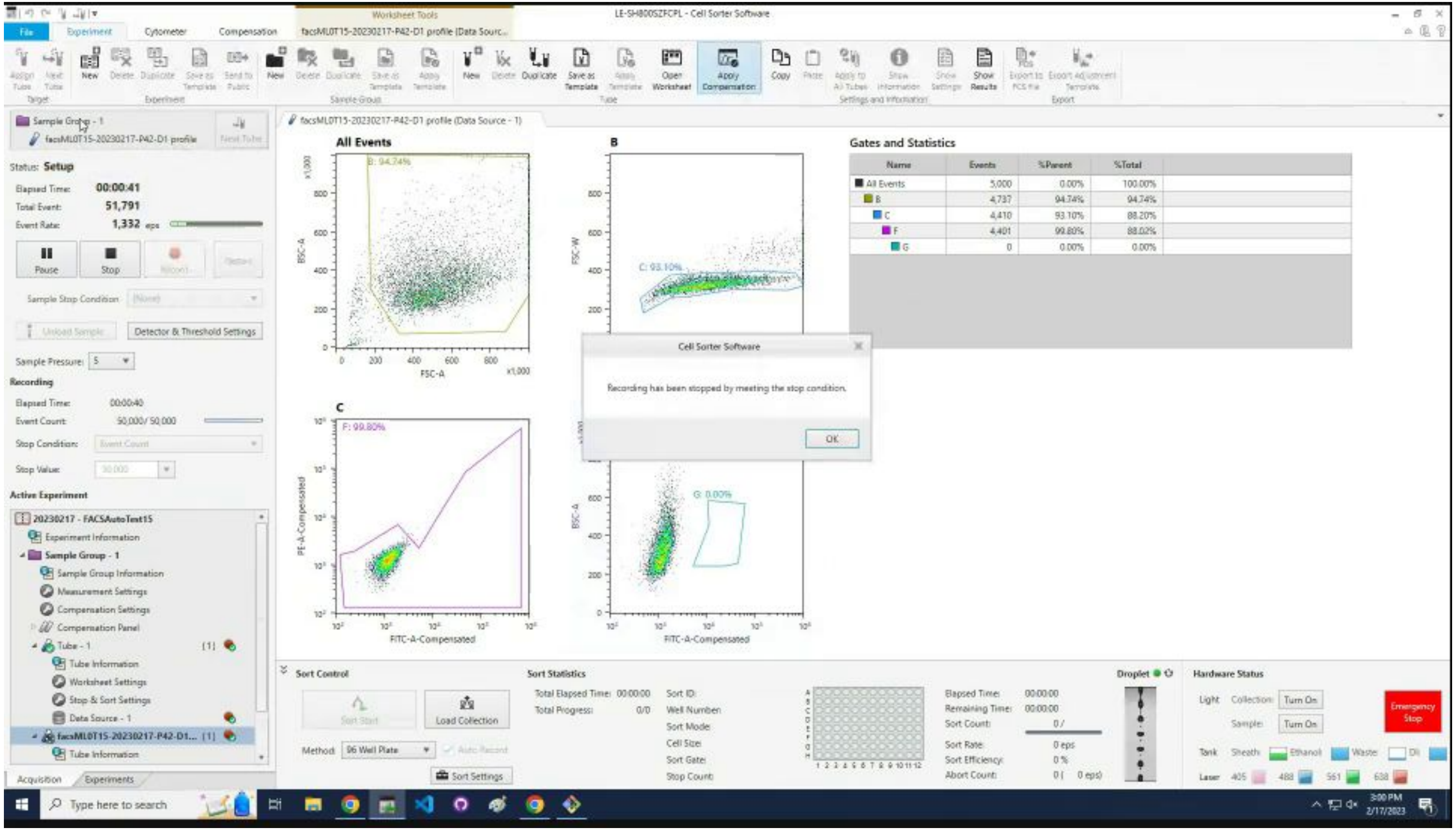

Click `Stop`.

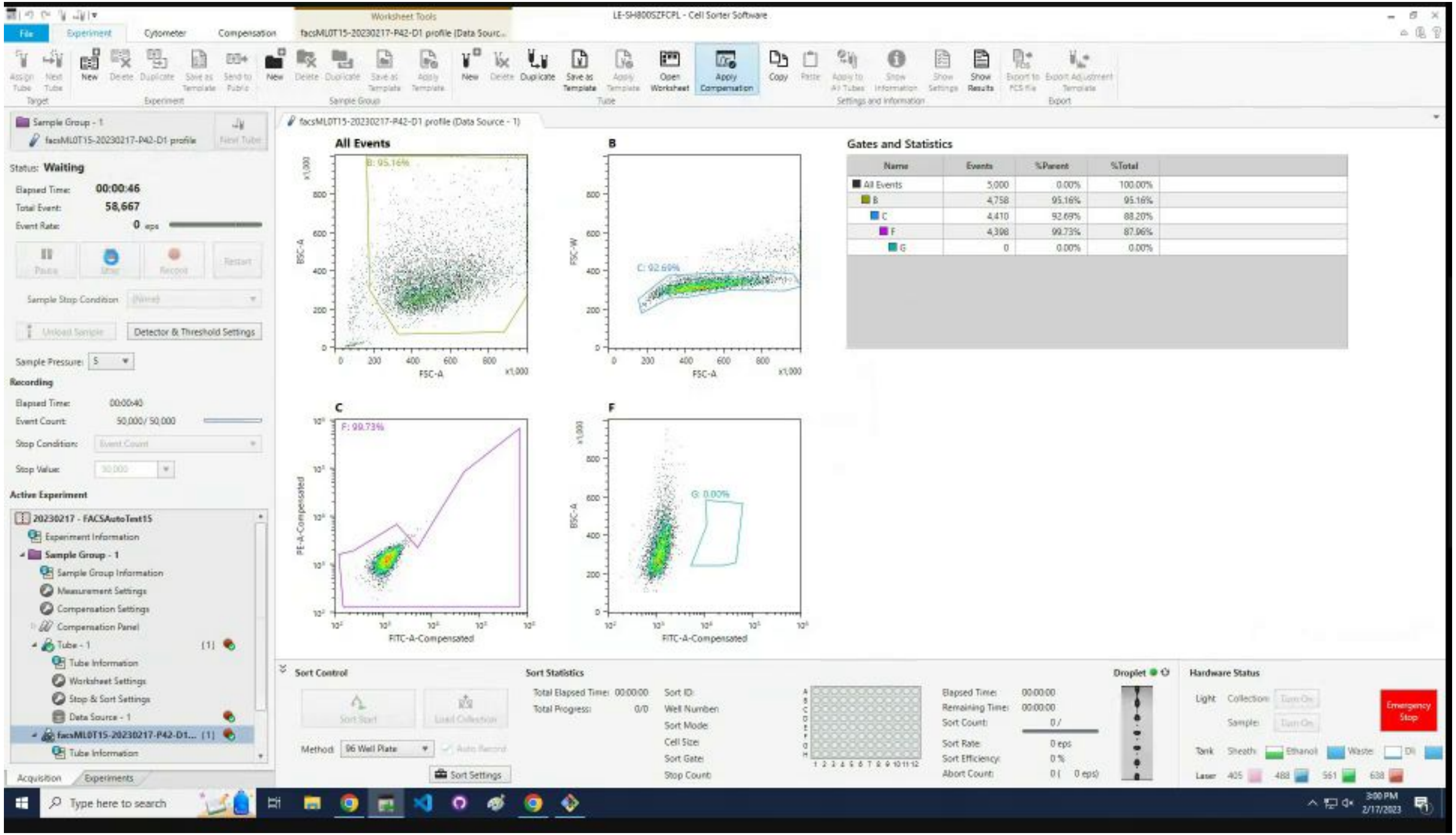
LE-SH800SZFCPL - Cell Sorter Software
File
Experiment
Cytometer
Compensation
Worksheet Tools
Apply Compensation
Open Worksheet
Show Results
Sample Group - 1
Status: Waiting
Elapsed Time: 00:00:46
Total Event: 58,667
Event Rate: 0 eps
Detector & Threshold Settings
Sample Pressure: 5
Recording
Event Count: 50,000 / 50,000
Active Experiment
20230217 - FACSAutoTest15
Experiment Information
Sample Group - 1
Sample Group Information
Measurement Settings
Compensation Settings
Compensation Panel
Tube - 1
Tube Information
Worksheet Settings
Stop & Sort Settings
Data Source - 1
Acquisition
Experiments
All Events
B
C
F
FSC-A
FSC-H
FSC-W
BSC-A
FITC-A-Compensated
PE-A-Compensated
B: 95.16%
C: 92.69%
F: 99.73%
G: 0.00%
Gates and Statistics
Name
Events
%Parent
%Total
All Events
5,000
0.00%
100.00%
4,758
95.16%
4,410
92.69%
88.20%
4,398
99.73%
87.96%
G
0
0.00%
0.00%
Sort Control
Sort Statistics
Method 96 Well Plate
Sort Settings
Droplet
Hardware Status
Emergency Stop
Type here to search

Click on the fourth plot.

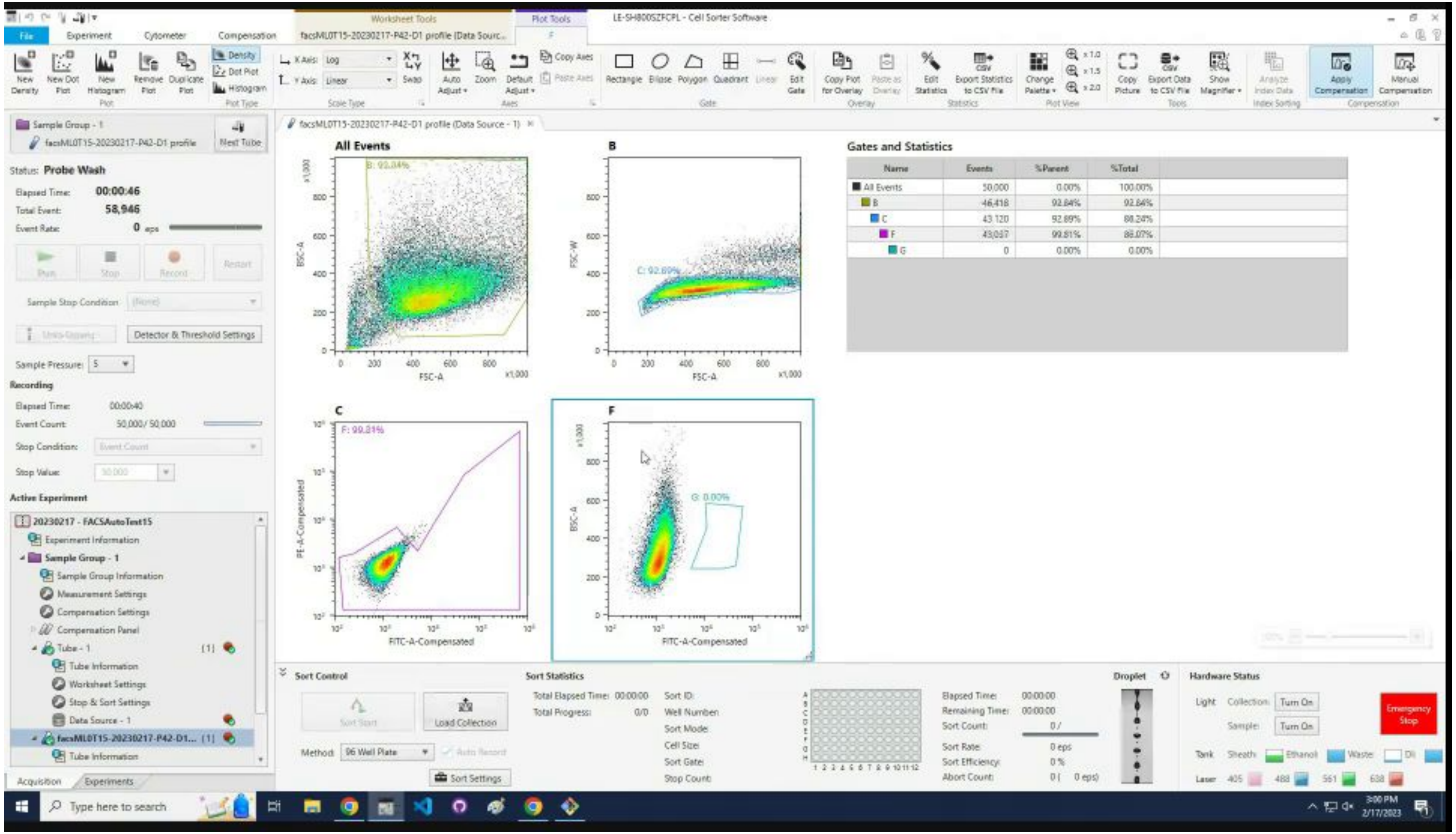

Click `Export Data to CSV File` icon.

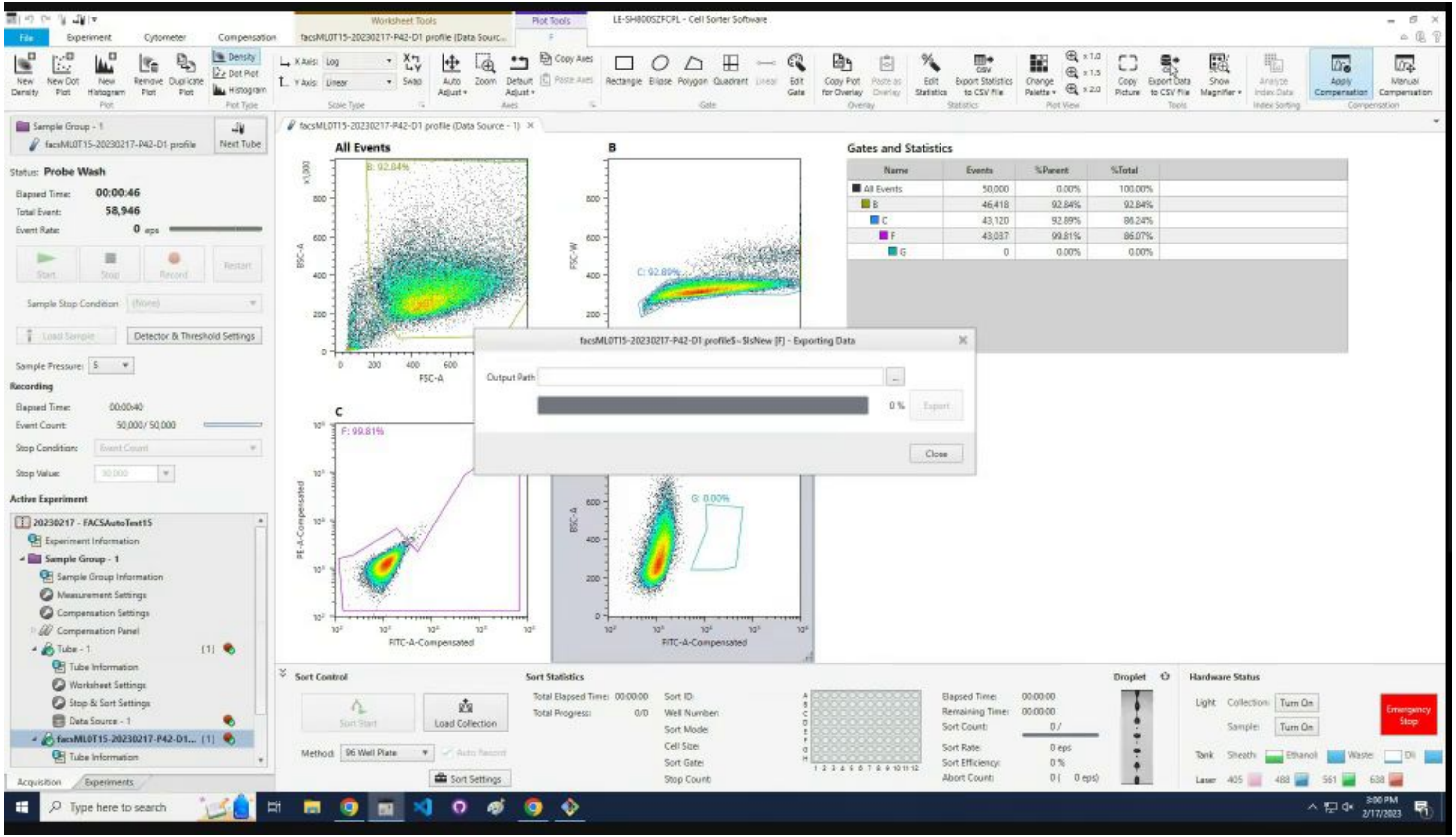

Click the … button in the popup.

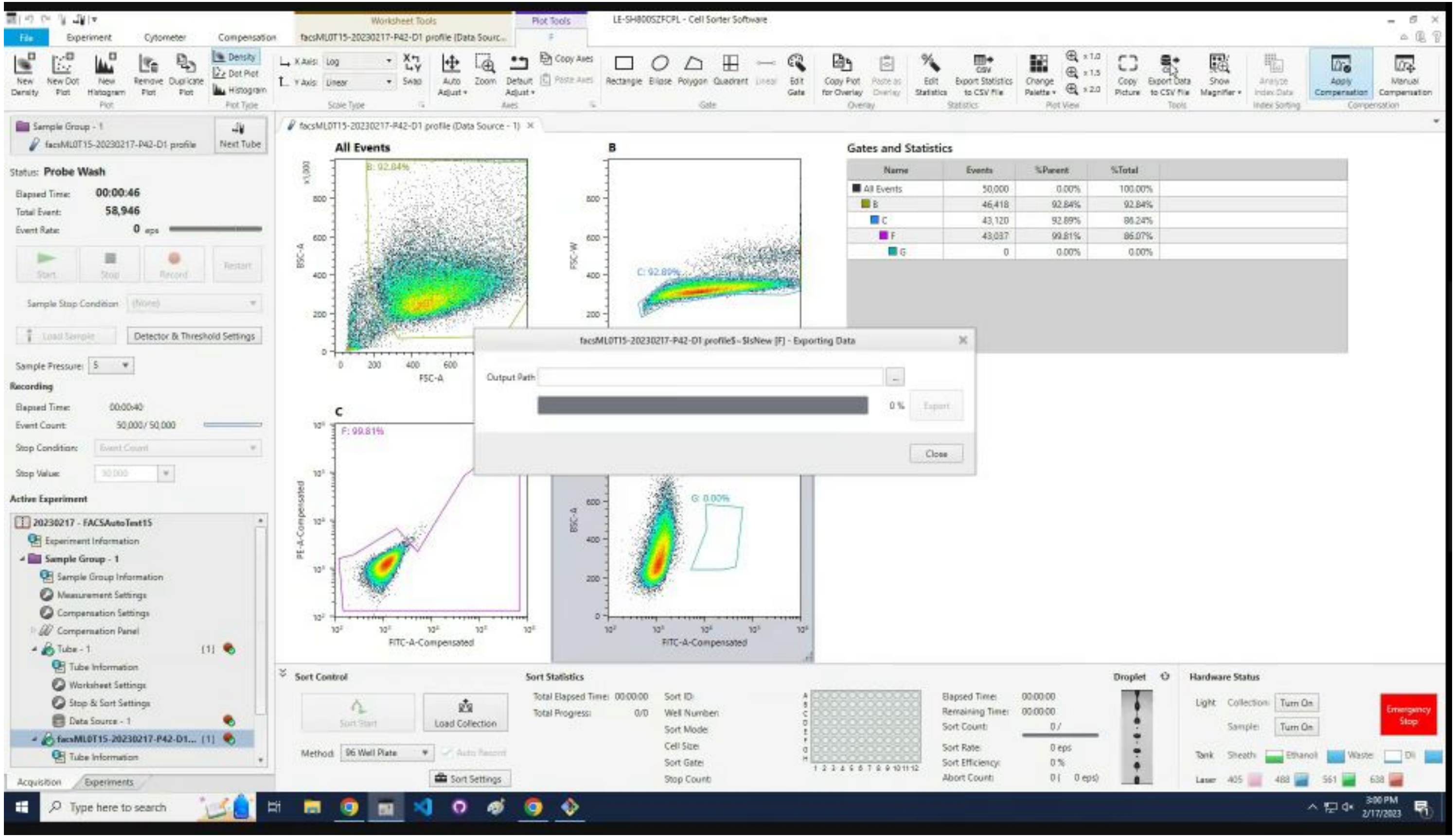

Select all of the file path and file name.

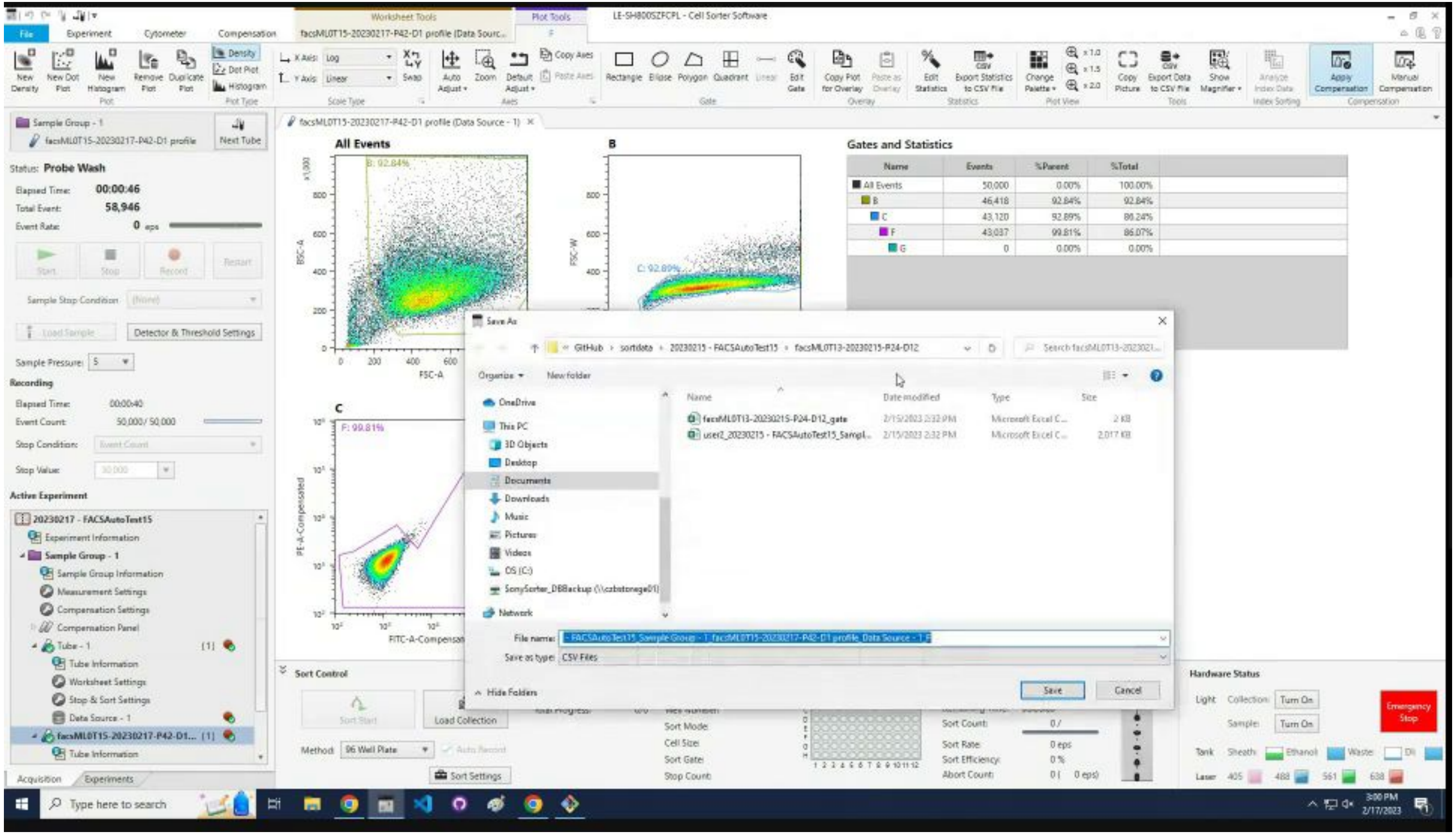

Rename the file as

`C:\Users\lab_user\Desktop\FACSAuto\{User}_{Experiment}_{SampleGroup}_{Sample}`

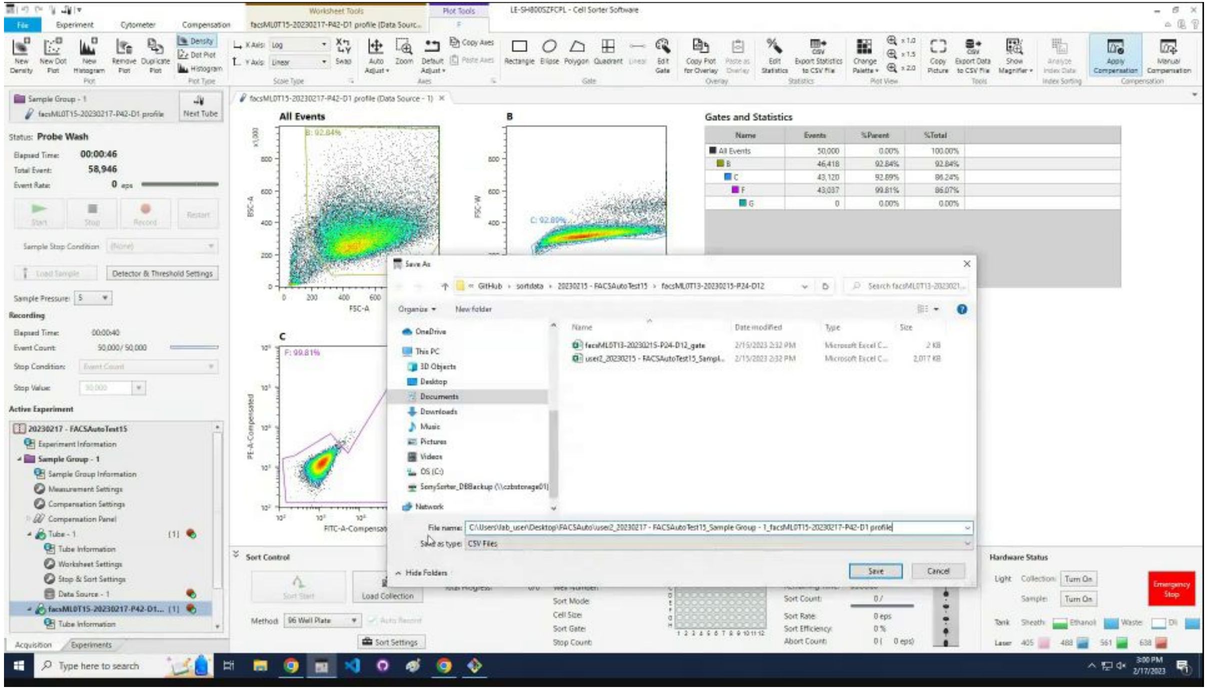

Click Save.

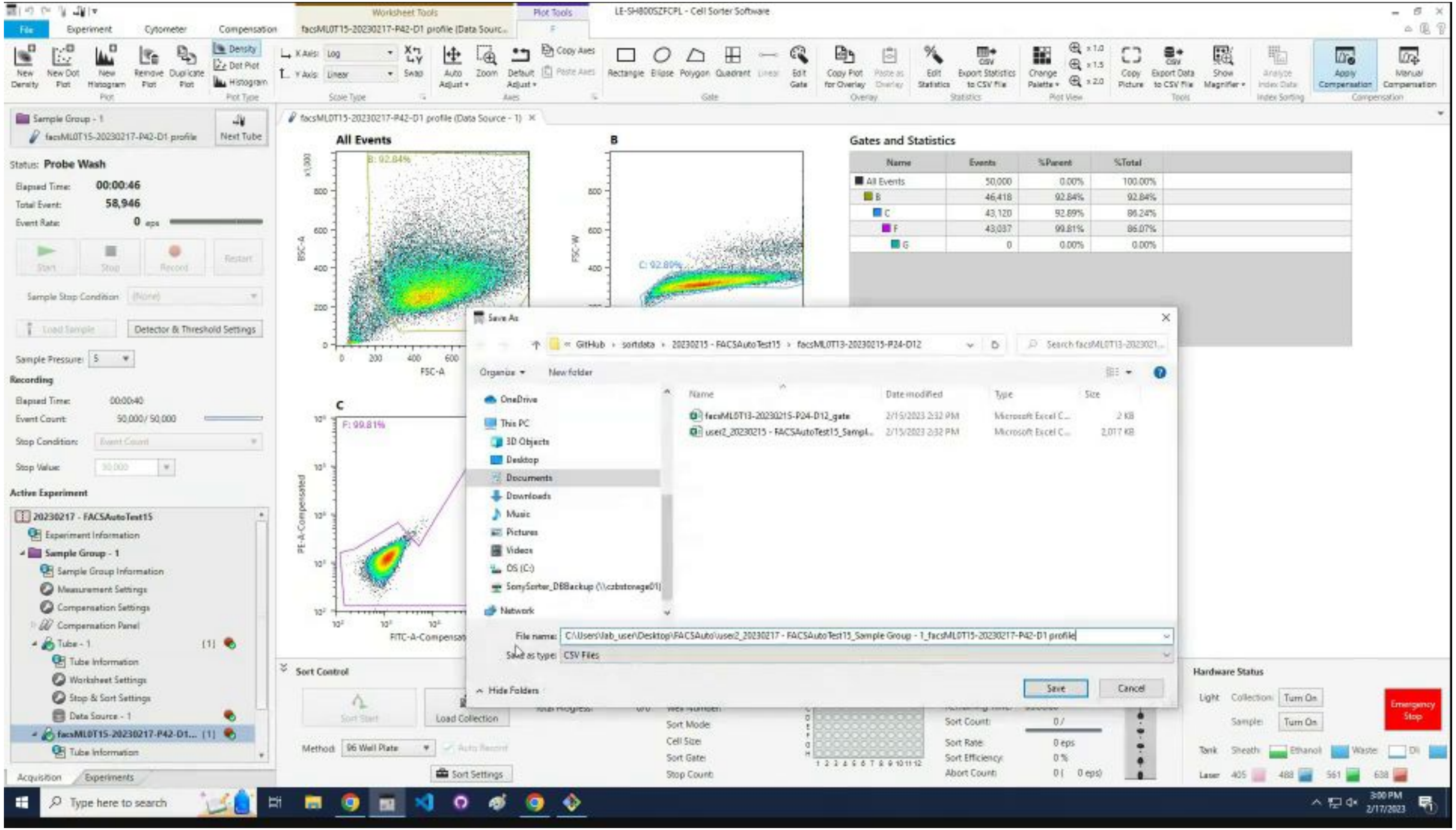

Save As
File name: C:\Users\lab_user\Desktop\FACSAuto\user2_20230217 - FACSAutoTest15_Sample Group - 1_facsMLOT15-20230217-P42-D1 profile
Save as type: CSV Files
Save
Cancel
Gates and Statistics
All Events

Click `Export`.

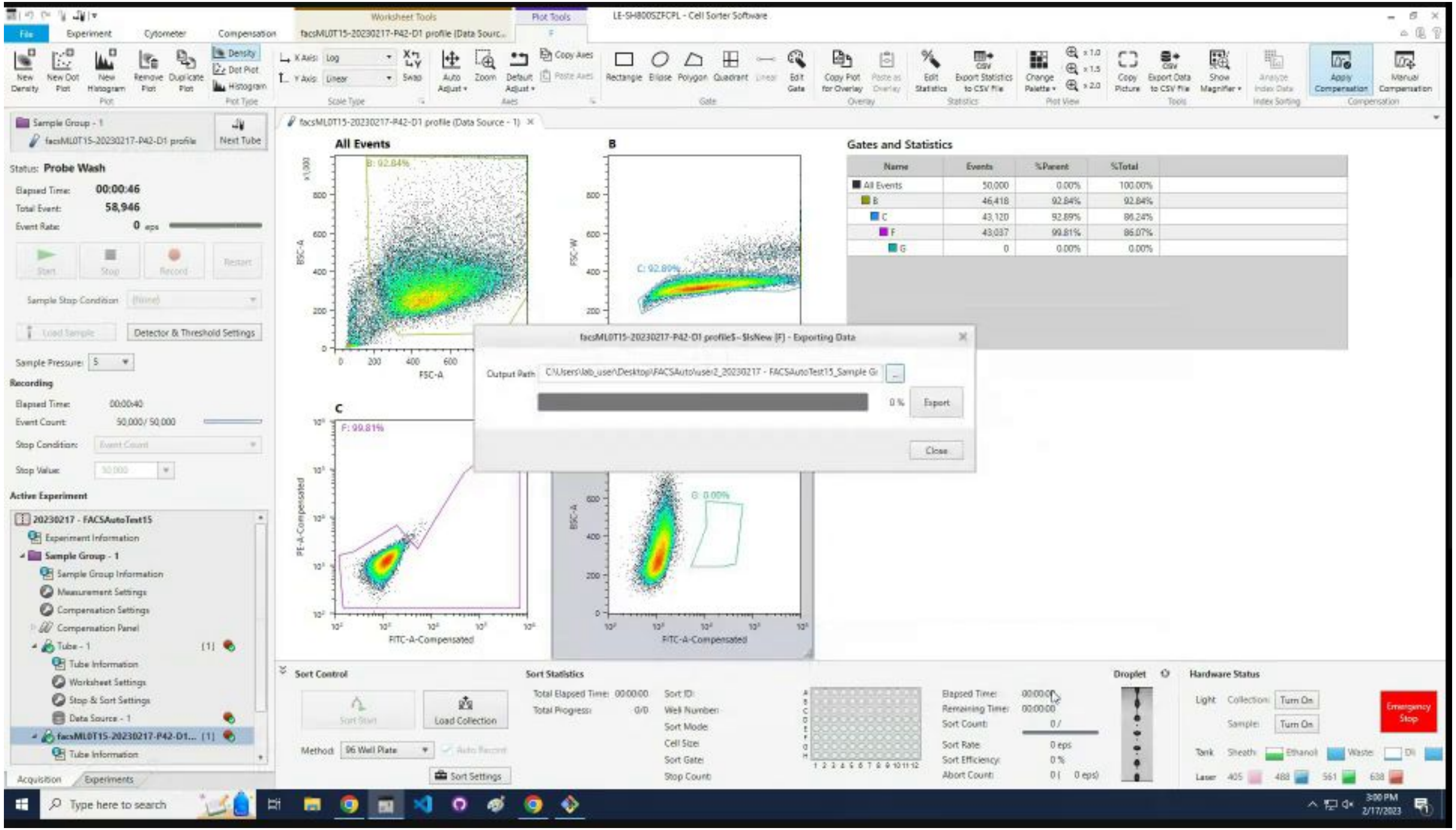

Wait for the export to finish.

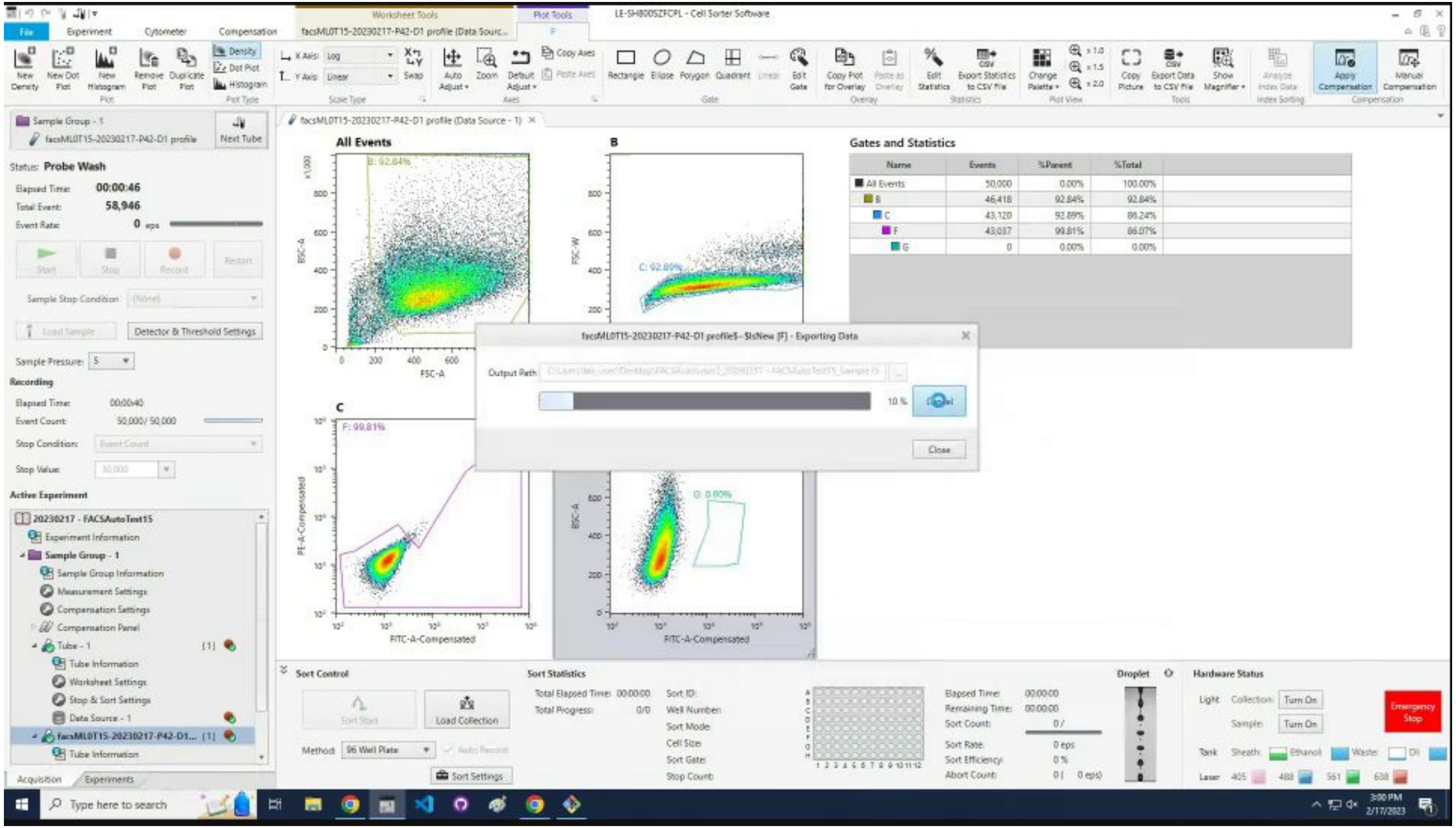

Click OK.

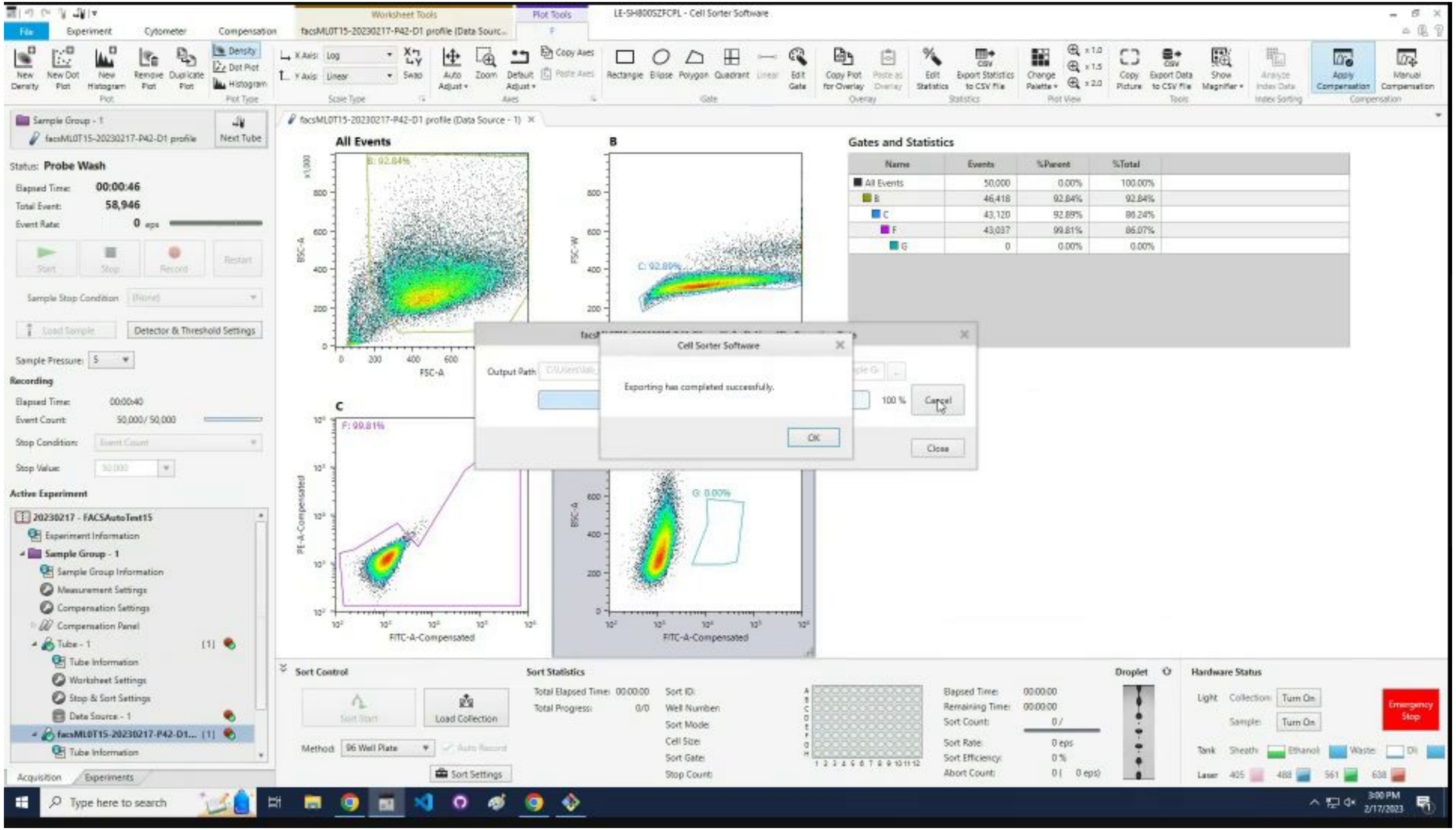

LE-SH800SZFCPL - Cell Sorter Software
Cell Sorter Software
Exporting has completed successfully.
OK
Cancel
Close
Gates and Statistics
All Events
Sort Control
Sort Statistics
Hardware Status

Click `Close`.

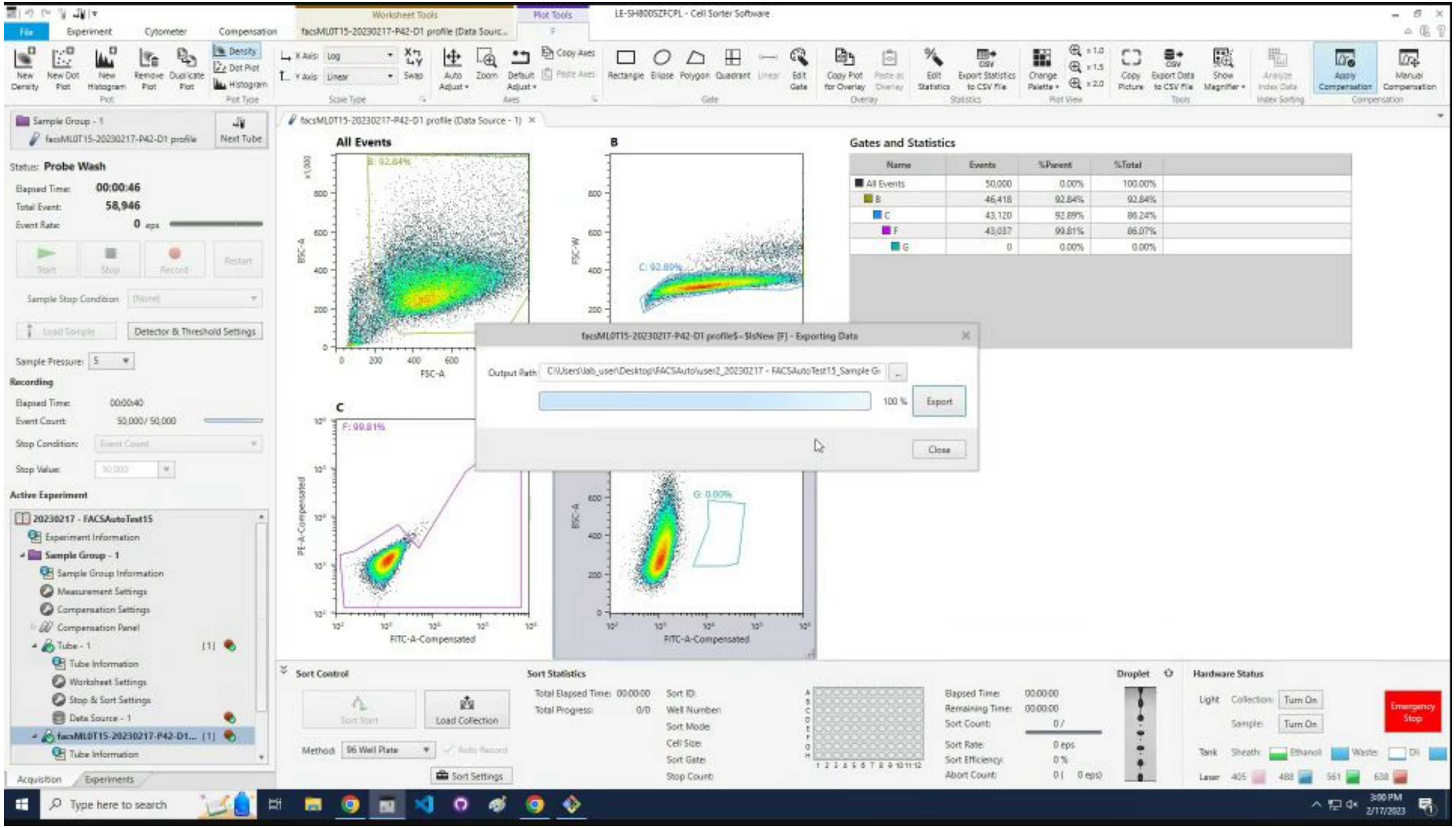

LE-SH800SZFCPL - Cell Sorter Software
Gates and Statistics
Output Path
Export
Close

Click the X to close the tube tab.

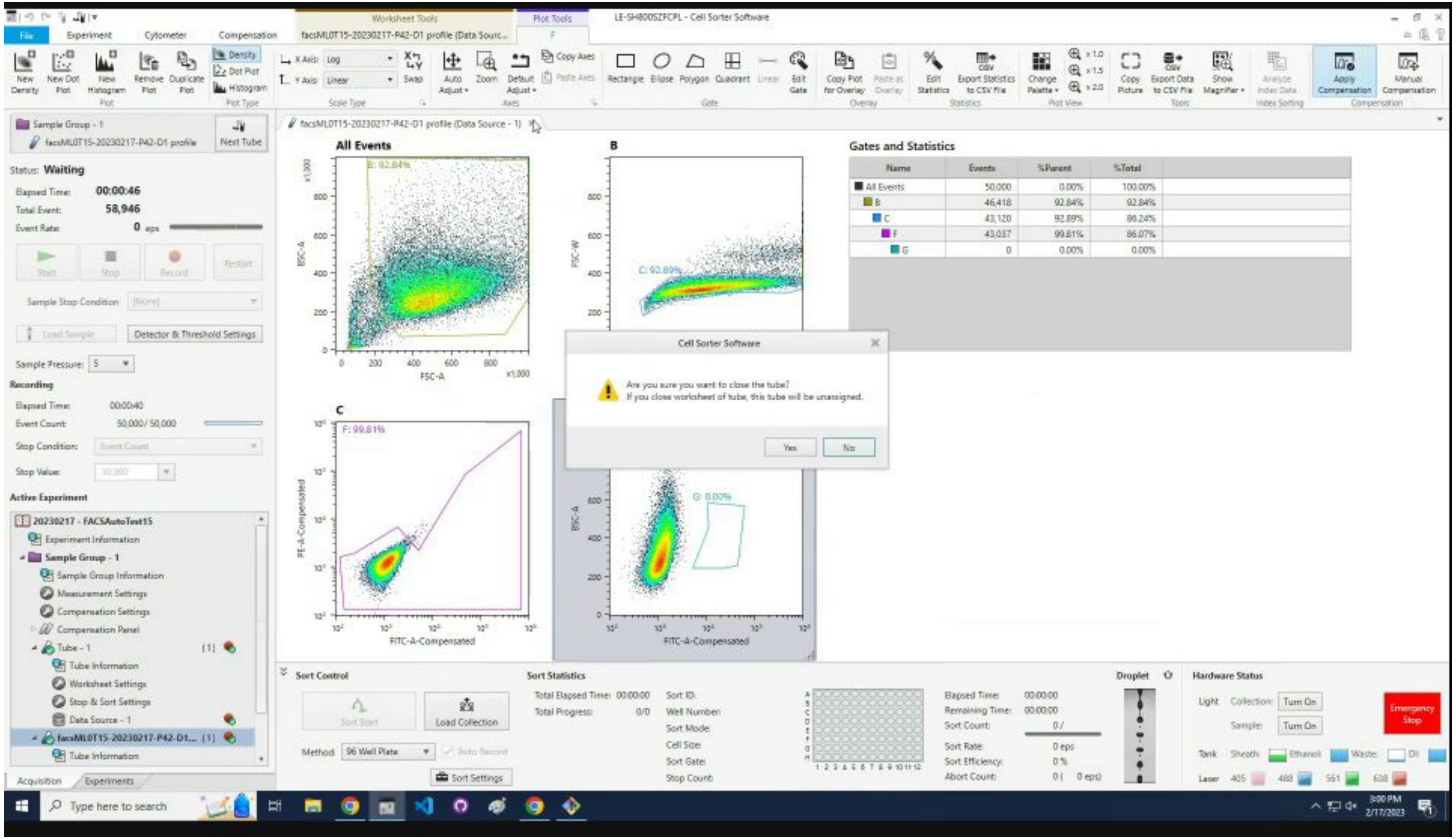

Click `Yes` to confirm closing the tube tab.

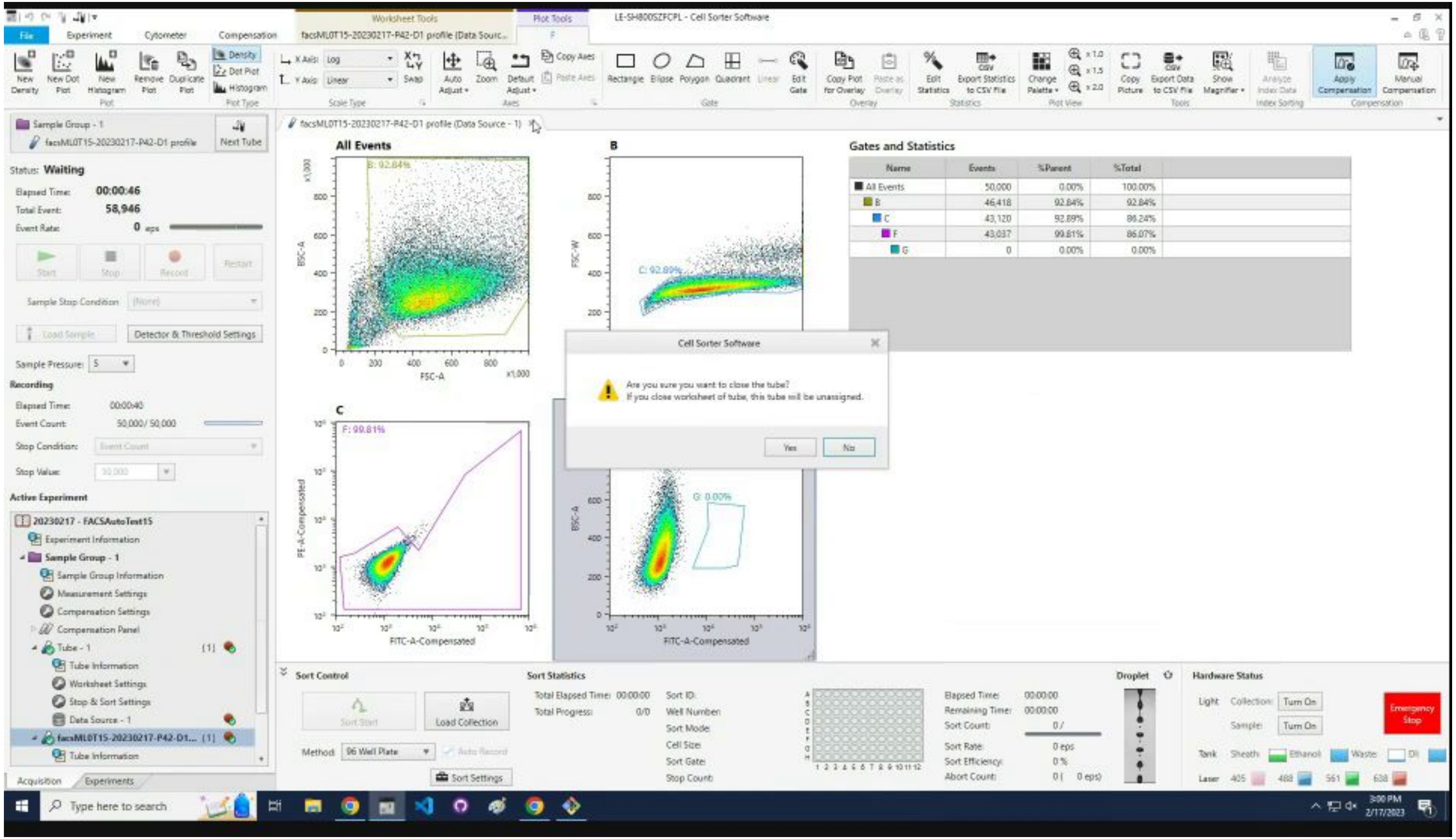

Wait for the automation to create the gate.

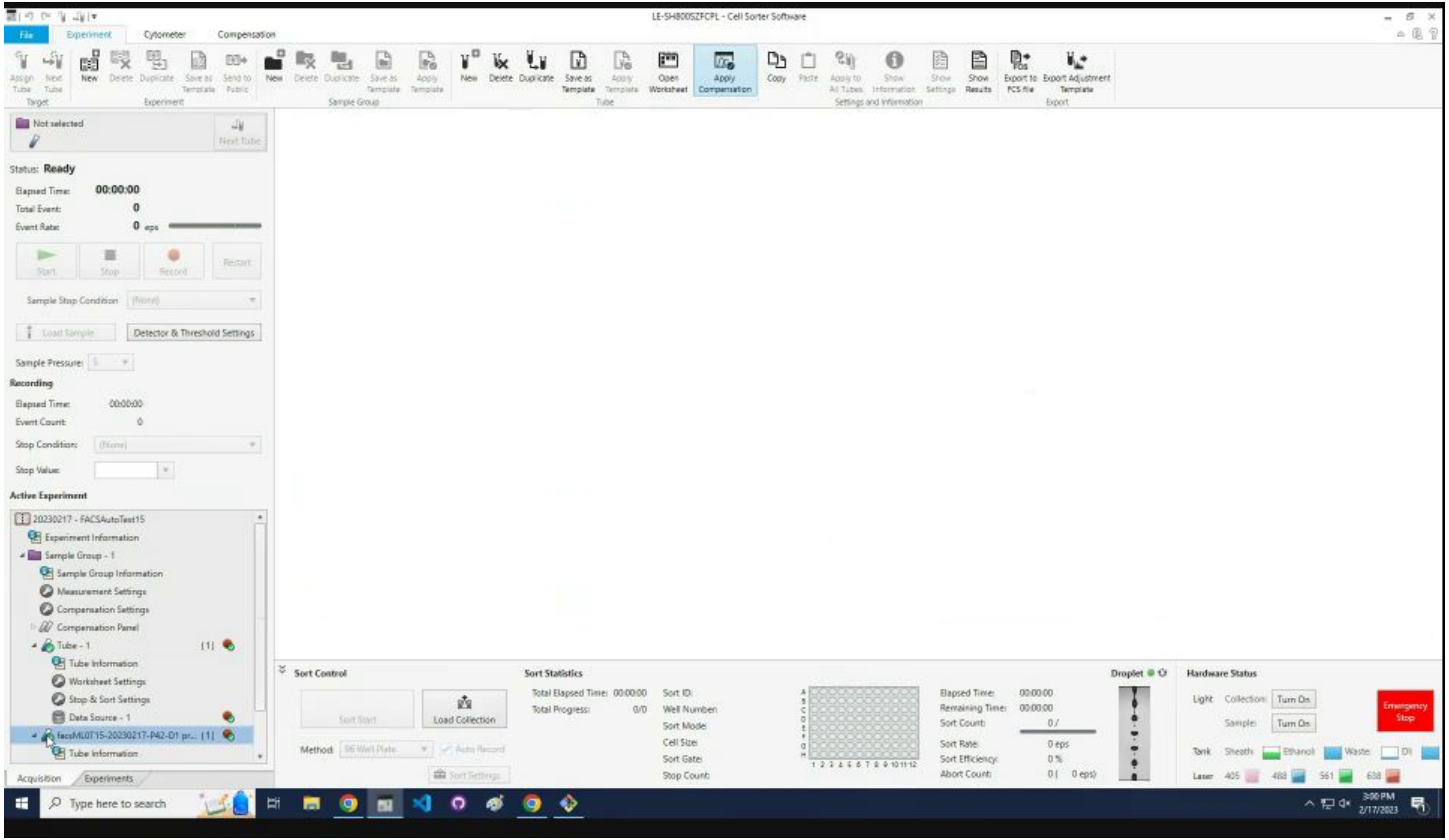

Click on the profile sample in the list.

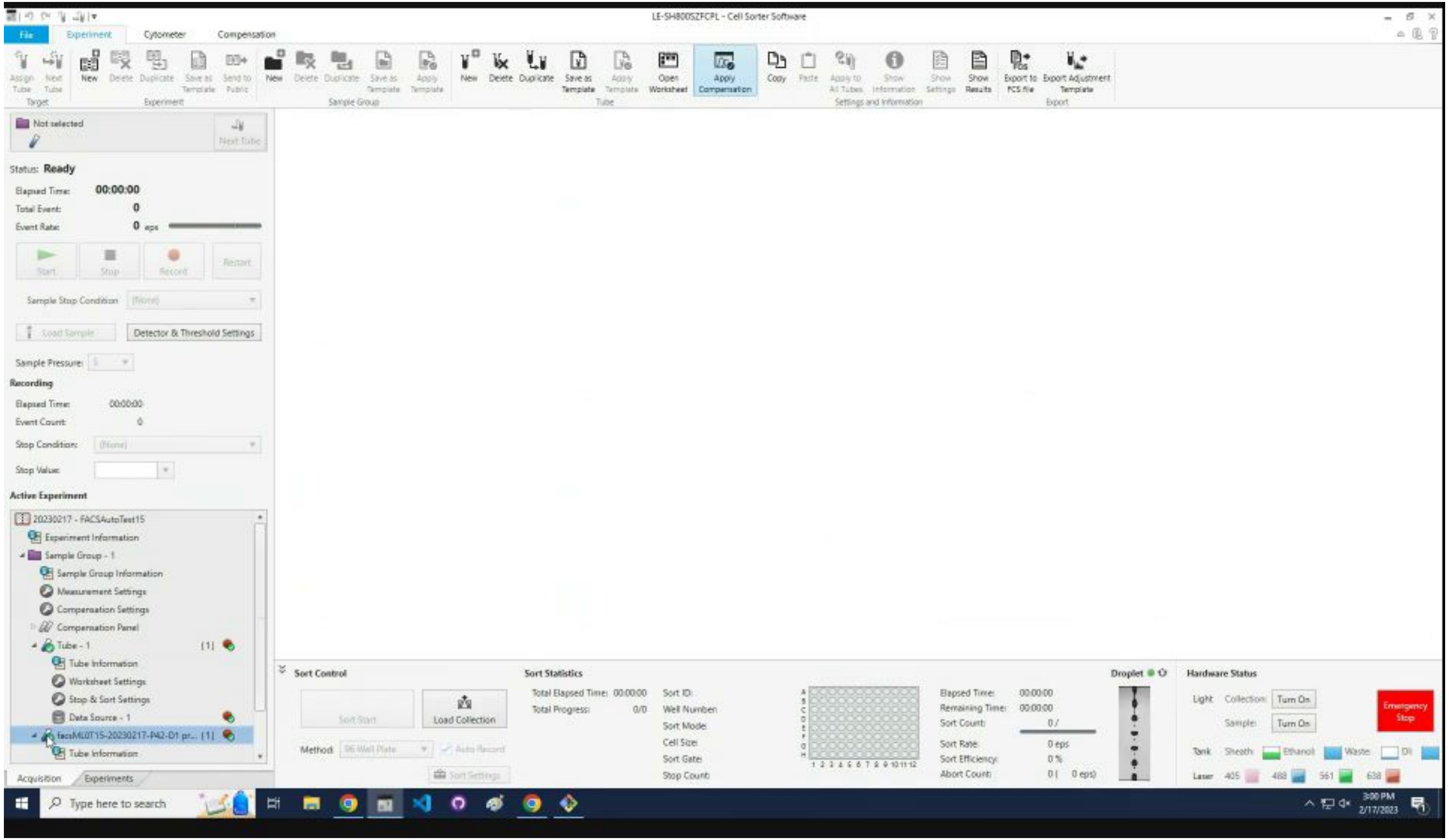

Click the `Duplicate` icon.

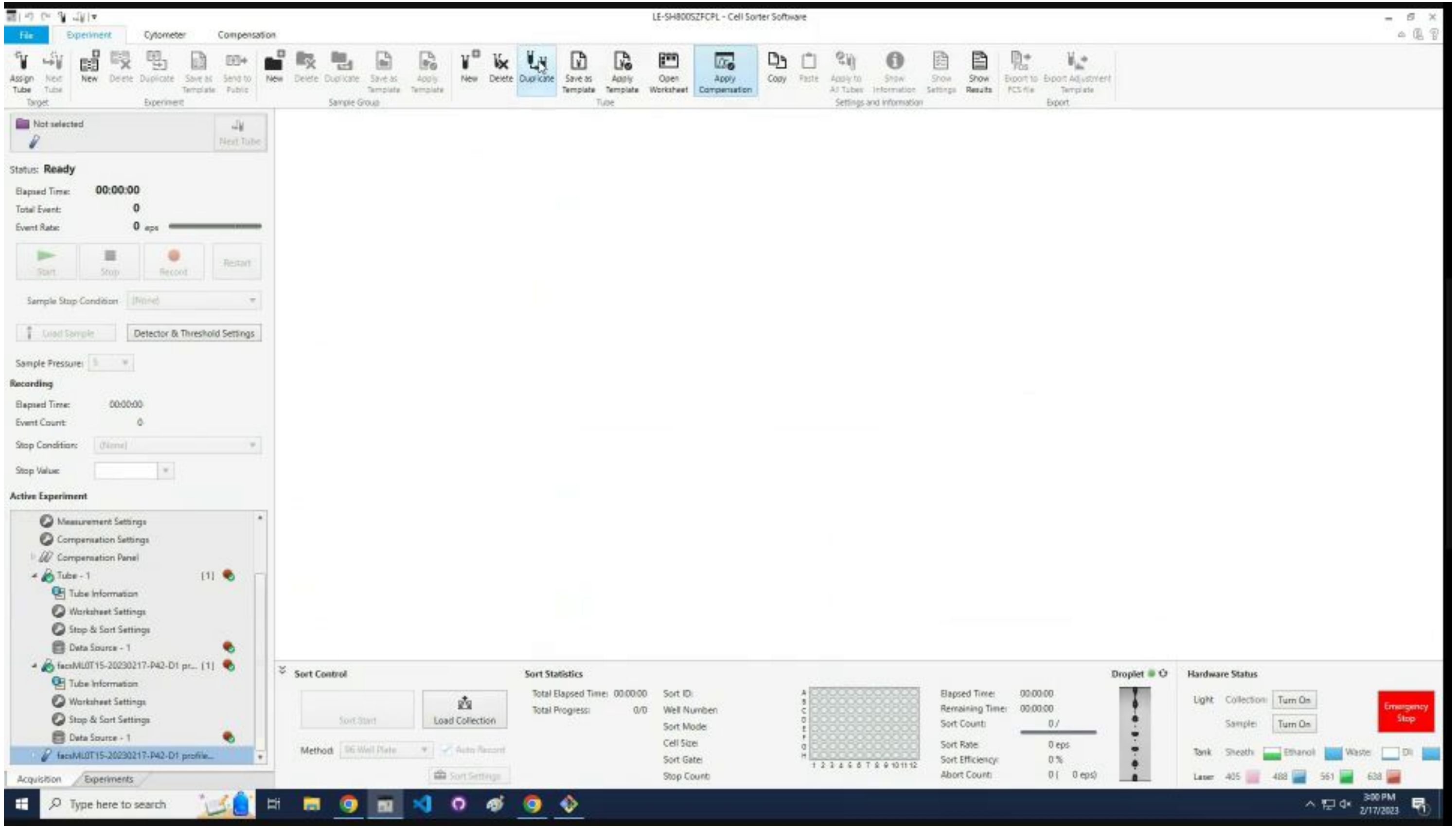

Click the `Assign Tube` icon.

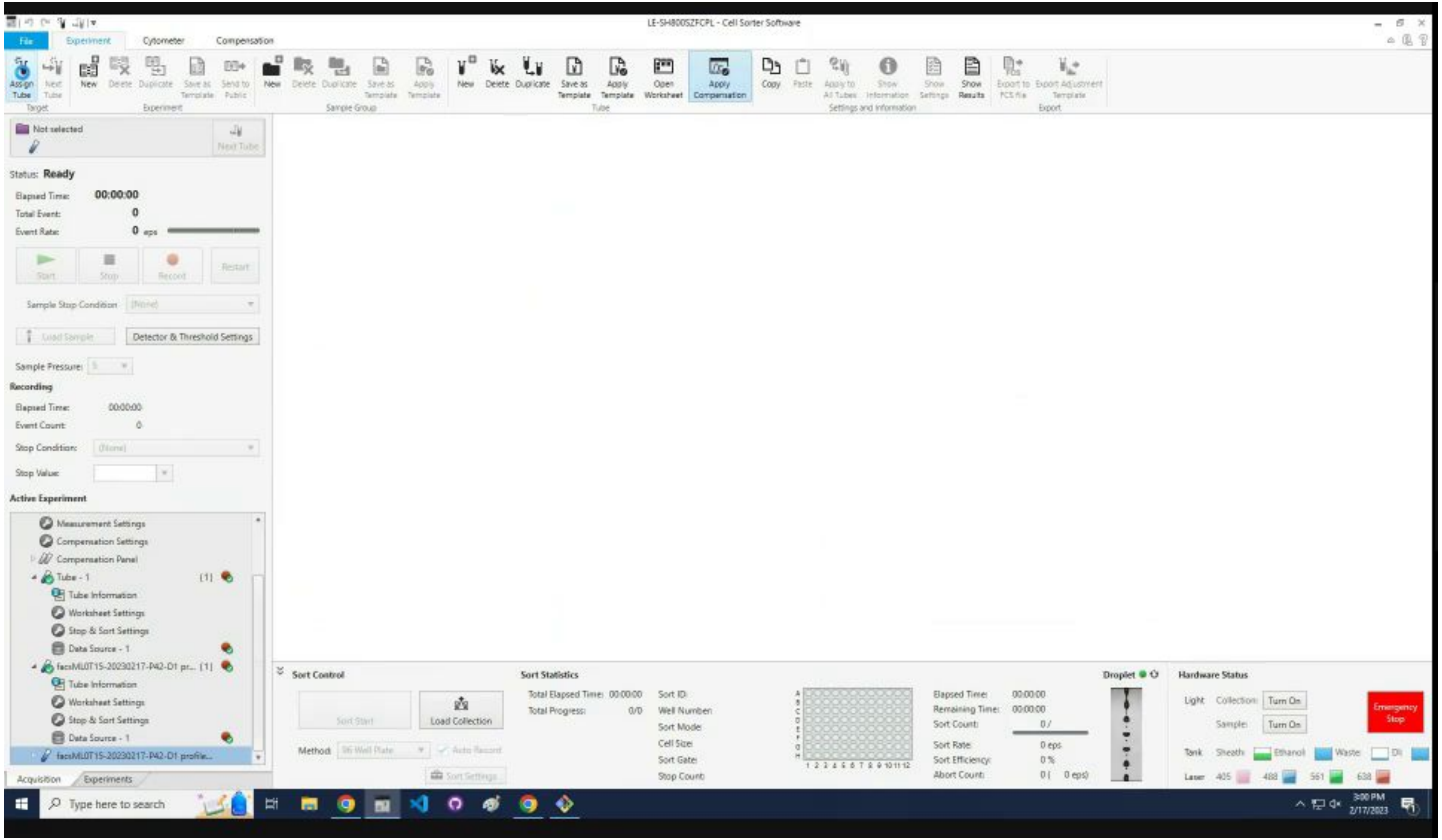

Check that the gate was properly drawn.

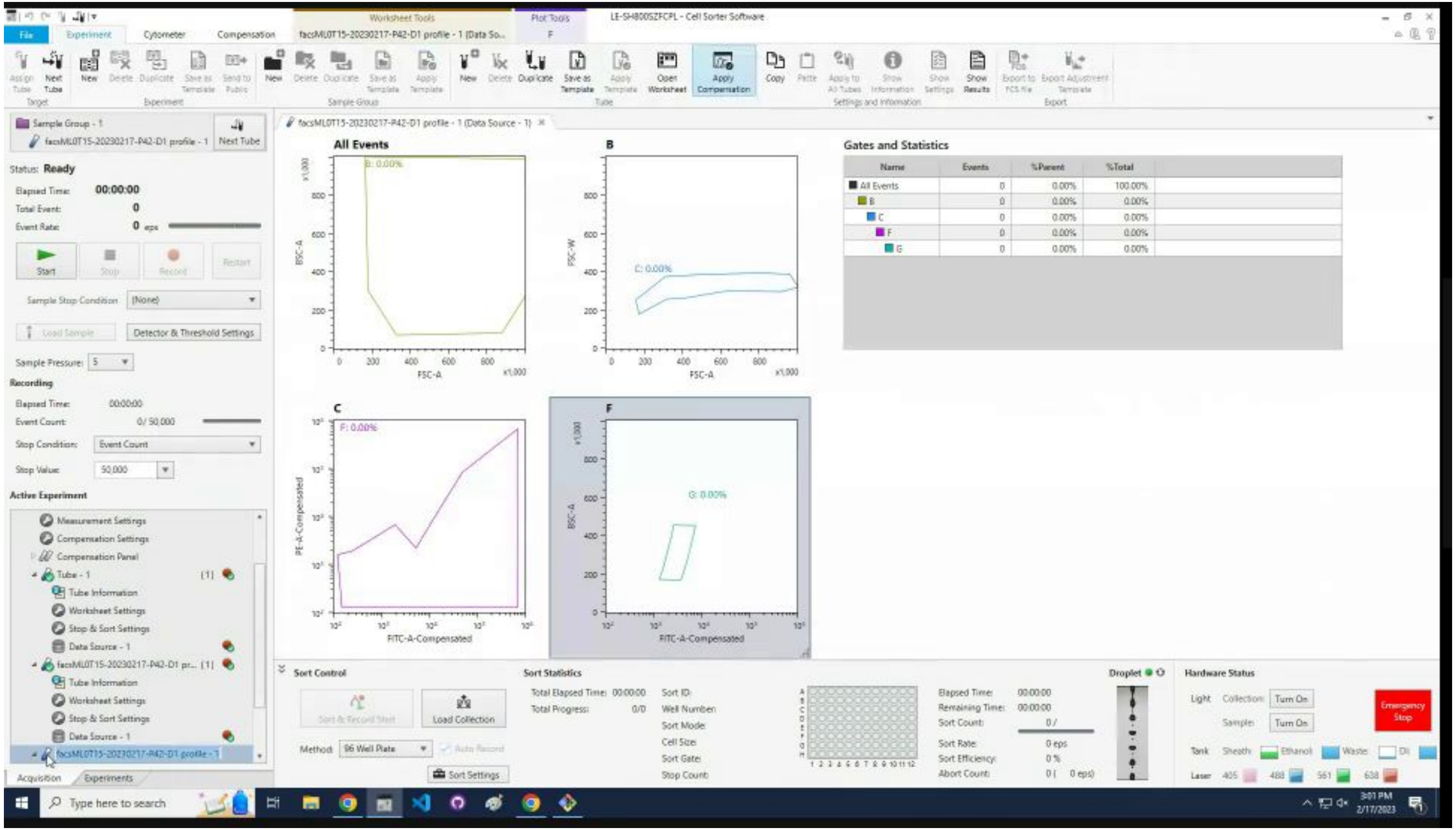

Press `F2` to rename the tube.

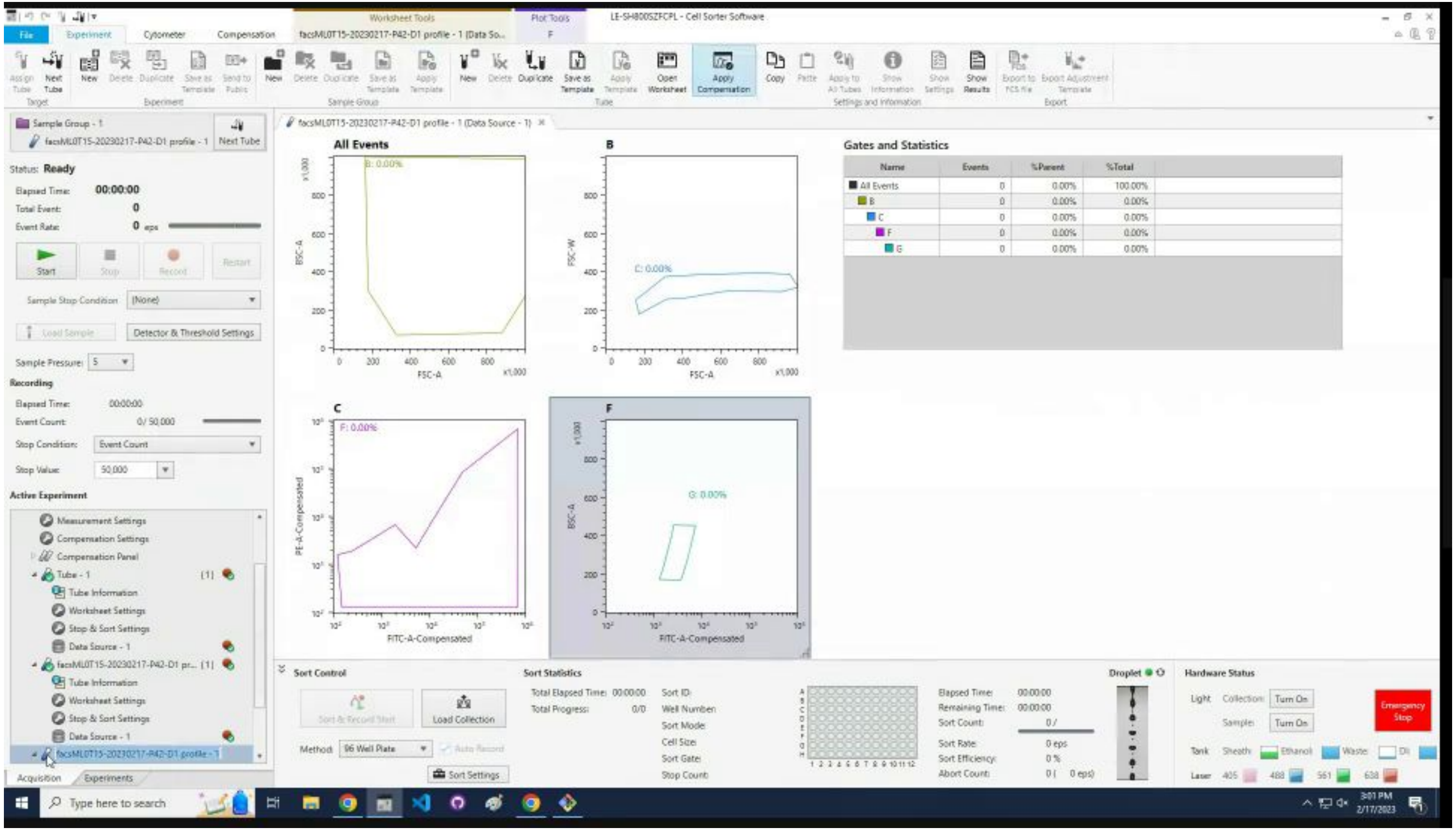

Rename to the sample name appended with sort.

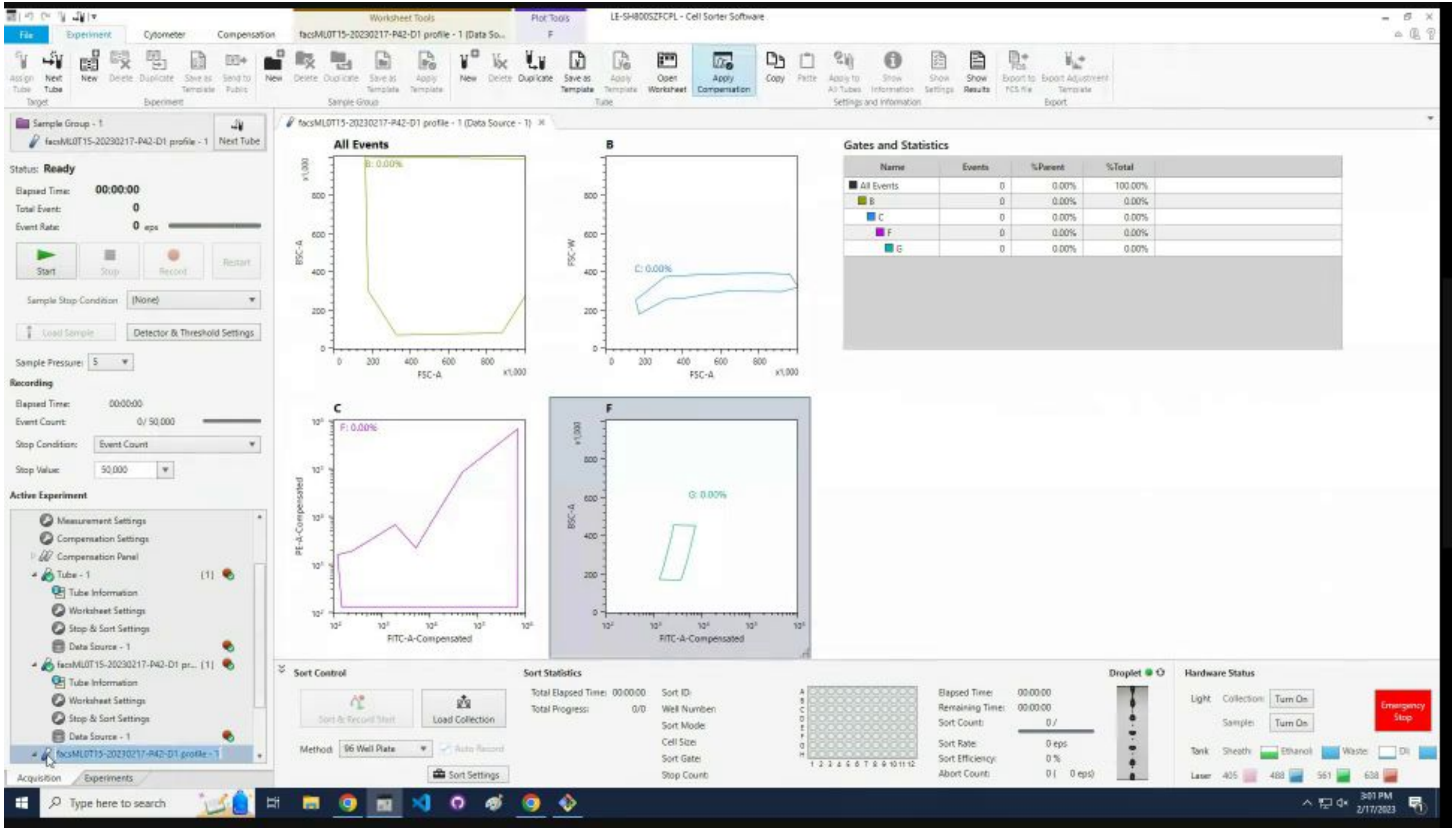

Set the `Sample Pressure` likely to `5`.

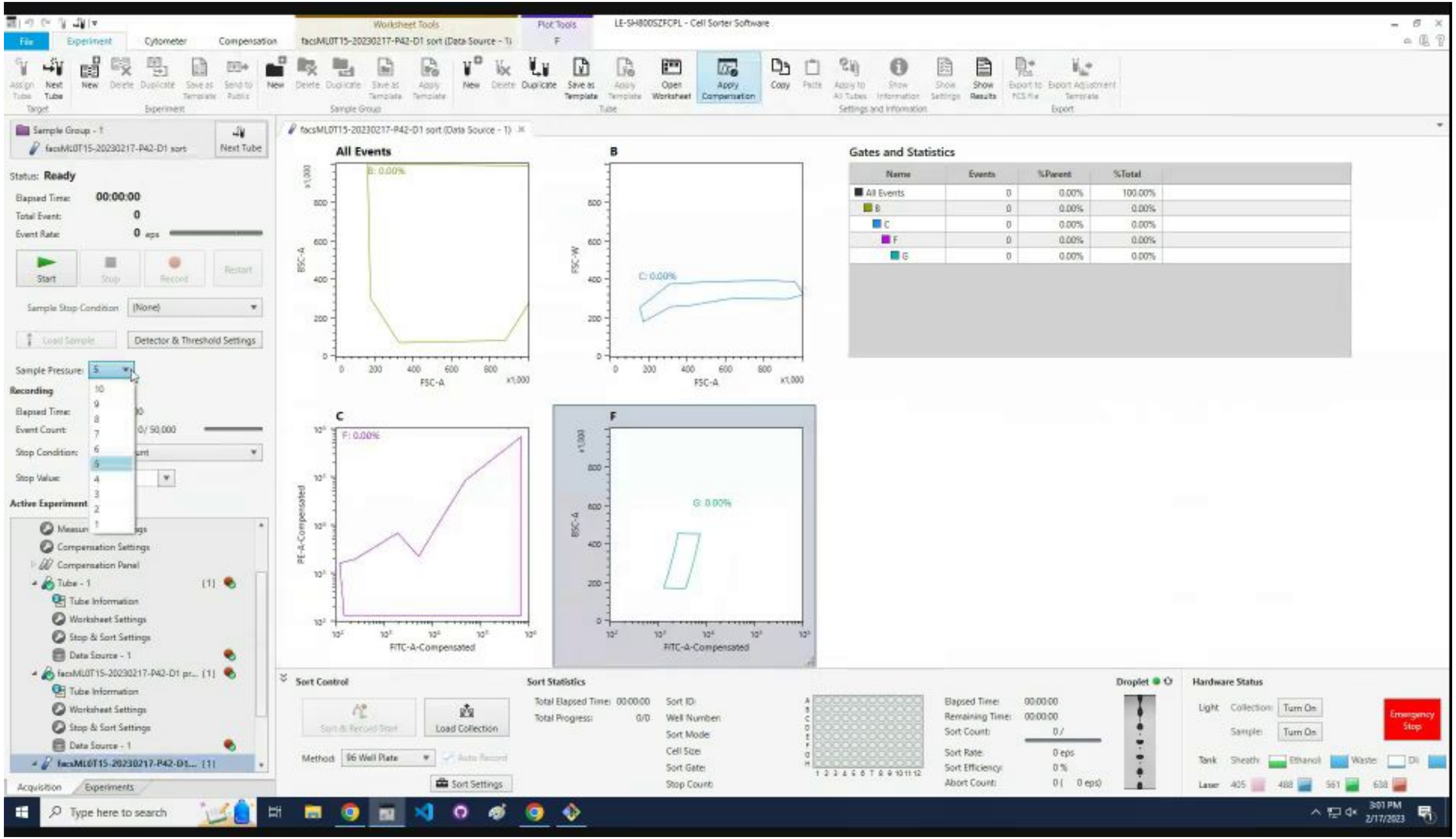

Change the `Stop Condition` to `None`.

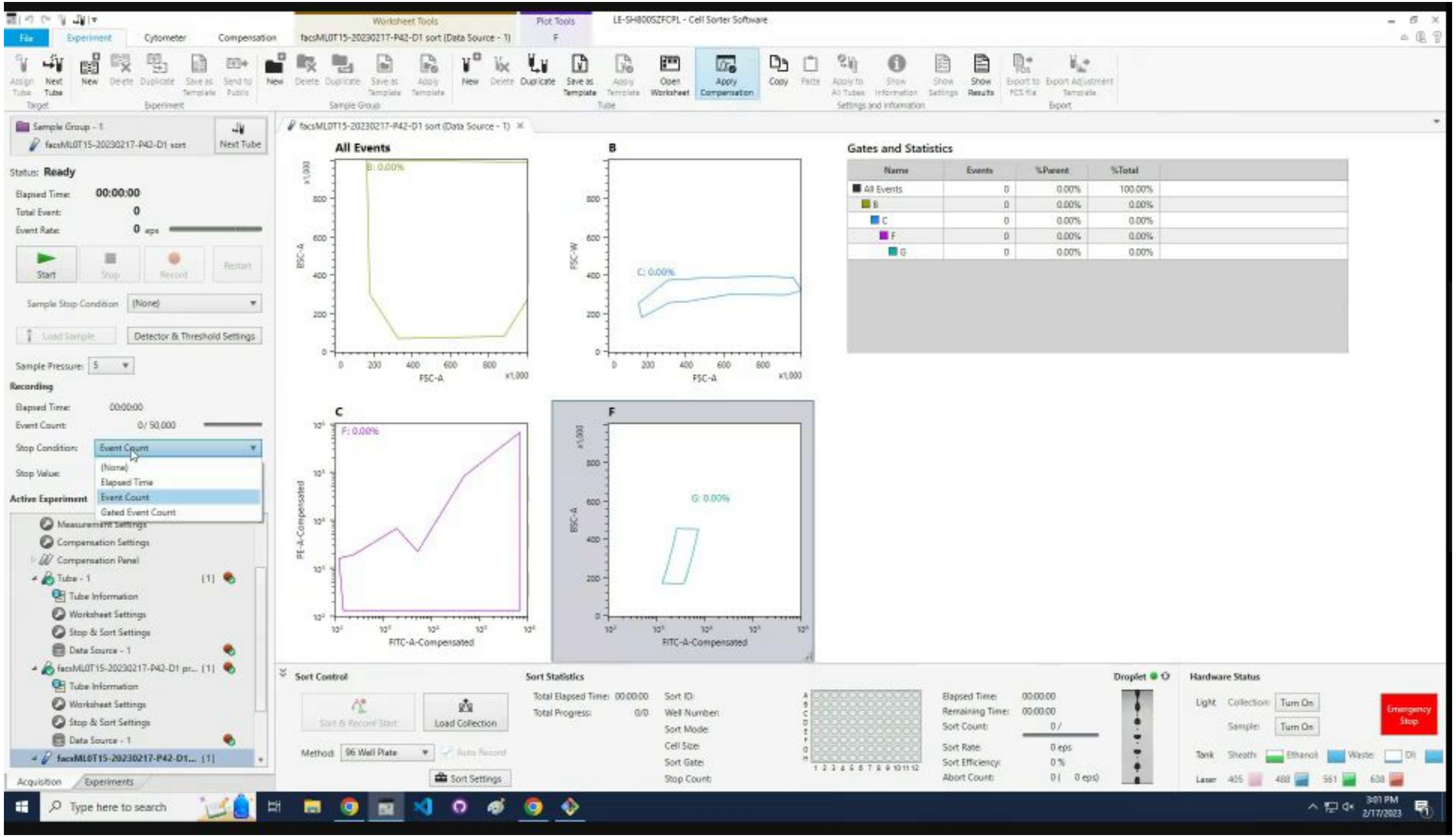

Click `Sort Settings.`

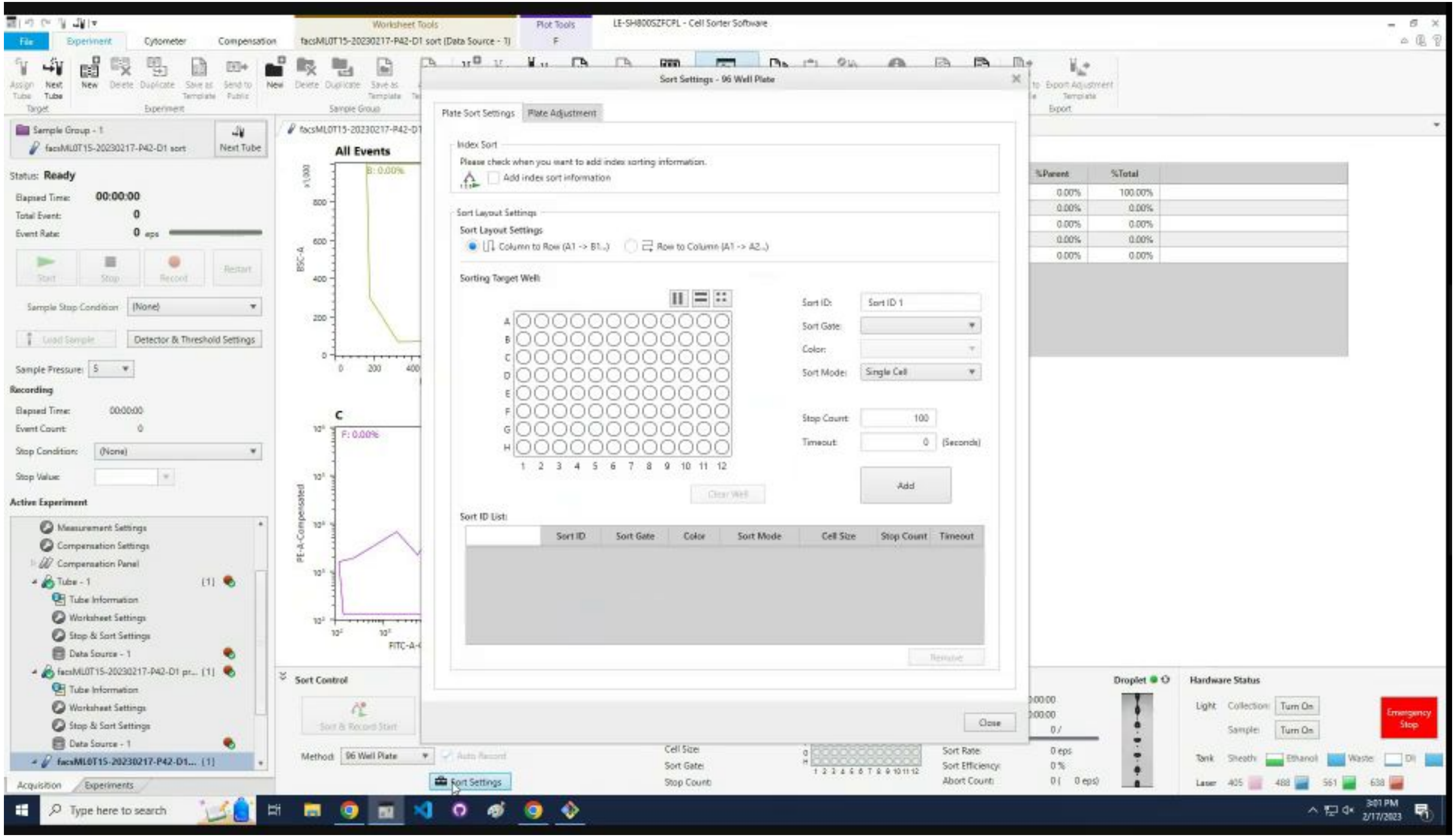

If it is the first sort, it loads without any sort settings.

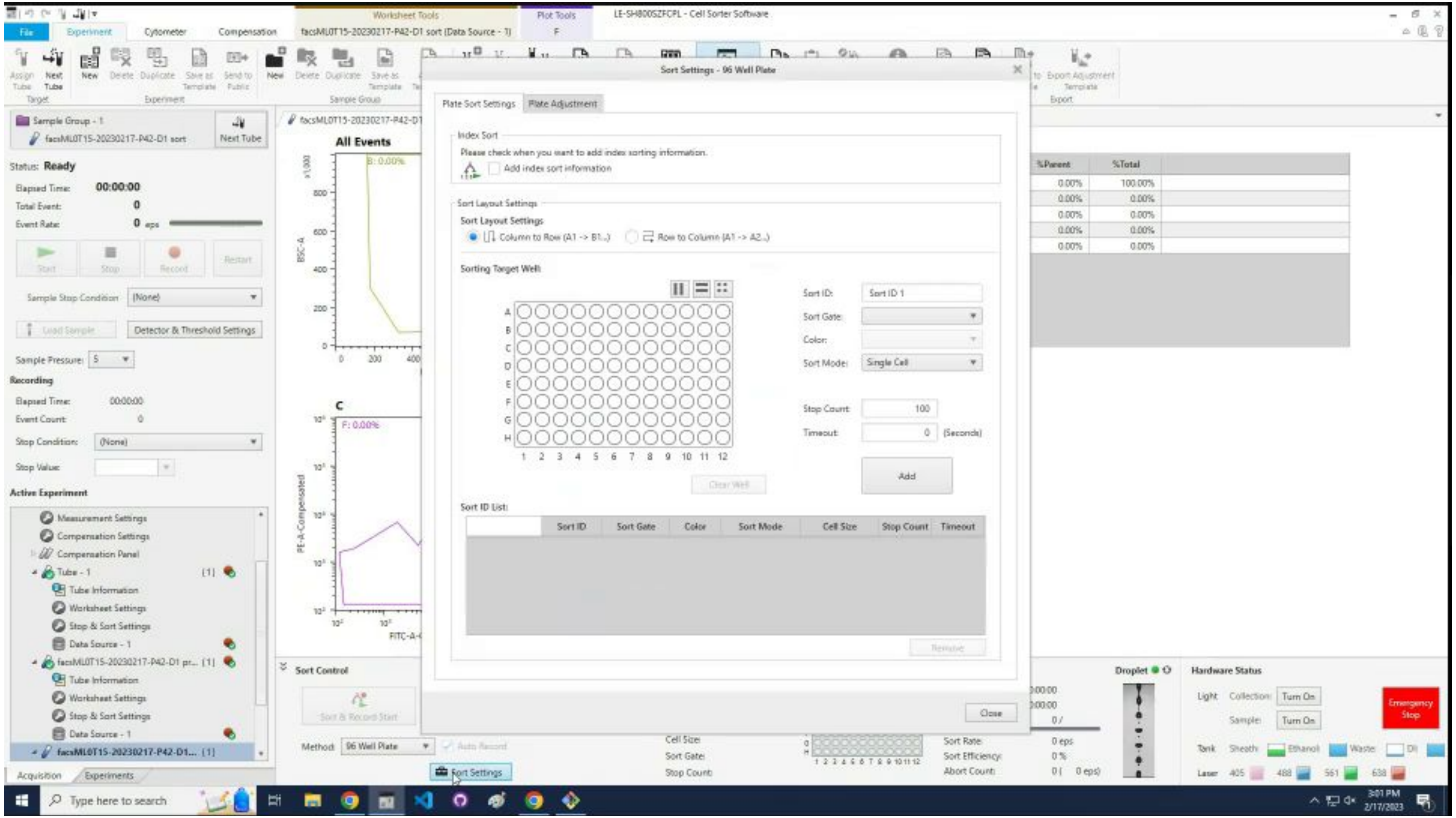

If is not the first sort, it loads with the previous settings.

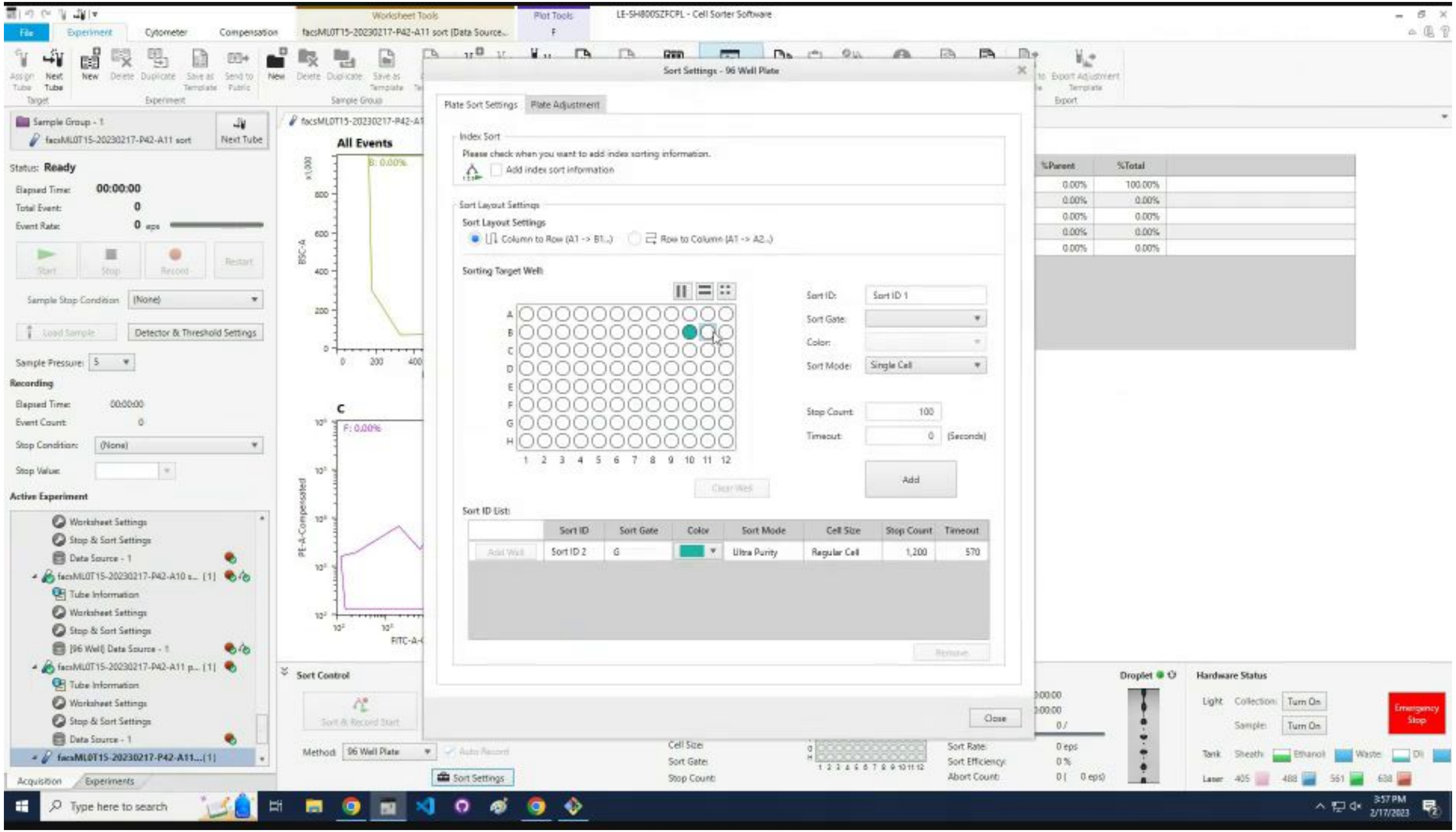

Select the well into which the desired cells should go.

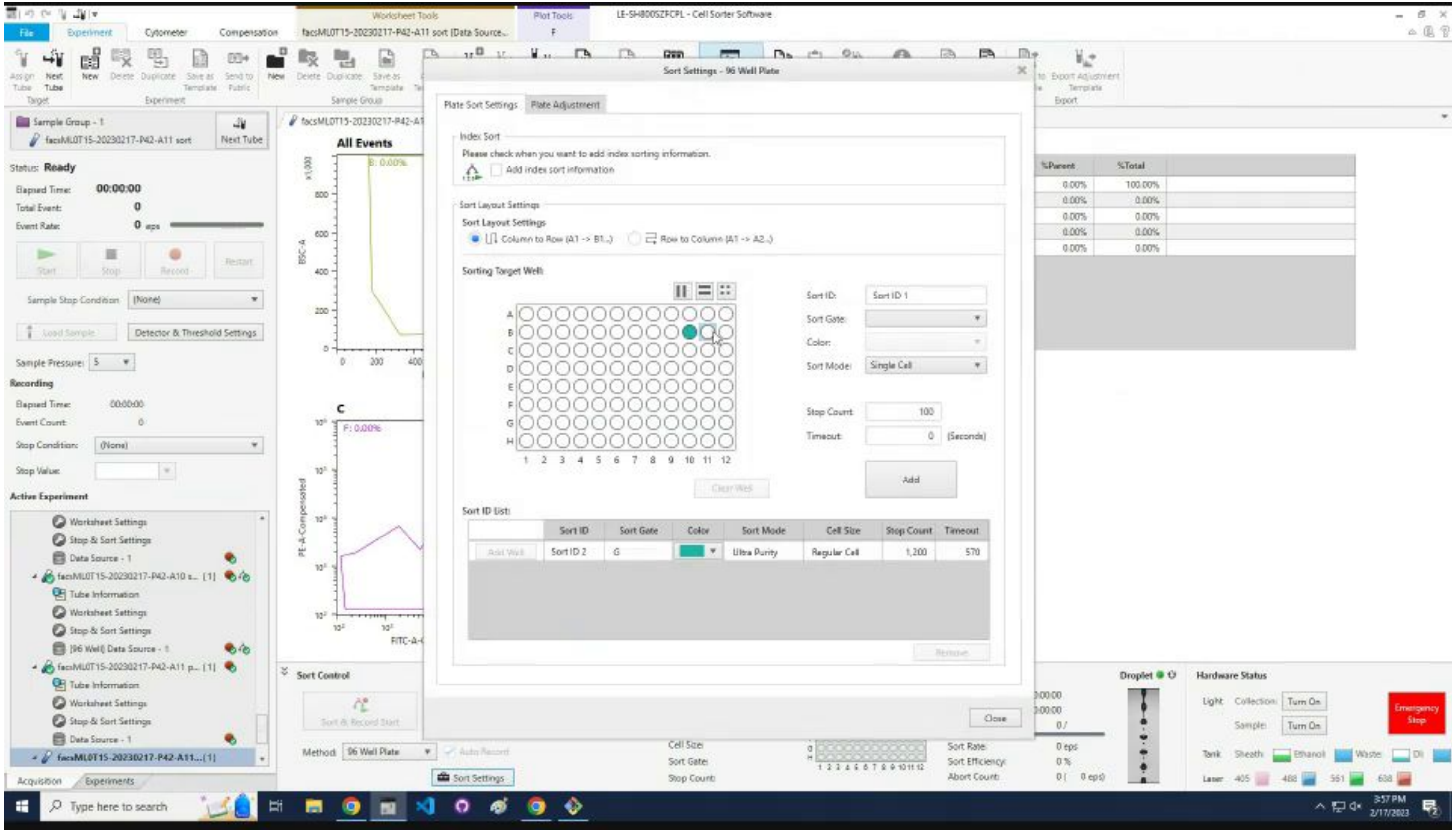

Click the `Sort Gate` drop down.

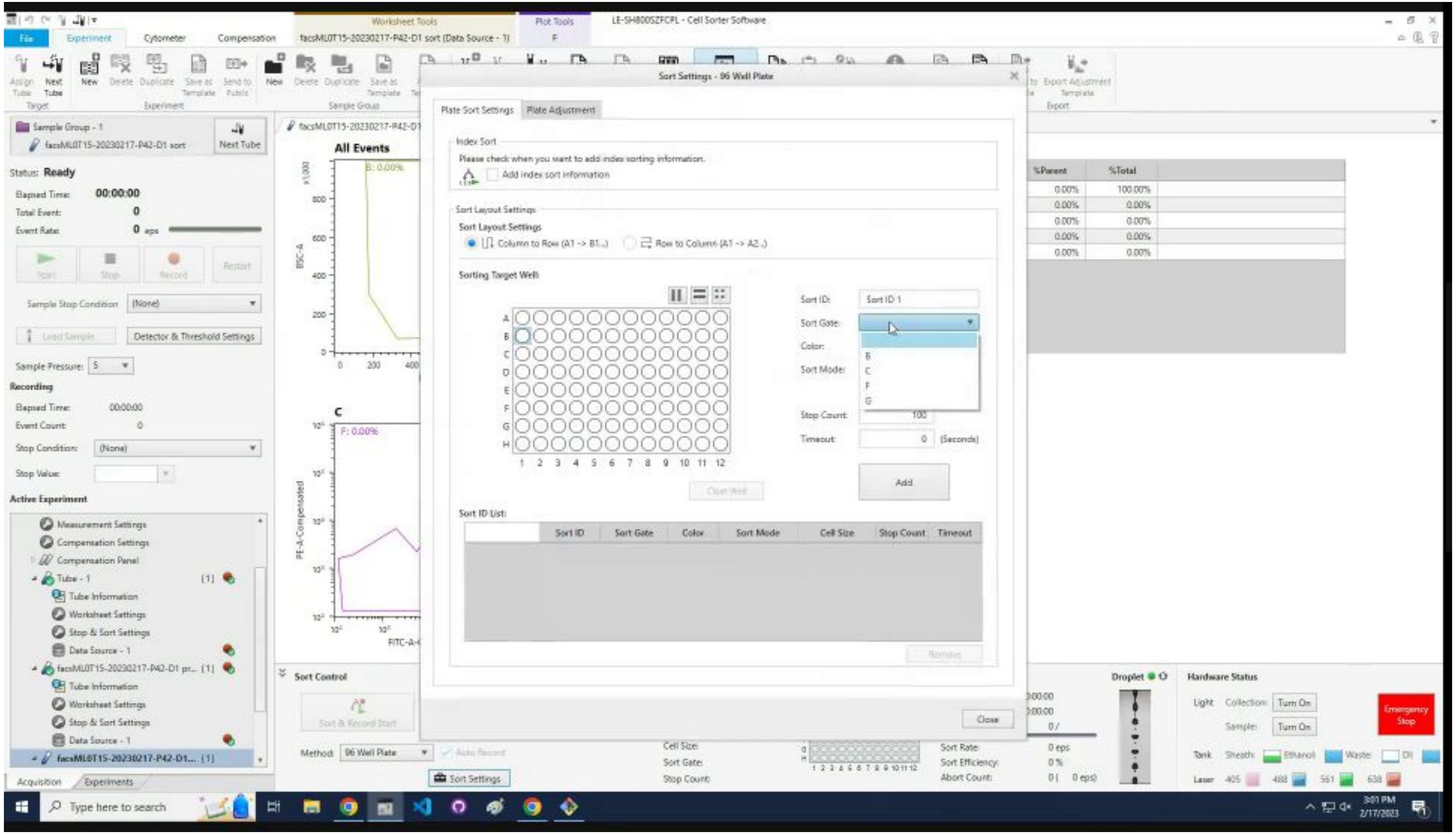

Select the last gate.

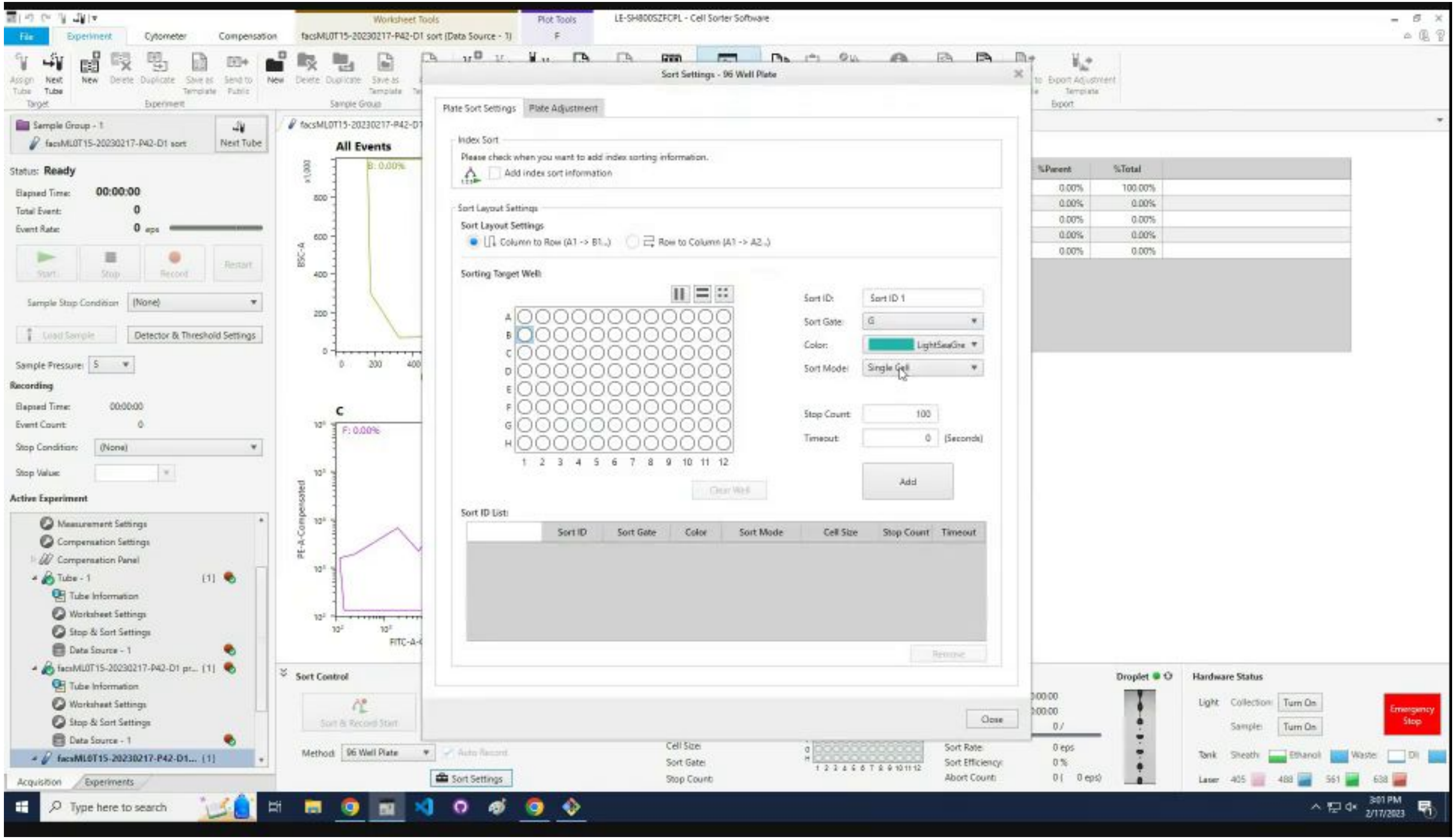

Select the `Sort Mode` drop down.

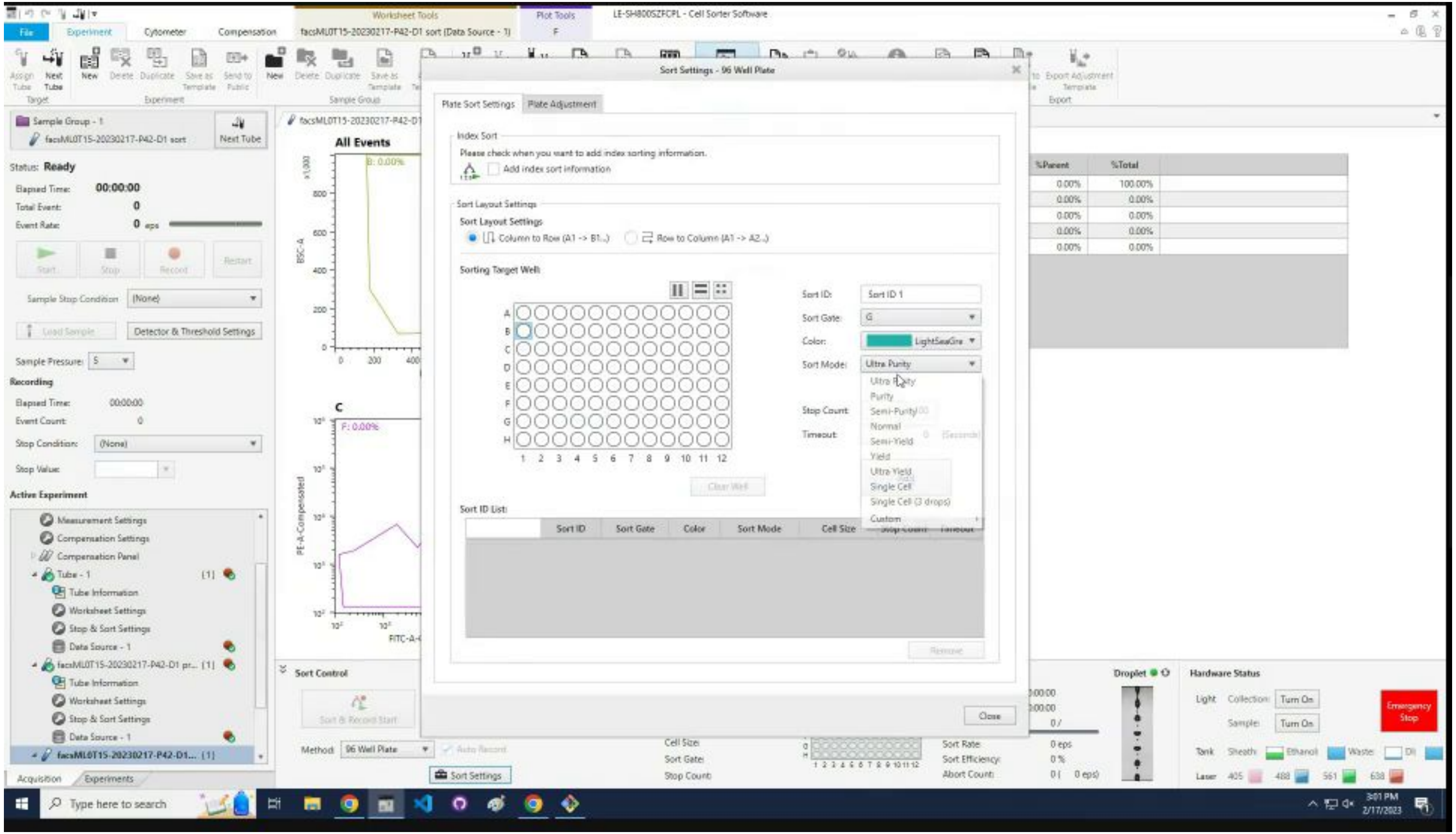

Select `Ultra Purity`.

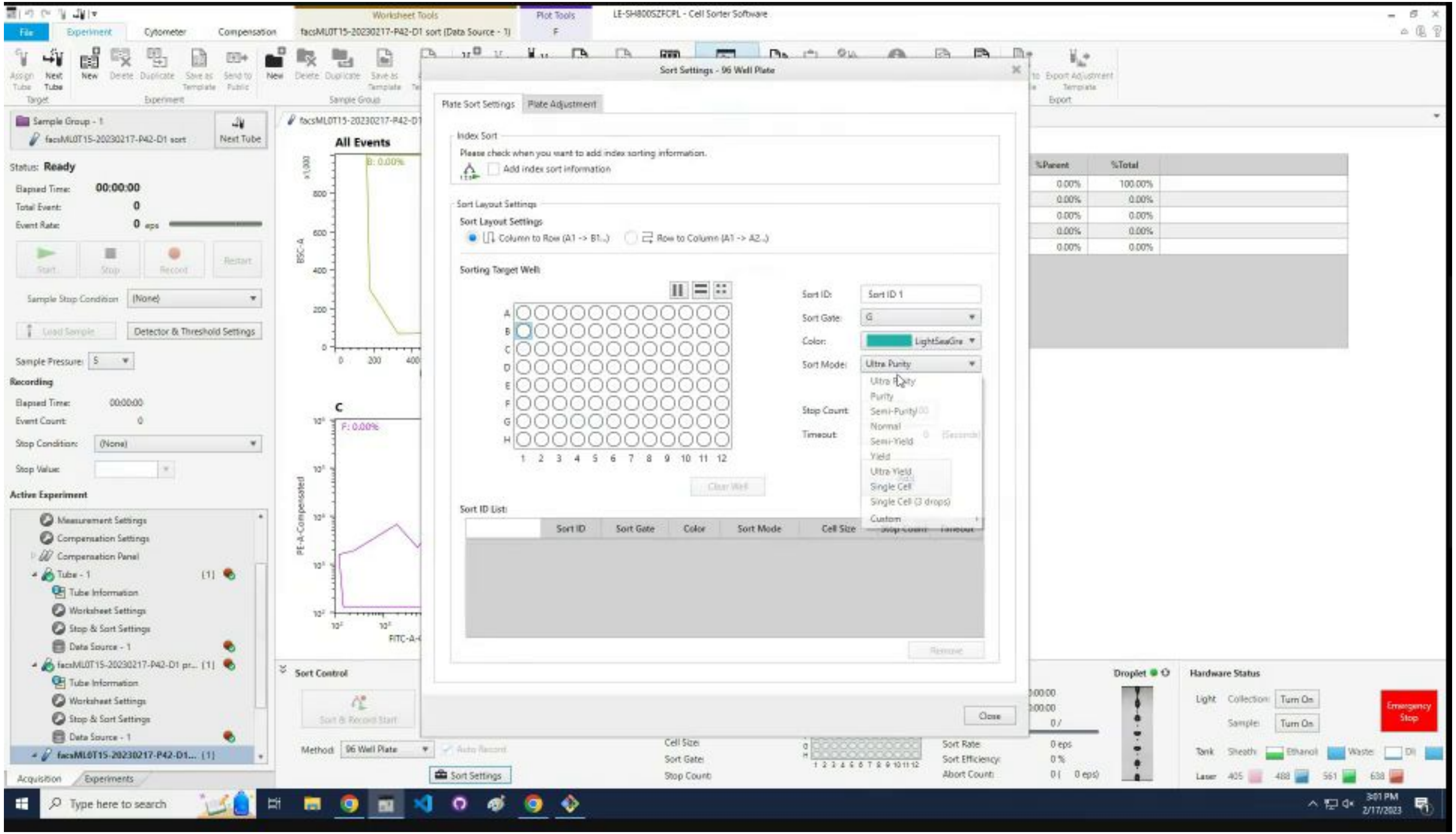

Click in the `Stop Count`.

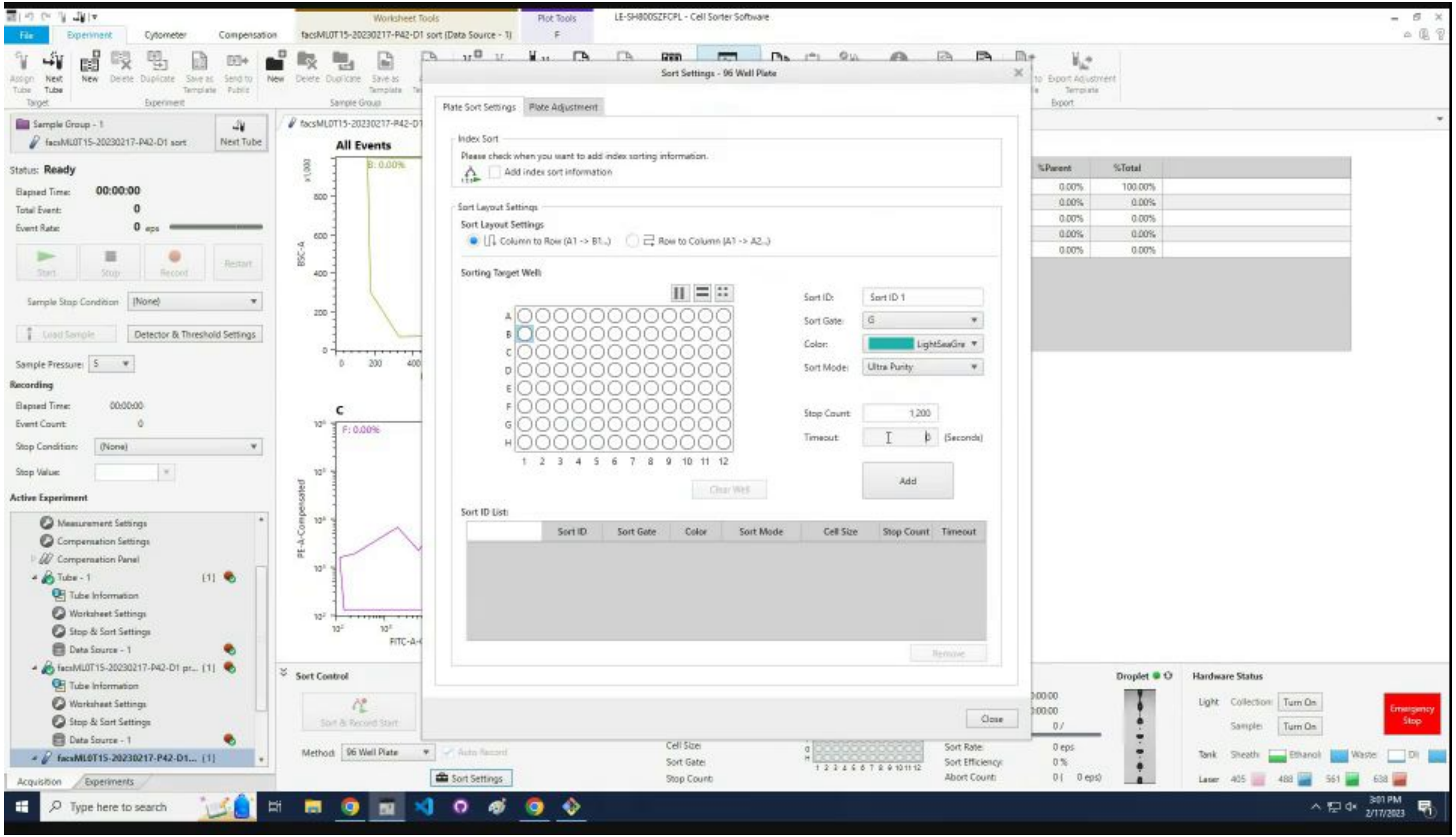

Type in the desired count, likely `1200`.

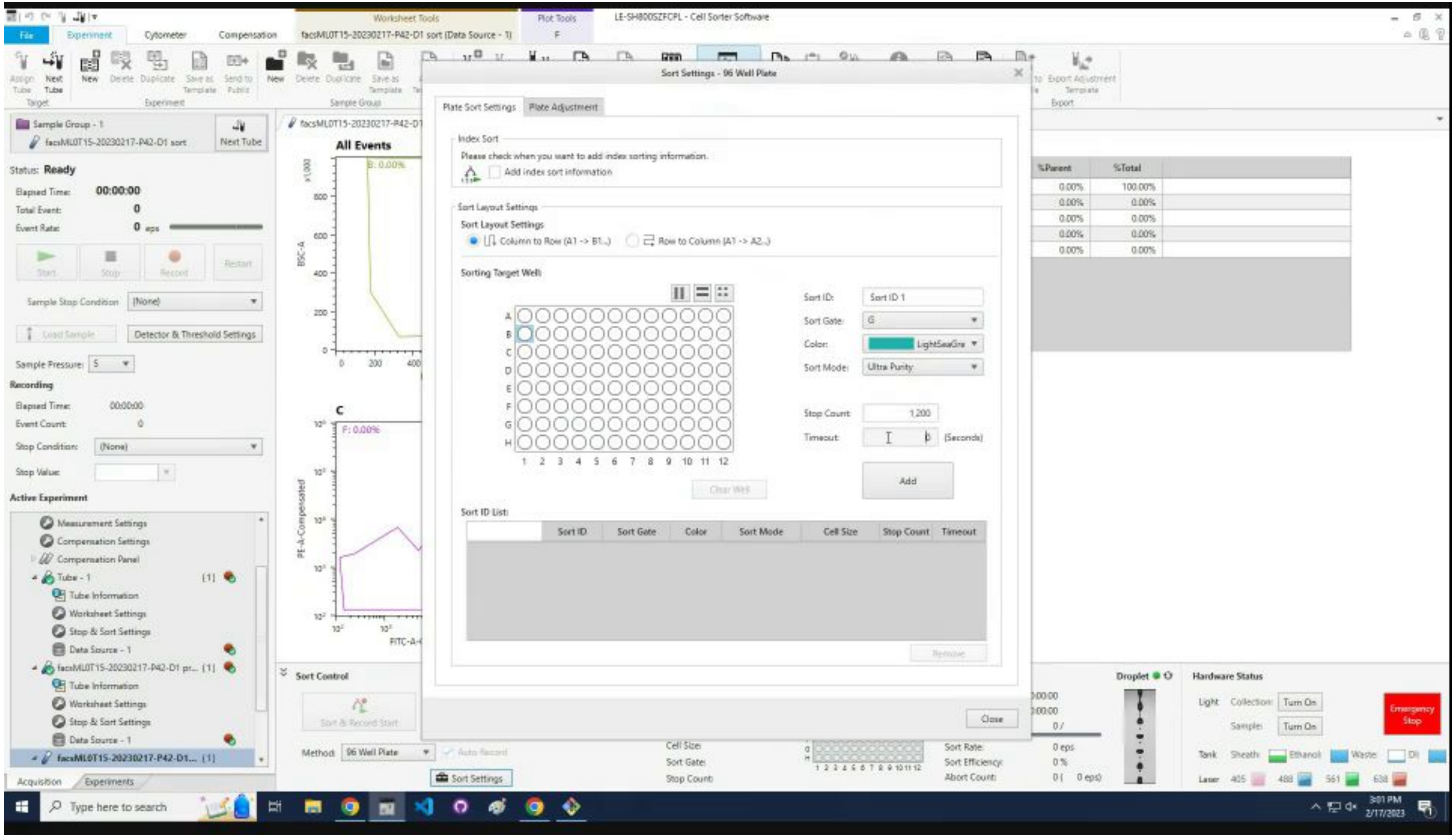

Click in the `Timeout`.

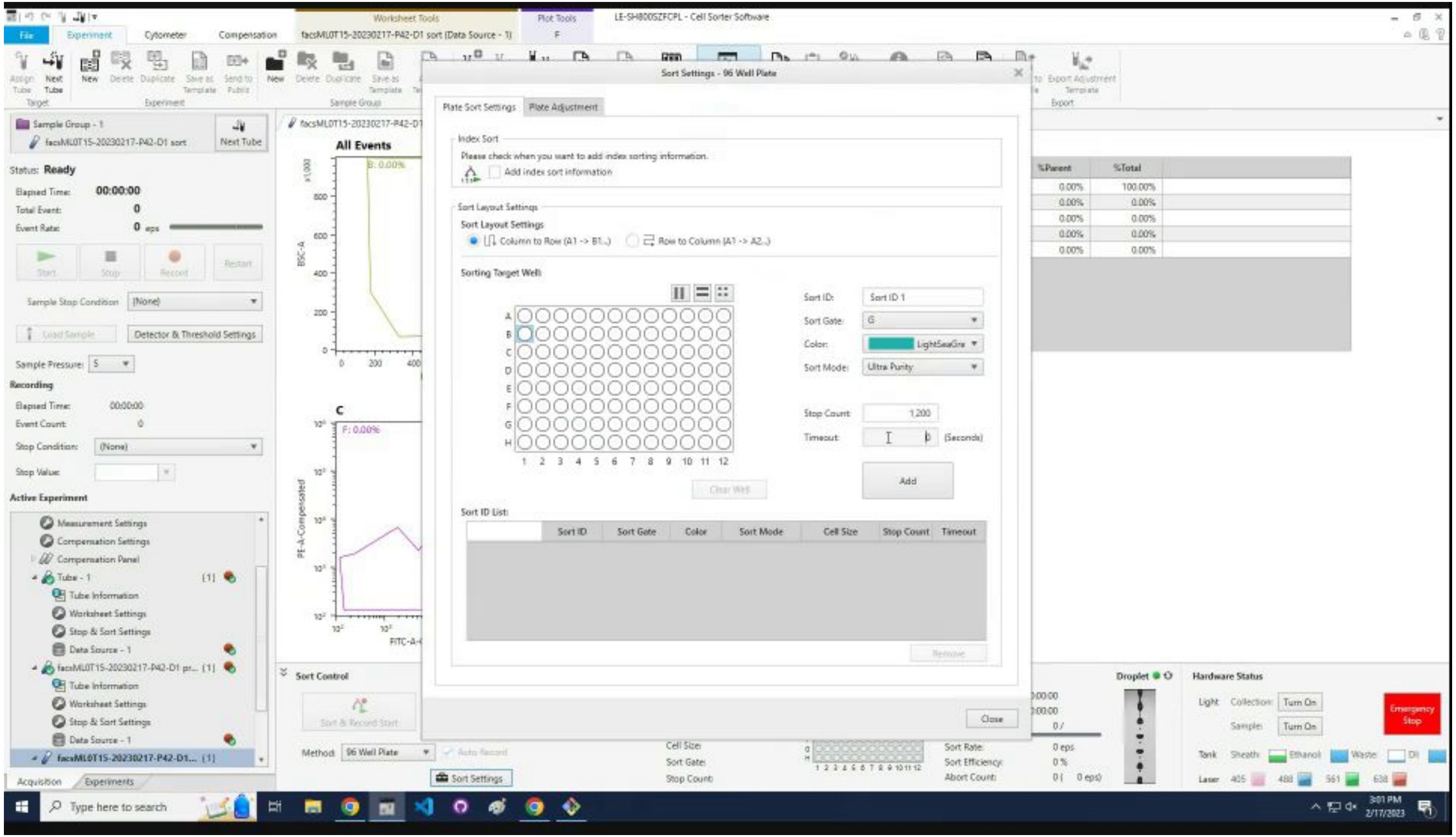

Type in the time, likely `570`.

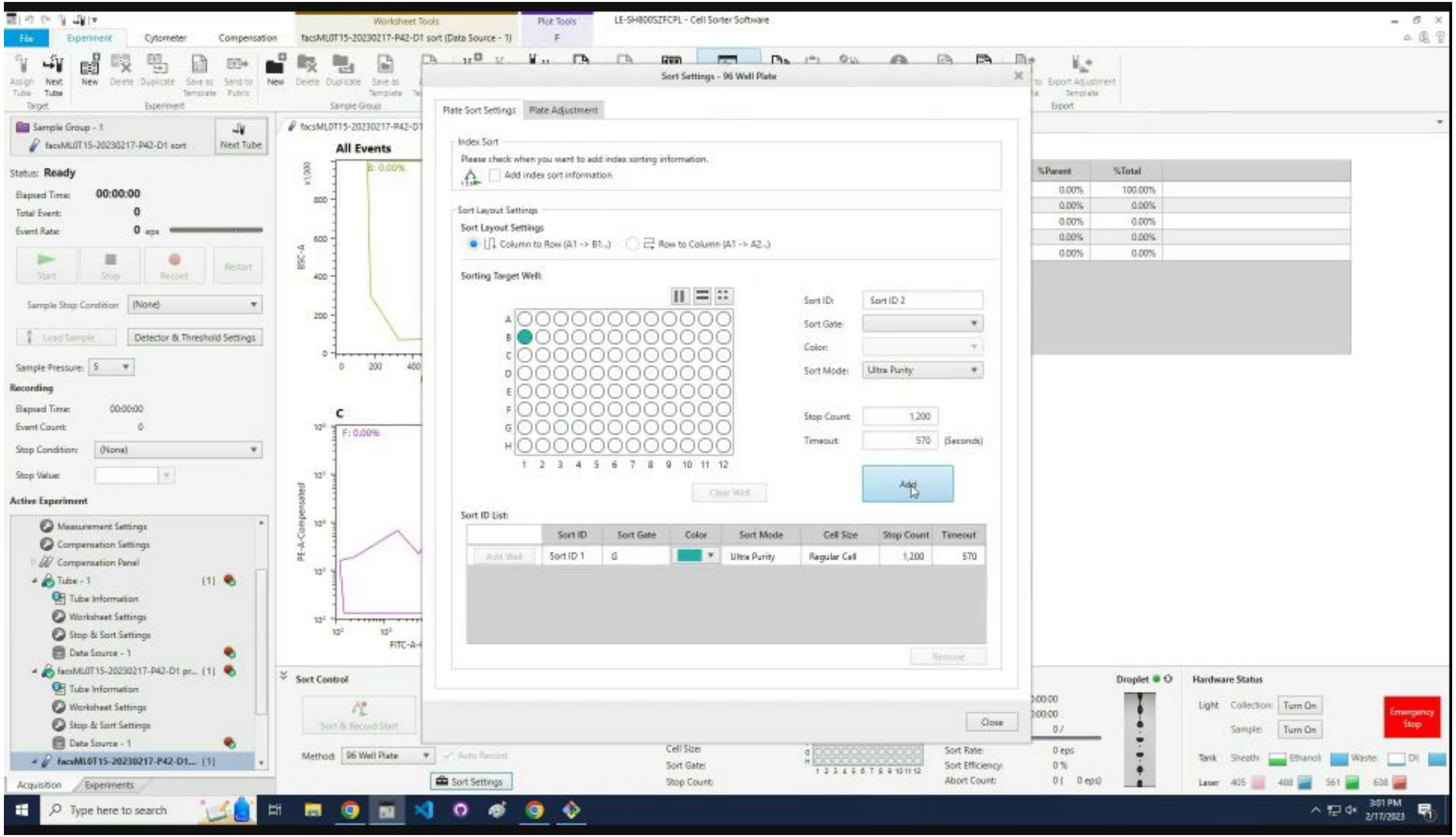

Click `Add`.

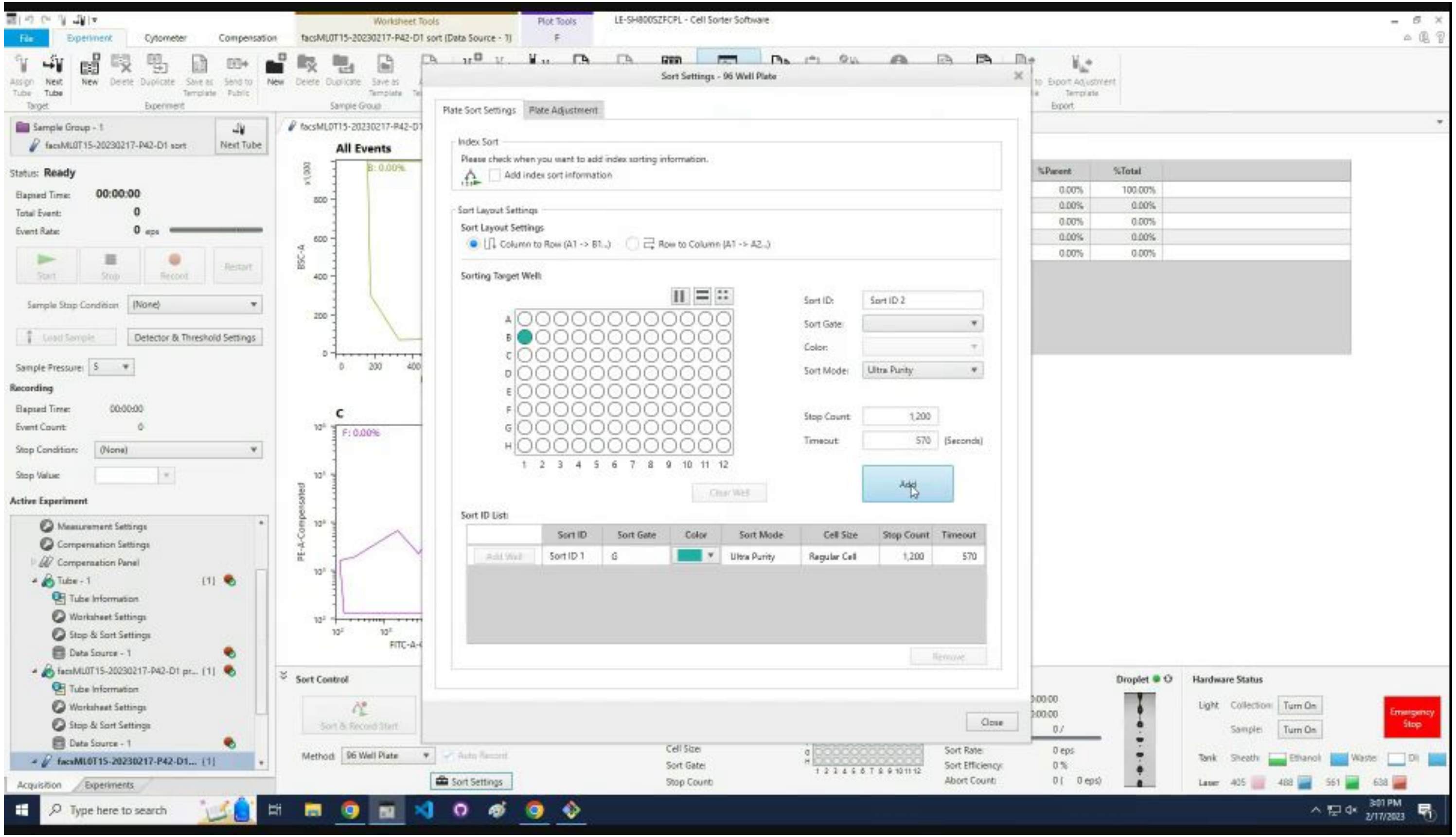
Sort Settings - 96 Well Plate
Plate Sort Settings
Plate Adjustment
Index Sort
Please check when you want to add index sorting information.
Add index sort information
Sort Layout Settings
Column to Row (A1 -> B1...)
Row to Column (A1 -> A2...)
Sorting Target Well:
Sort ID:
Sort ID 2
Sort Gate:
Color:
Sort Mode:
Ultra Purity
Stop Count:
1,200
Timeout:
570
(Seconds)
Add
Clear Well
Sort ID List:
Sort ID
Sort Gate
Color
Sort Mode
Cell Size
Stop Count
Timeout
Add Well
Sort ID 1
G
Ultra Purity
Regular Cell
1,200
570
Remove
Close
All Events
Sort Control
Hardware Status
Emergency Stop

If it is the first sort, go to completing Sort Settings slide.

If is not the first sort, go to next slide.

Click the top entry in the `Sort ID List`.

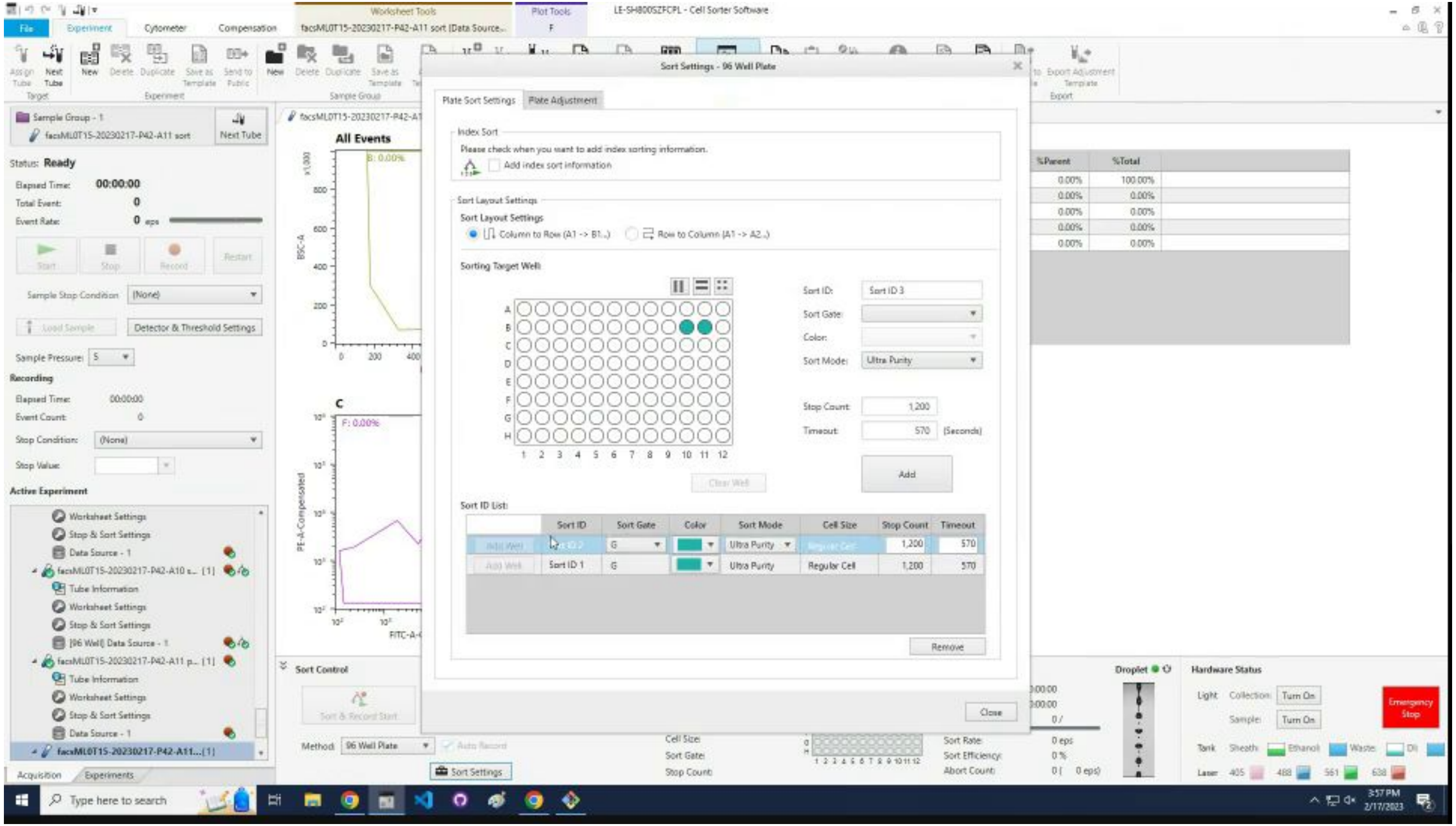

Click `Remove`.

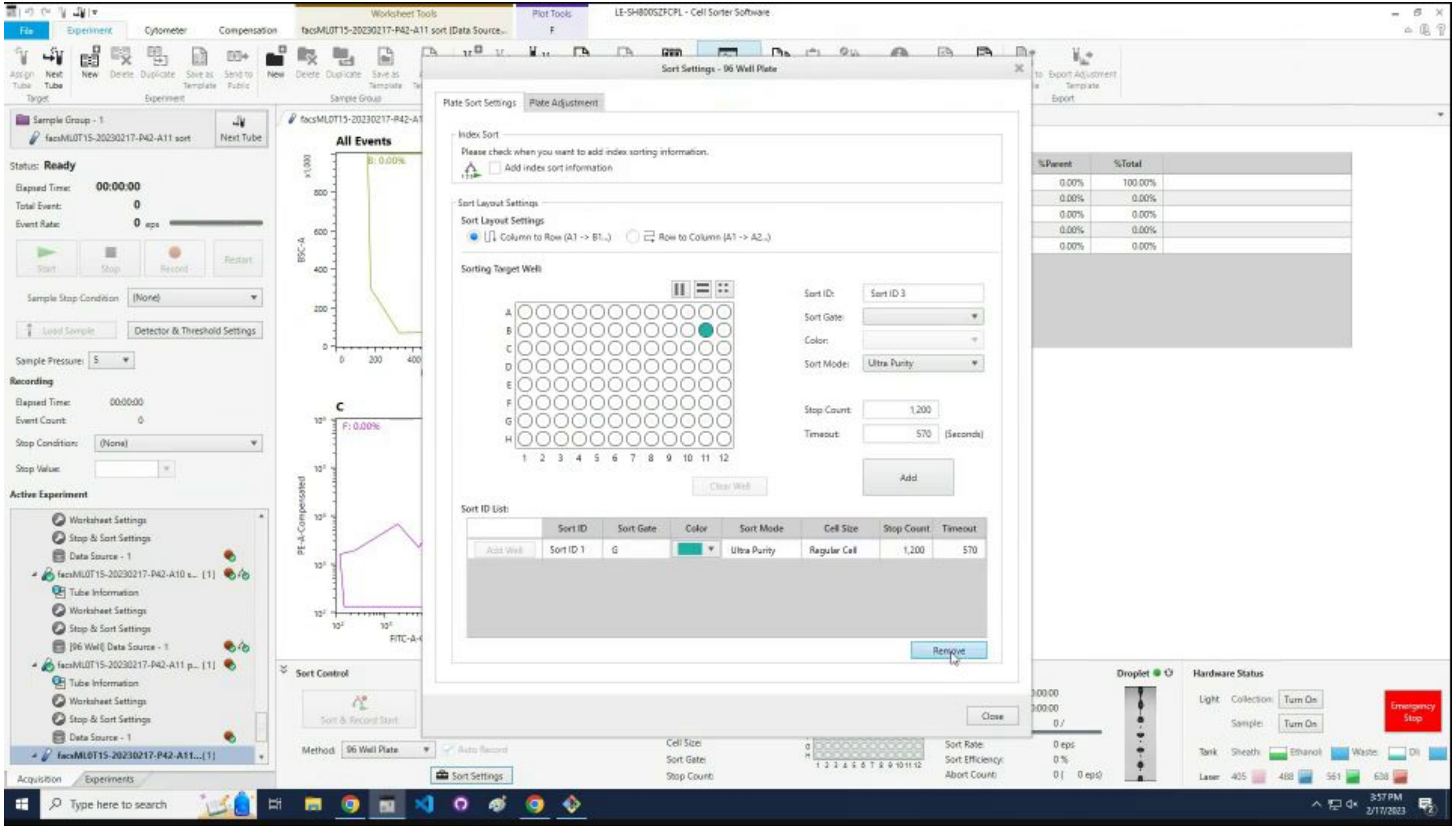

Click `Close`.

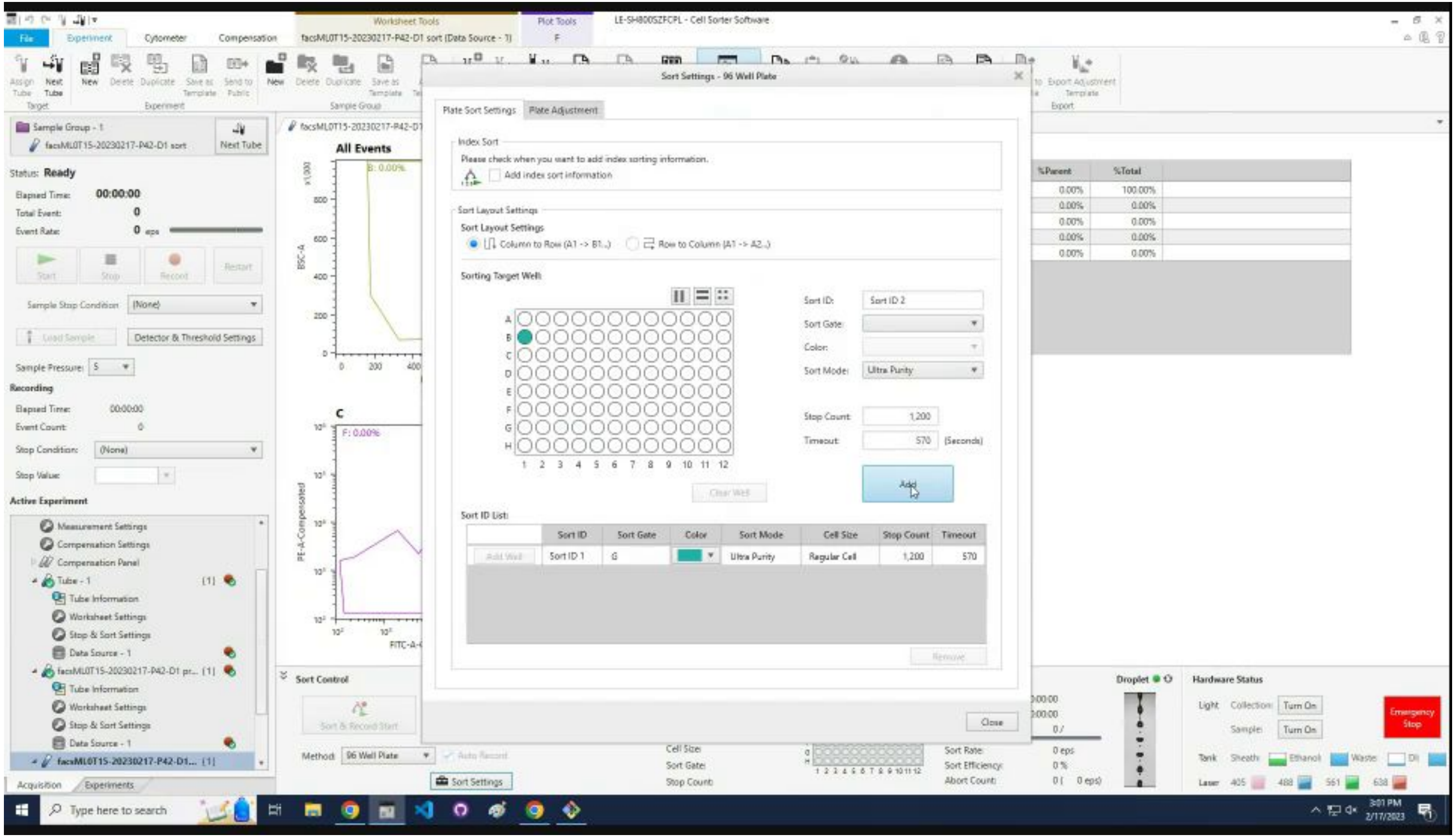

Sort Settings - 96 Well Plate
Plate Sort Settings
Plate Adjustment
Index Sort
Please check when you want to add index sorting information.
Add index sort information
Sort Layout Settings
Column to Row (A1 -> B1...)
Row to Column (A1 -> A2...)
Sorting Target Well:
Sort ID:
Sort ID 2
Sort Gate:
Color:
Sort Mode:
Ultra Purity
Stop Count:
1,200
Timeout:
570
(Seconds)
Add
Clear Well
Sort ID List:
Sort ID
Sort Gate
Color
Sort Mode
Cell Size
Stop Count
Timeout
Sort ID 1
G
Ultra Purity
Regular Cell
1,200
570
Remove
Close

Click `Load Collection`.

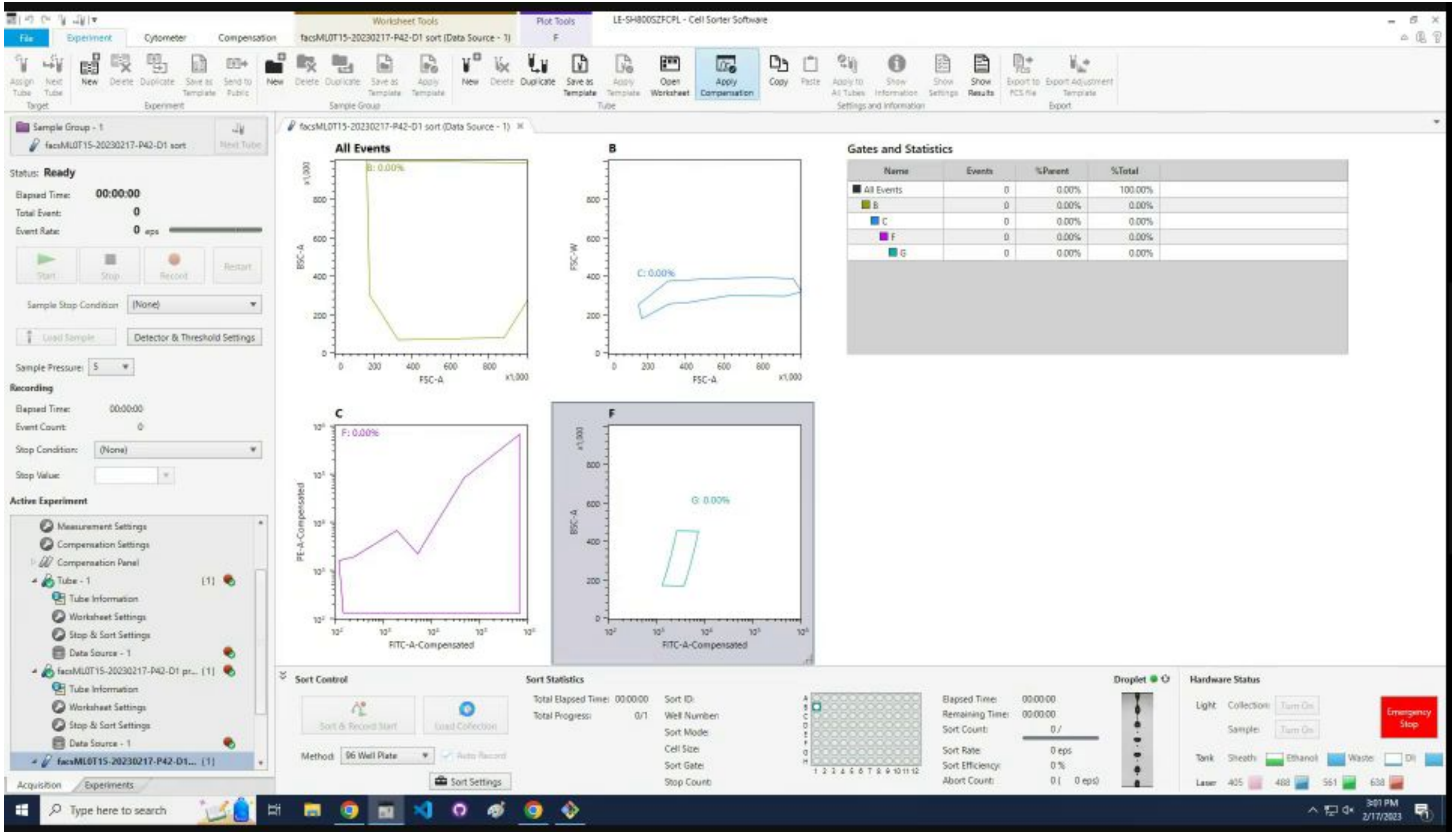

Click `Start`.

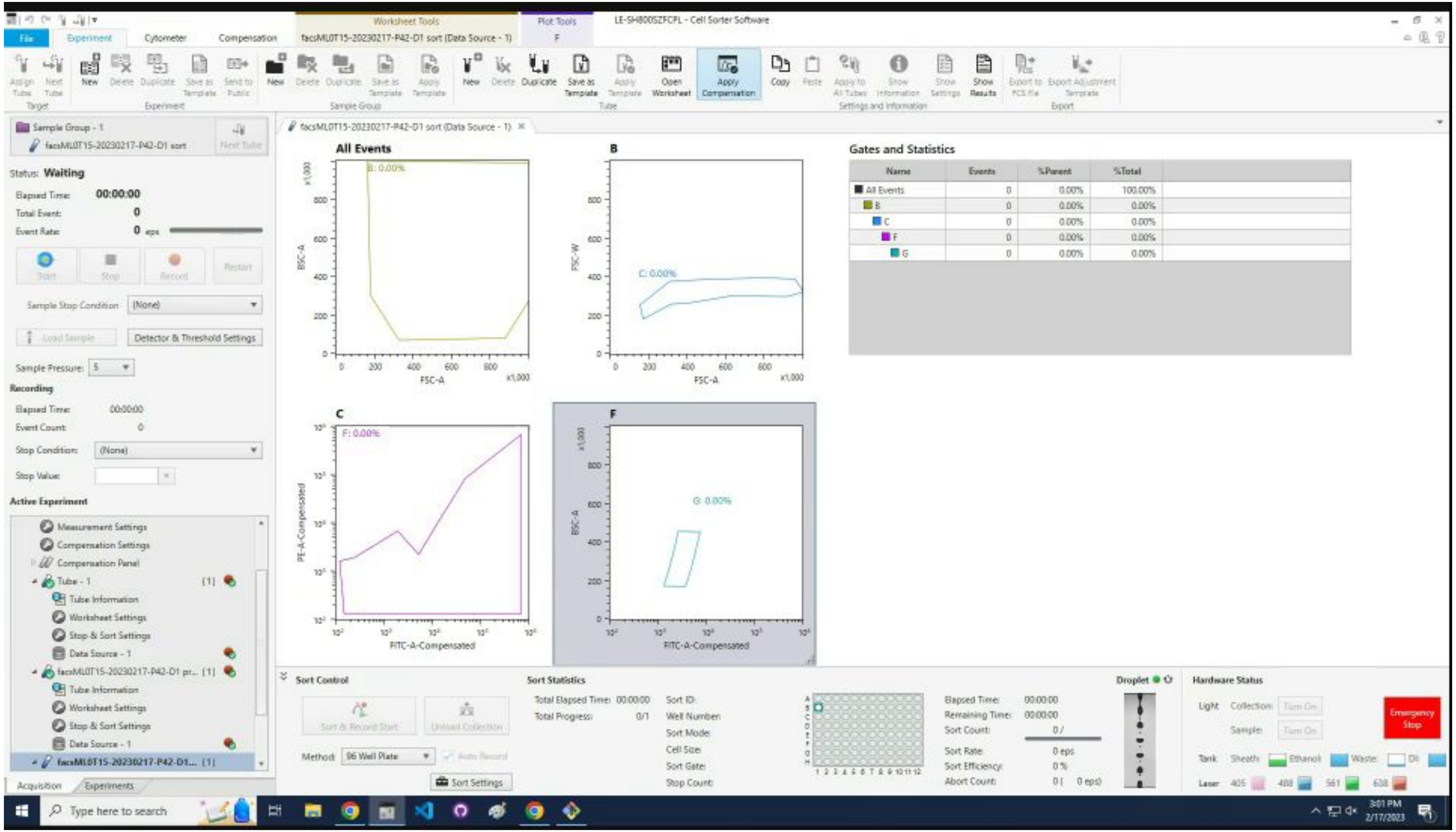

LE-SH800SZFCPL - Cell Sorter Software
File
Experiment
Cytometer
Compensation
Worksheet Tools
Plot Tools
Sample Group - 1
Status: Waiting
Elapsed Time: 00:00:00
Total Event: 0
Event Rate: 0
Start
Stop
Record
Restart
Sample Stop Condition
Detector & Threshold Settings
Sample Pressure: 5
Recording
Stop Condition:
Stop Value:
Active Experiment
Measurement Settings
Compensation Settings
Compensation Panel
Tube - 1
Tube Information
Worksheet Settings
Stop & Sort Settings
Data Source - 1
All Events
B
C
F
FSC-A
BSC-A
FSC-W
FITC-A-Compensated
PE-A-Compensated
Gates and Statistics
Name
Events
%Parent
%Total
Sort Control
Sort Statistics
Method: 96 Well Plate
Sort Settings
Droplet
Hardware Status
Emergency Stop
Acquisition
Experiments
Type here to search

Wait ~20 seconds for cells to appear.

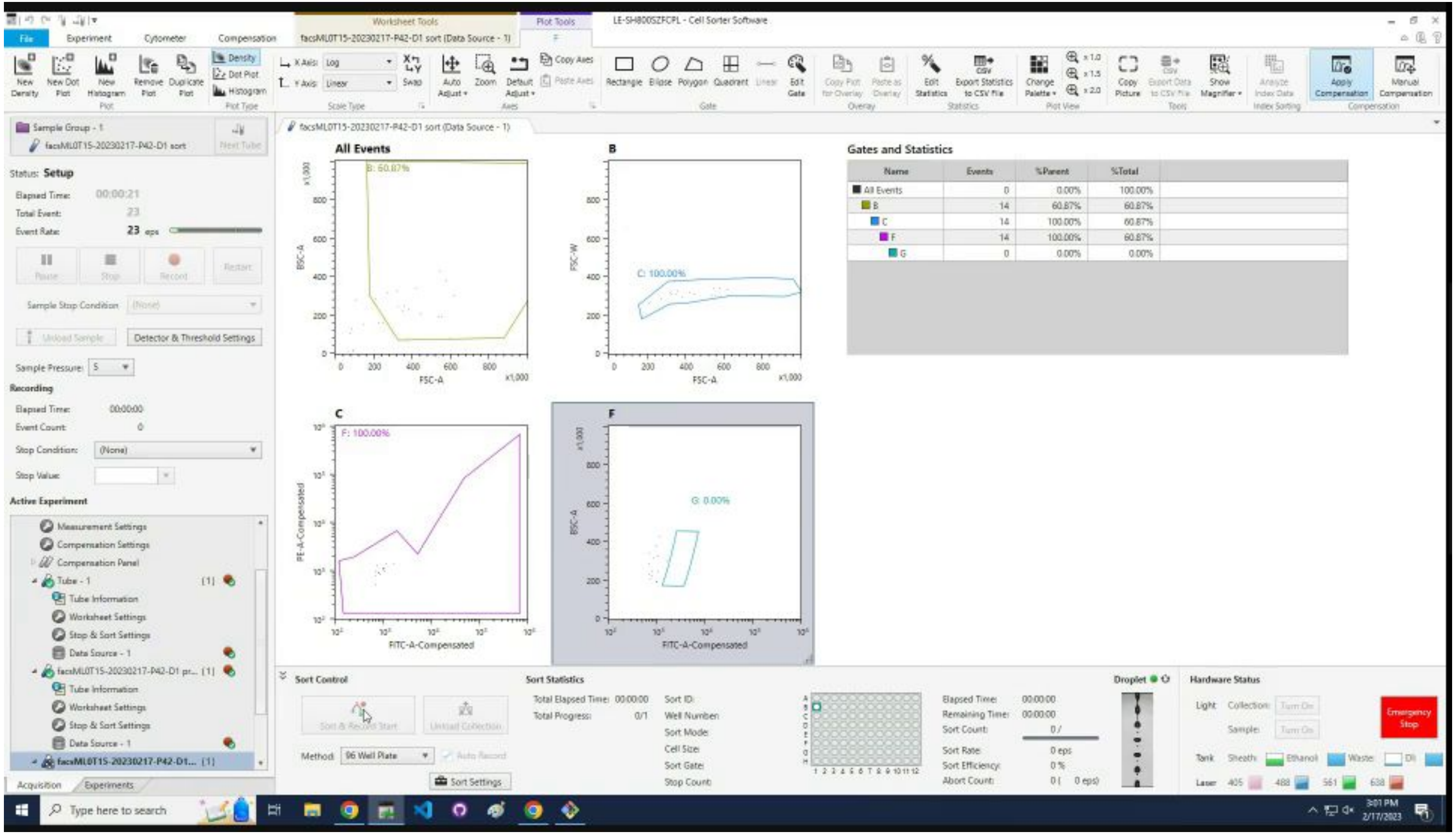

Click `Sort & Record Start.`

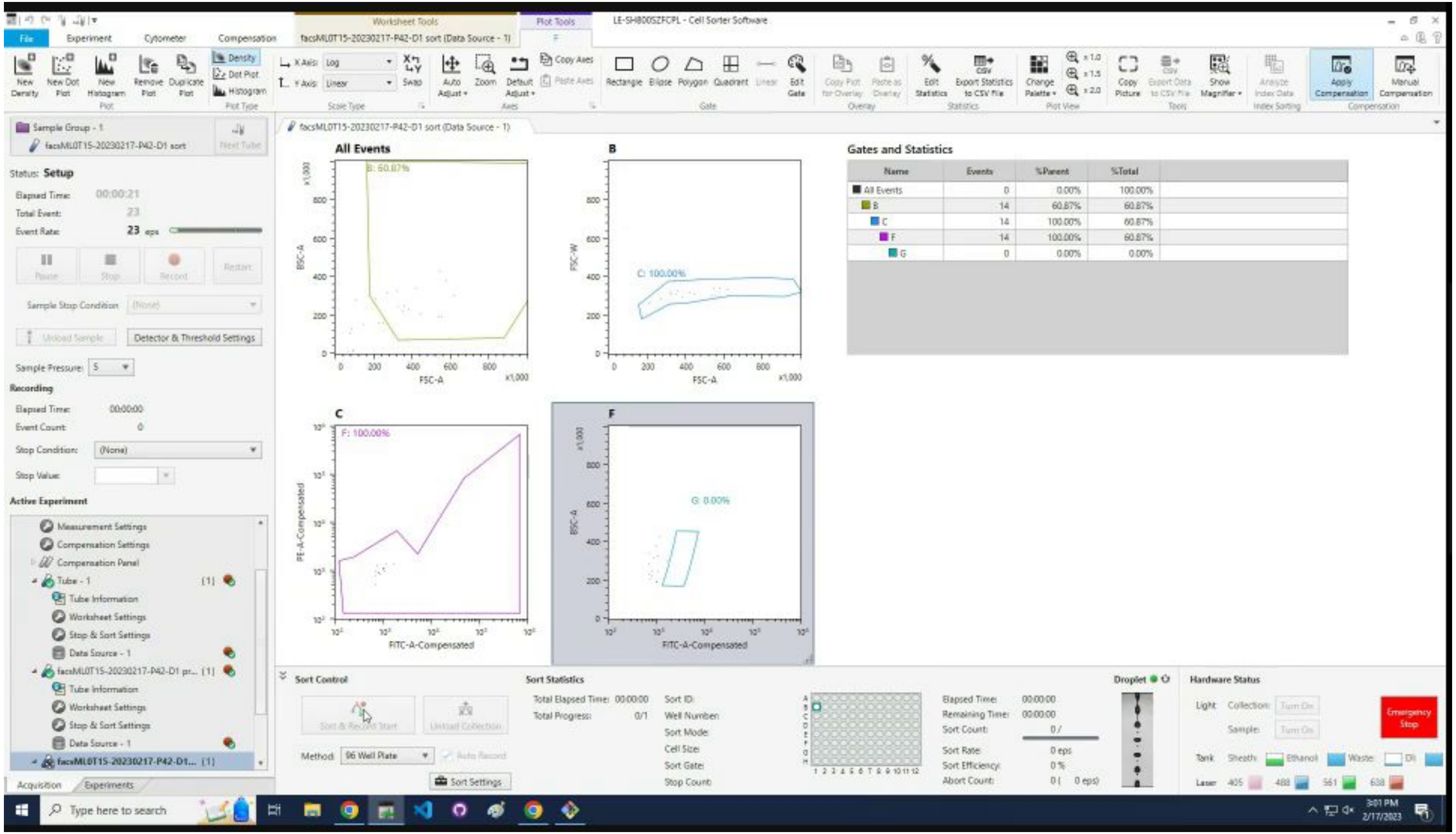

Wait for the sort to finish.

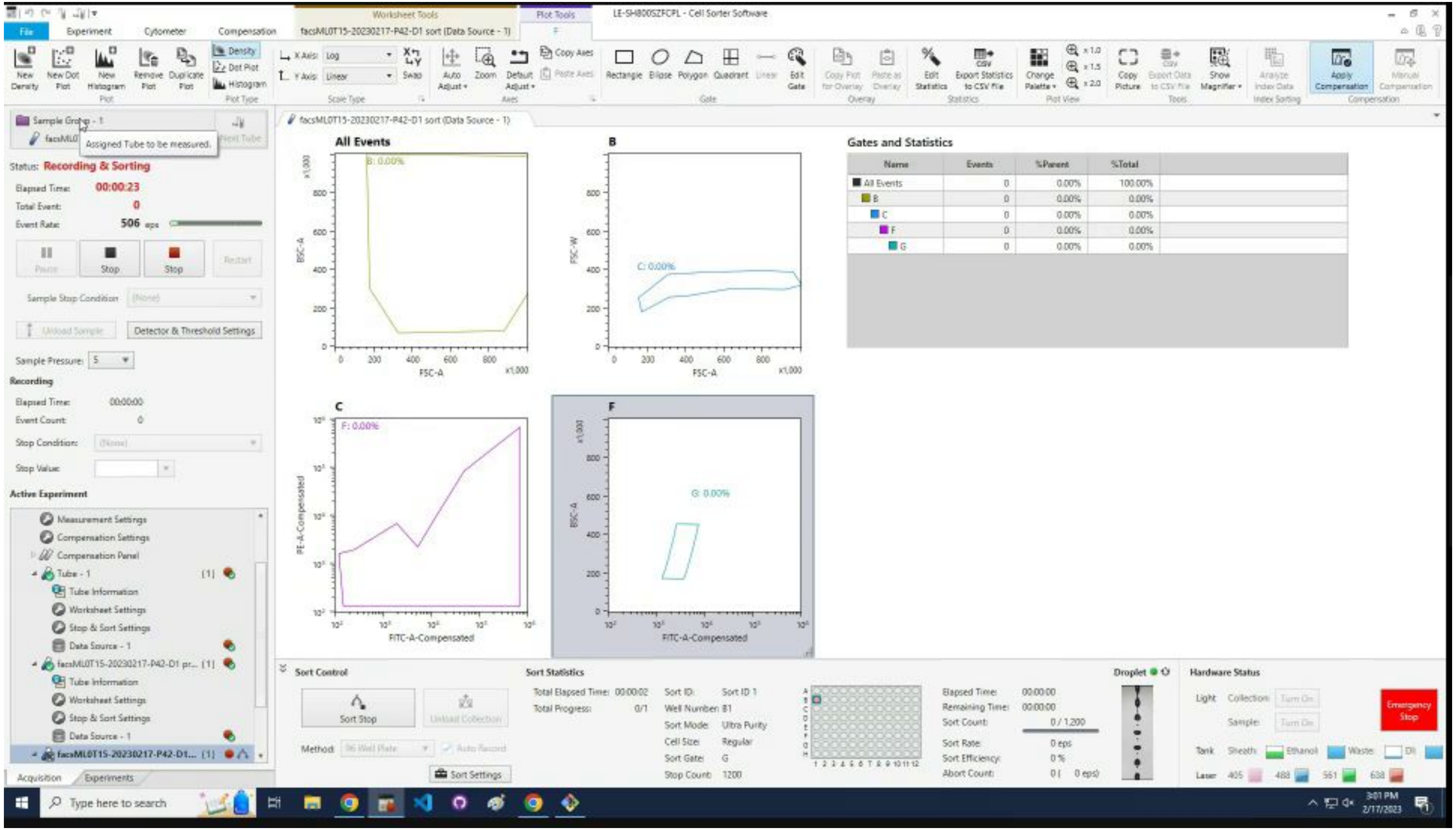

Click `Finish`.

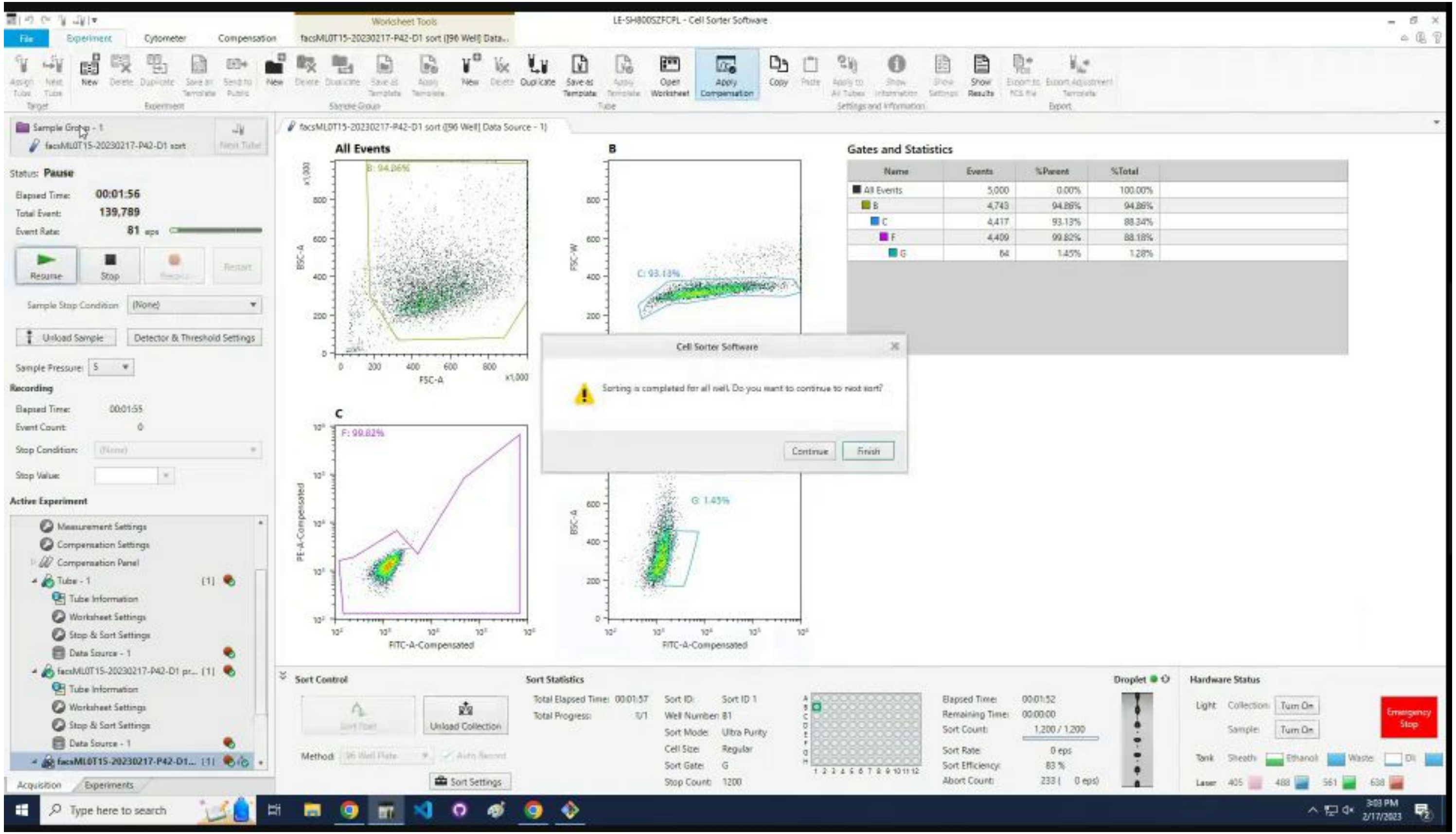

Click the X to close the tube tab.

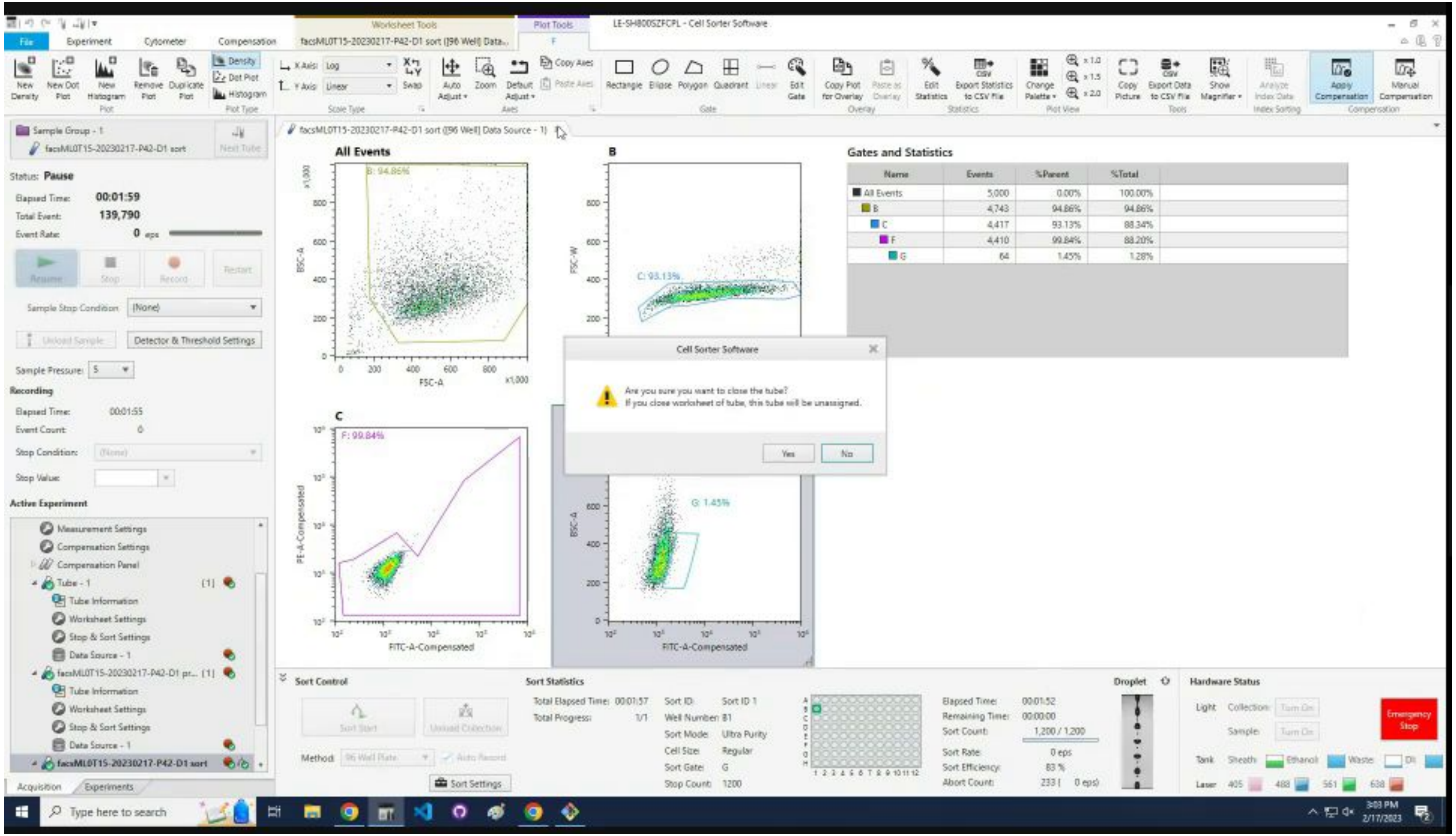

Click `Yes` to confirm closing the tube tab.

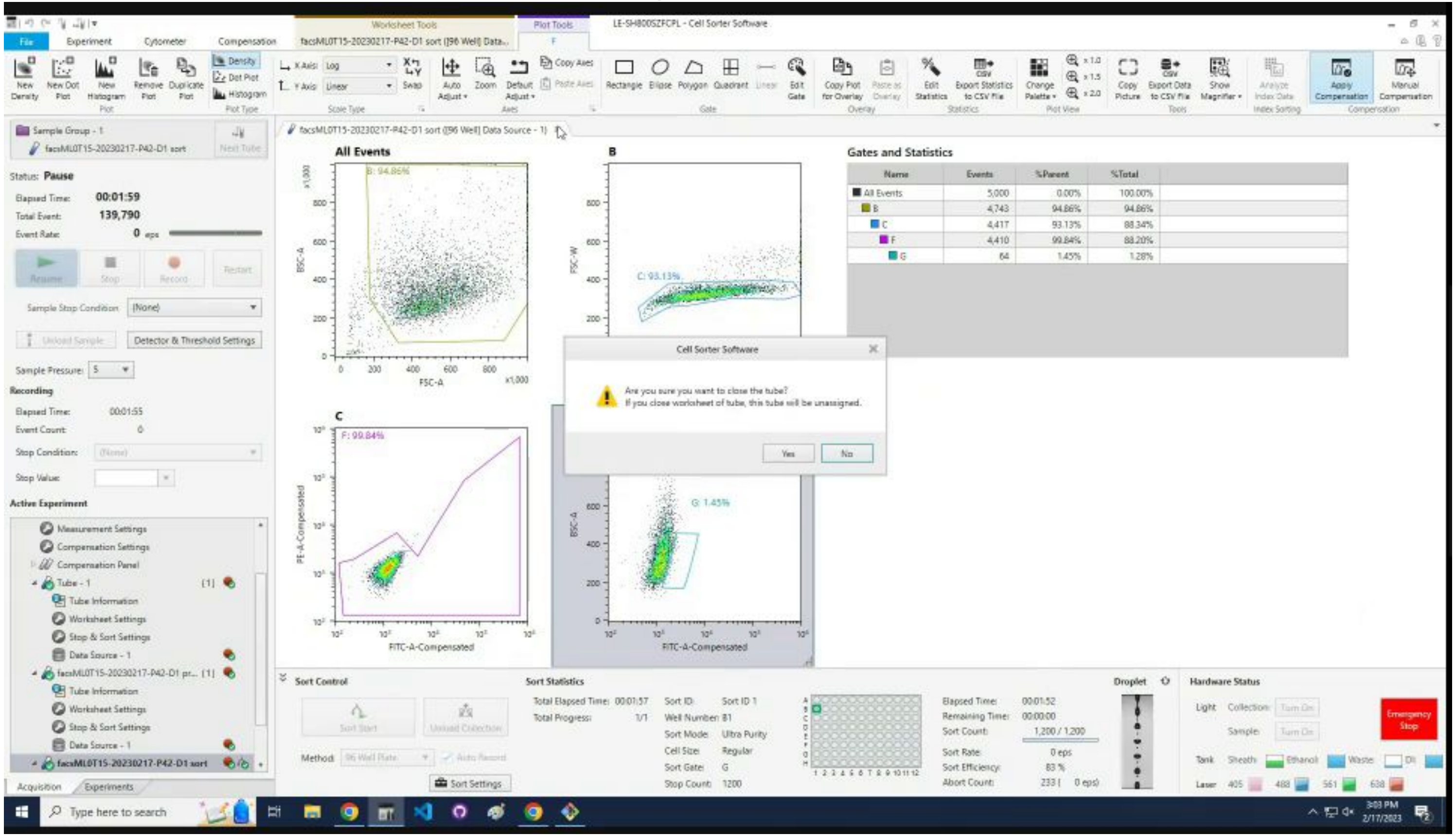

Wait for the Sony to refresh the GUI after `Probe Wash`.

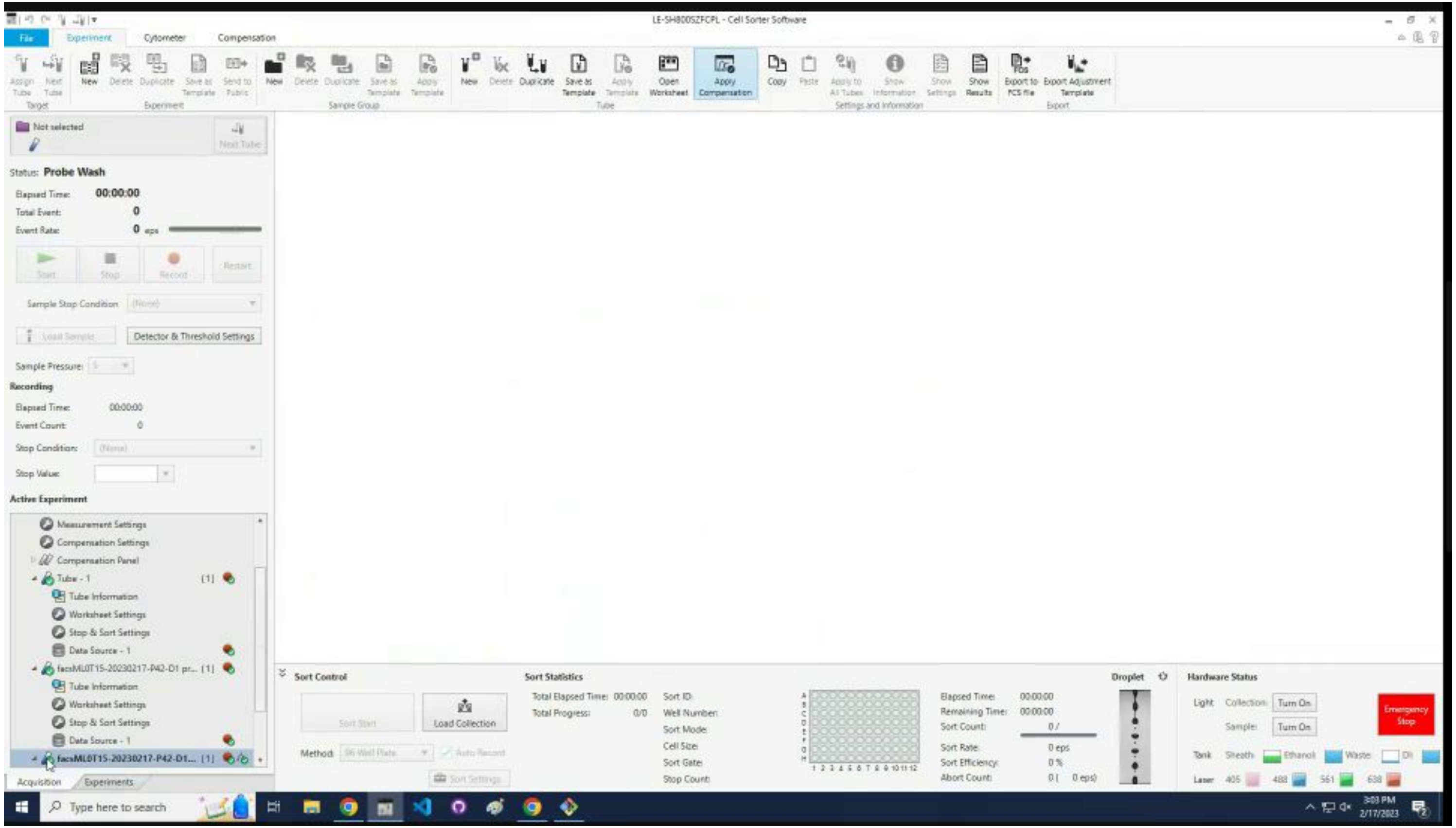

Click on the finished sample tube.

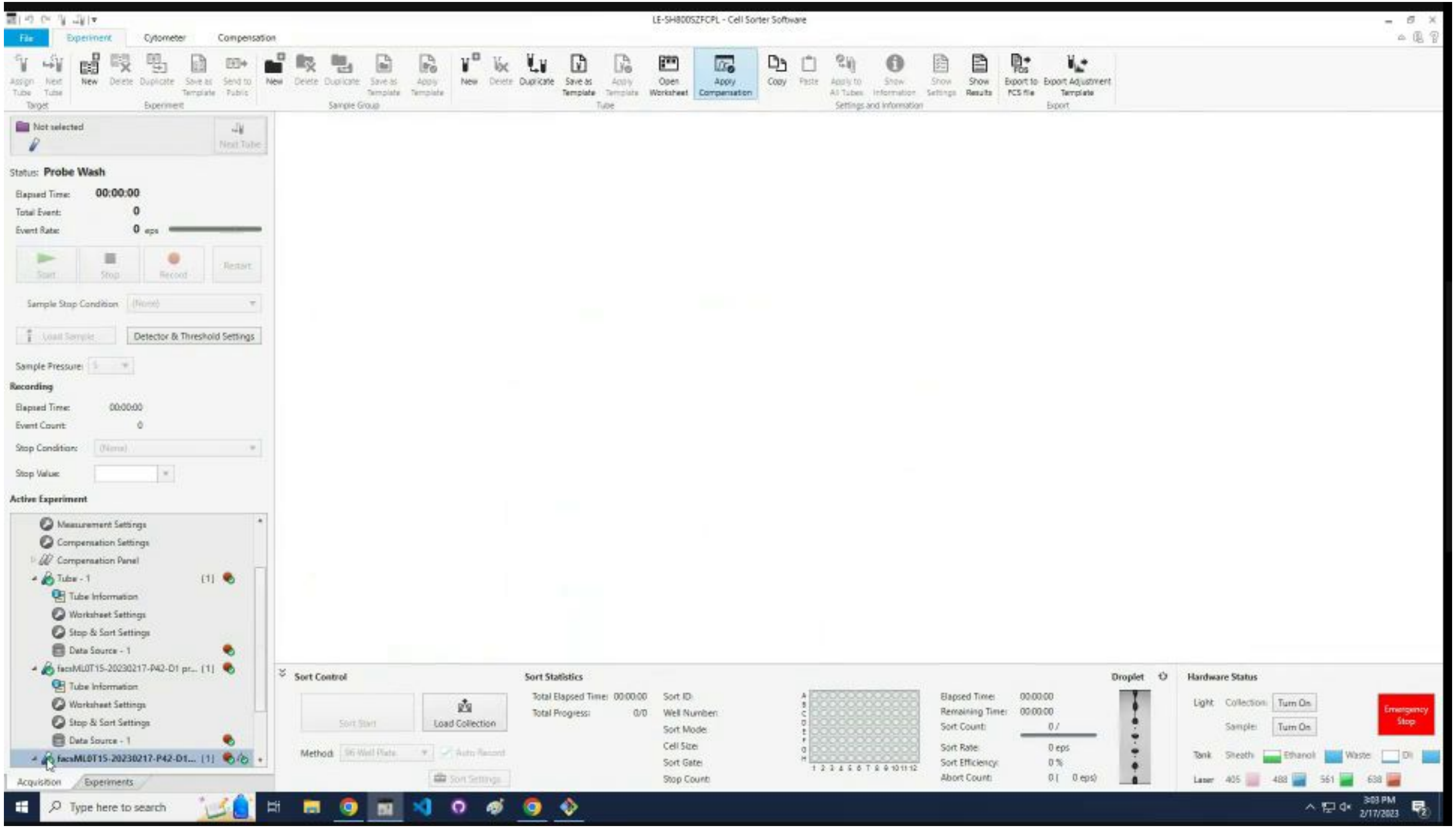

Click the `Duplicate` icon.

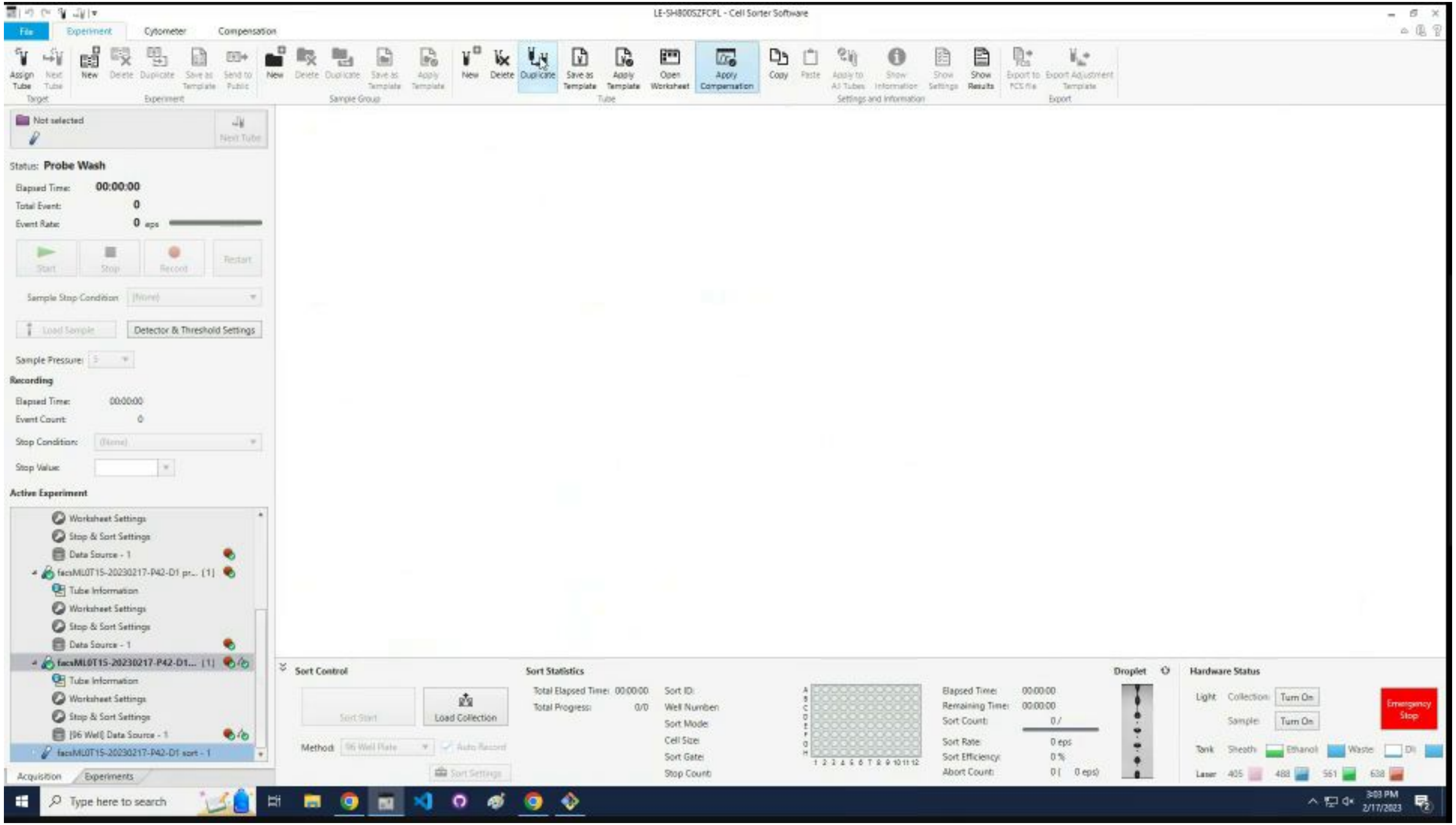

Click the `Assign Tube` icon.

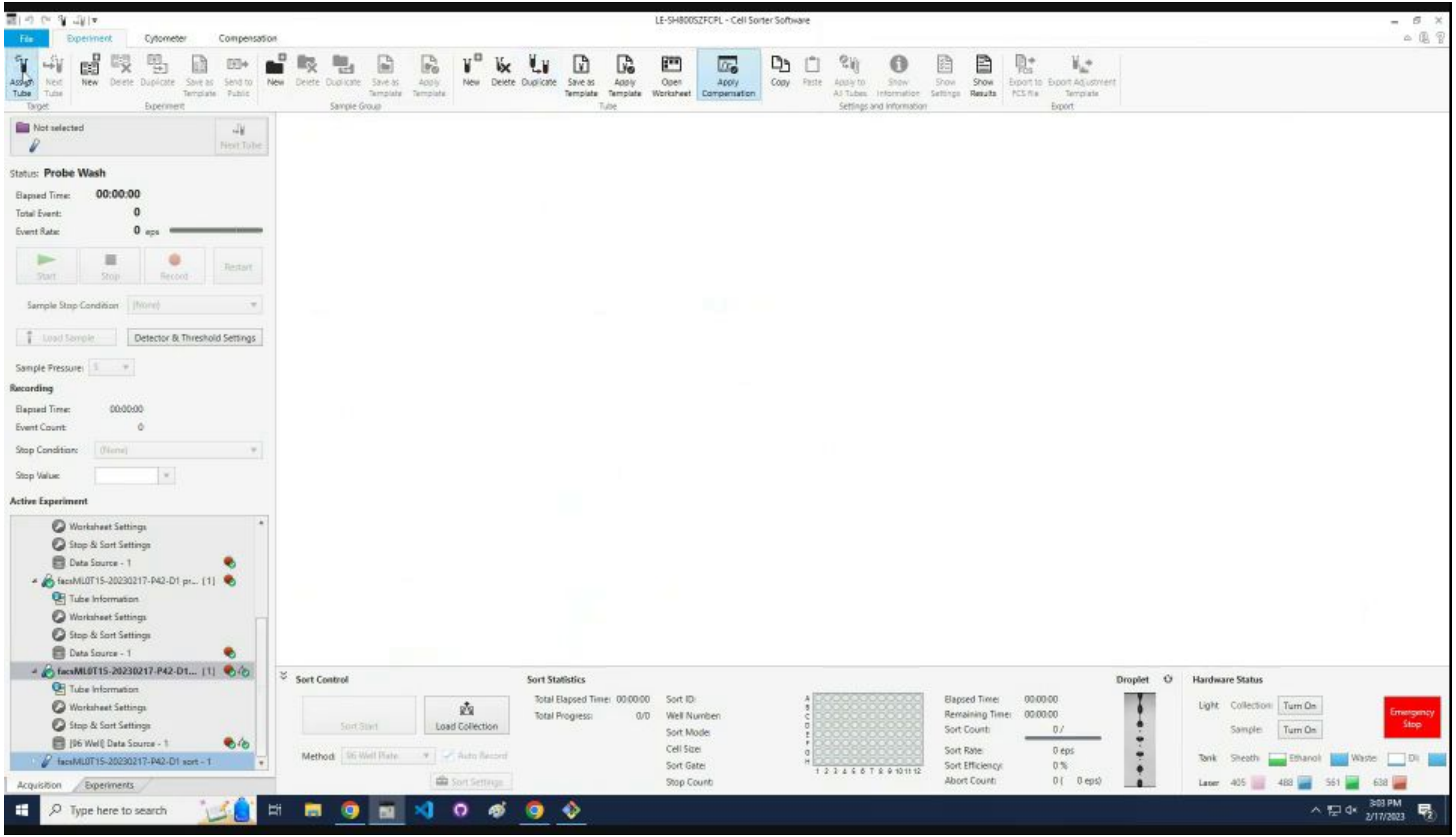

Repeat Iterate through samples section for more samples.

Shutdown

Answer `y/n` to close solenoid. (`No`, if sorting again.)

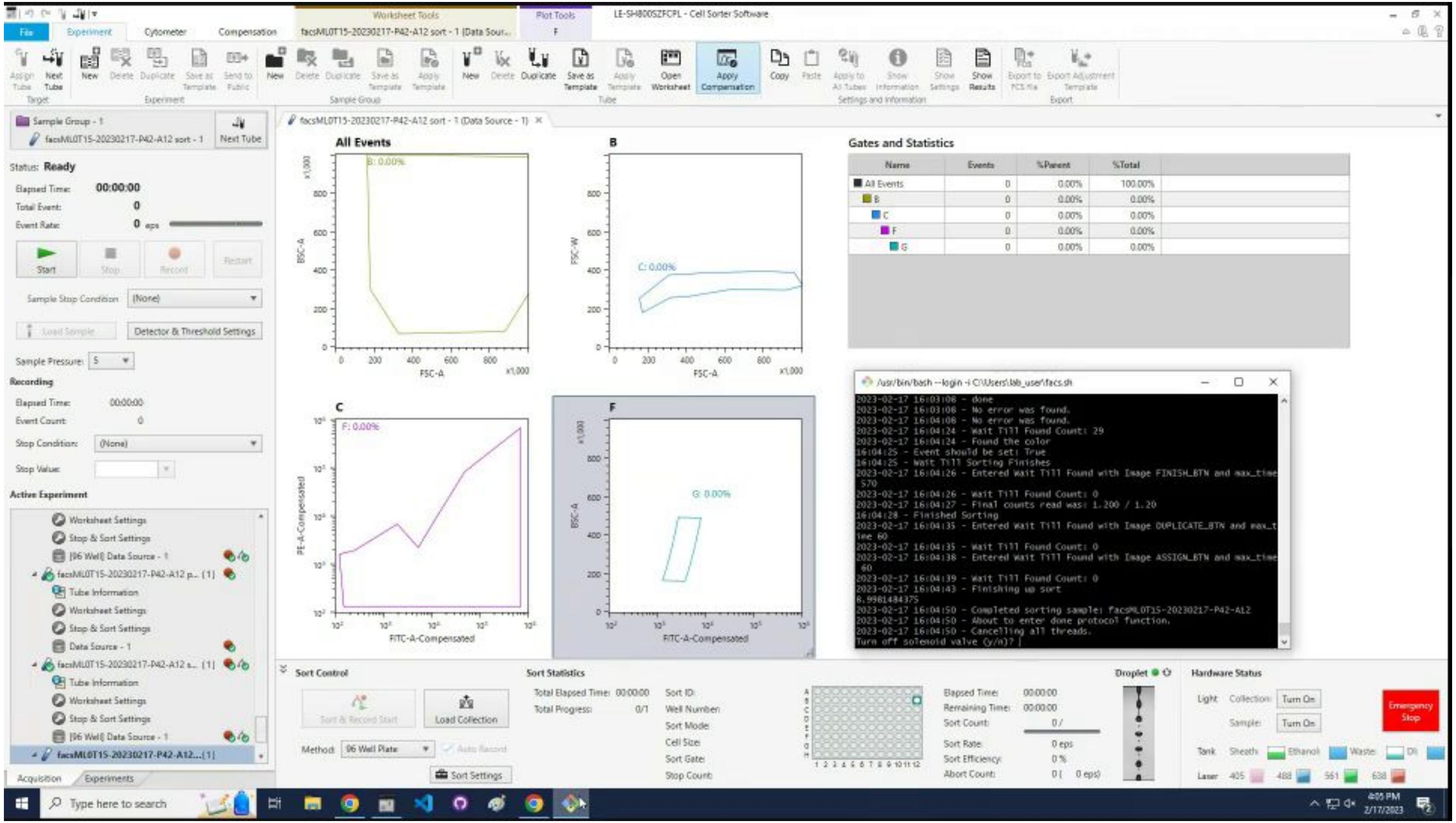

An active blue tube remains for the next sorting run.

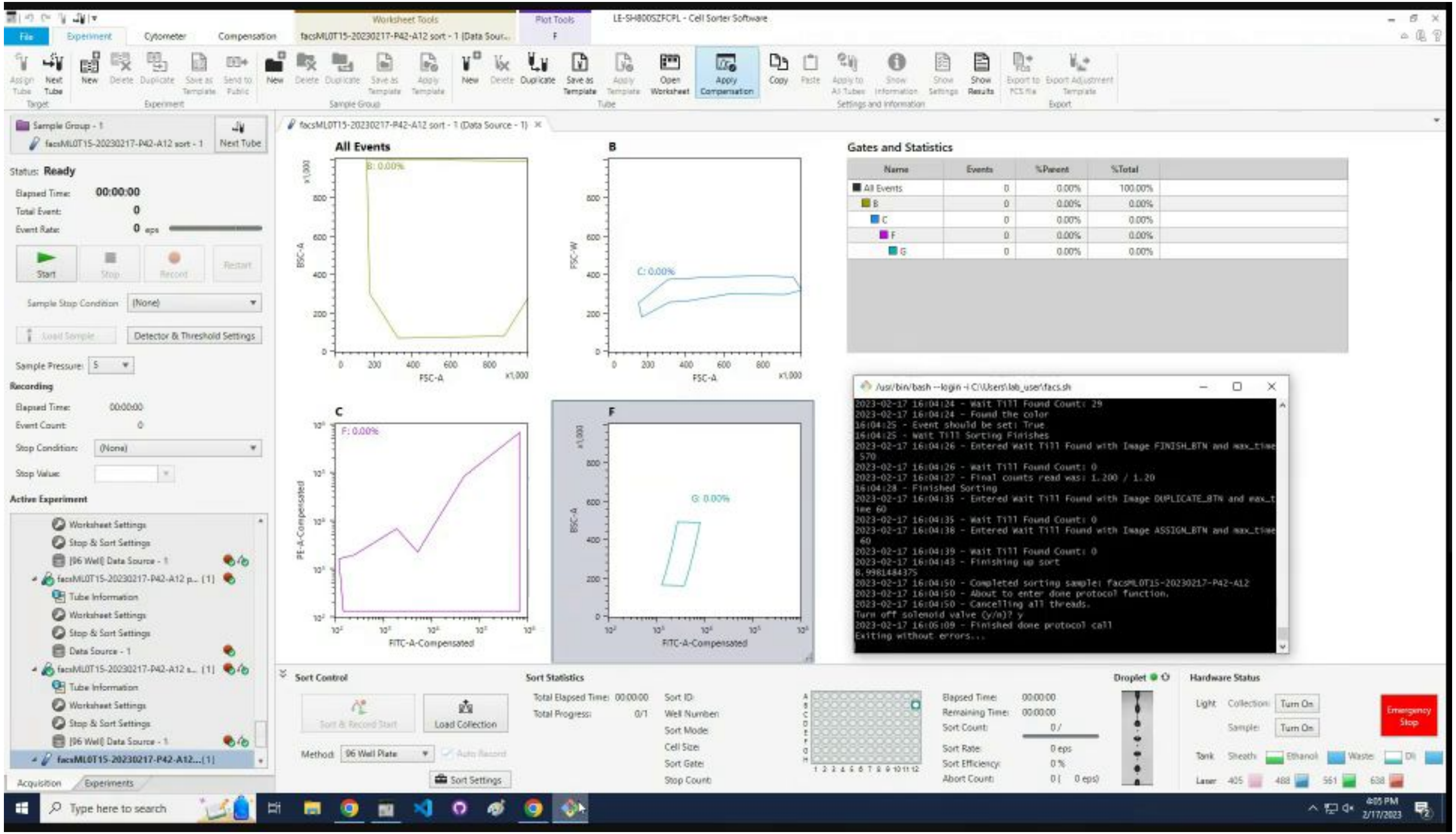

Data Scraping

Start the automated data scraping by clicking `scraper`.

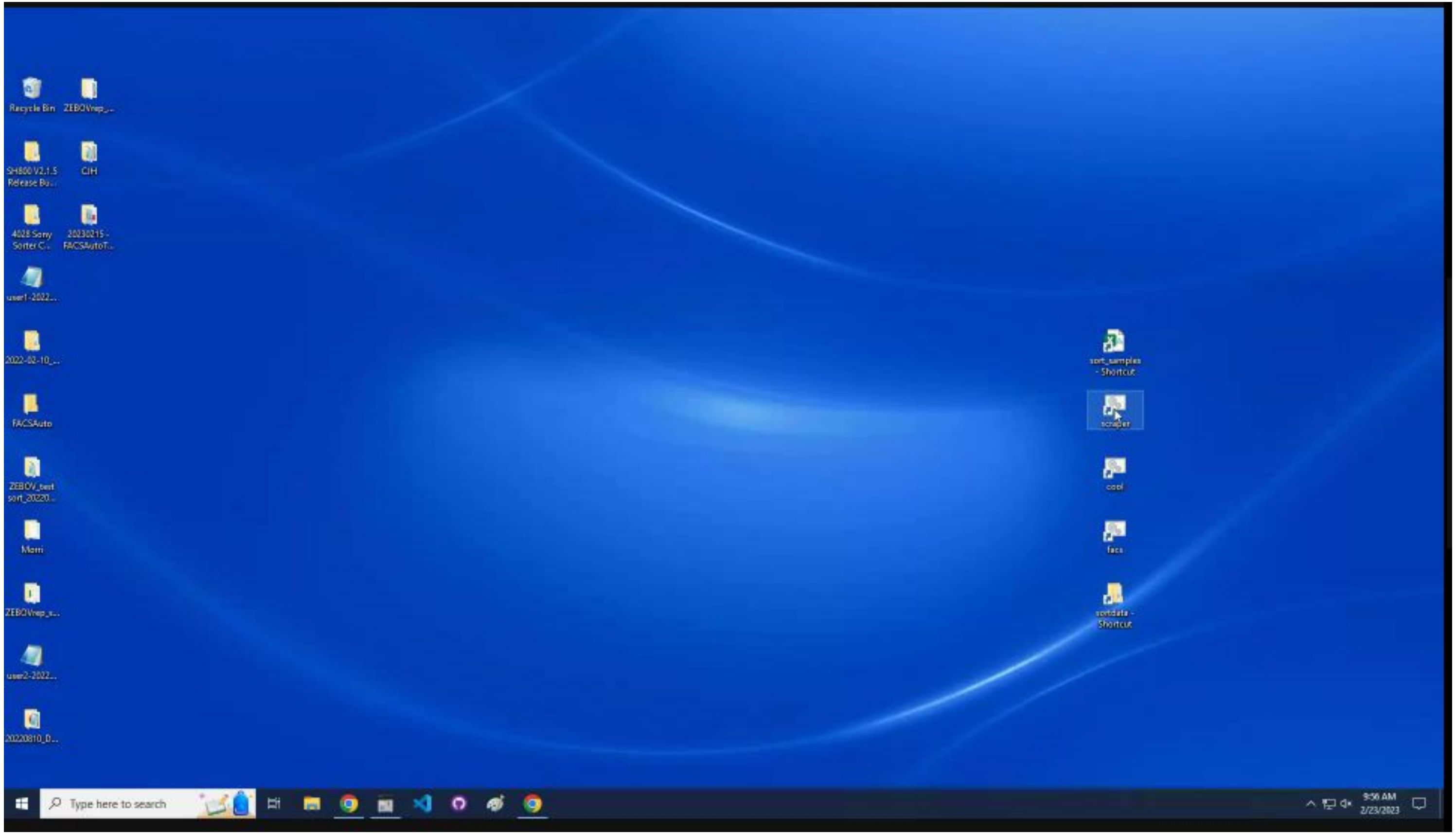

Enter `y/n` to export data from the Cell Sorter software.

If `yes`, skip to export.

If `no`, proceed to next slide.

Enter `n` on startup, and skip to plotting.

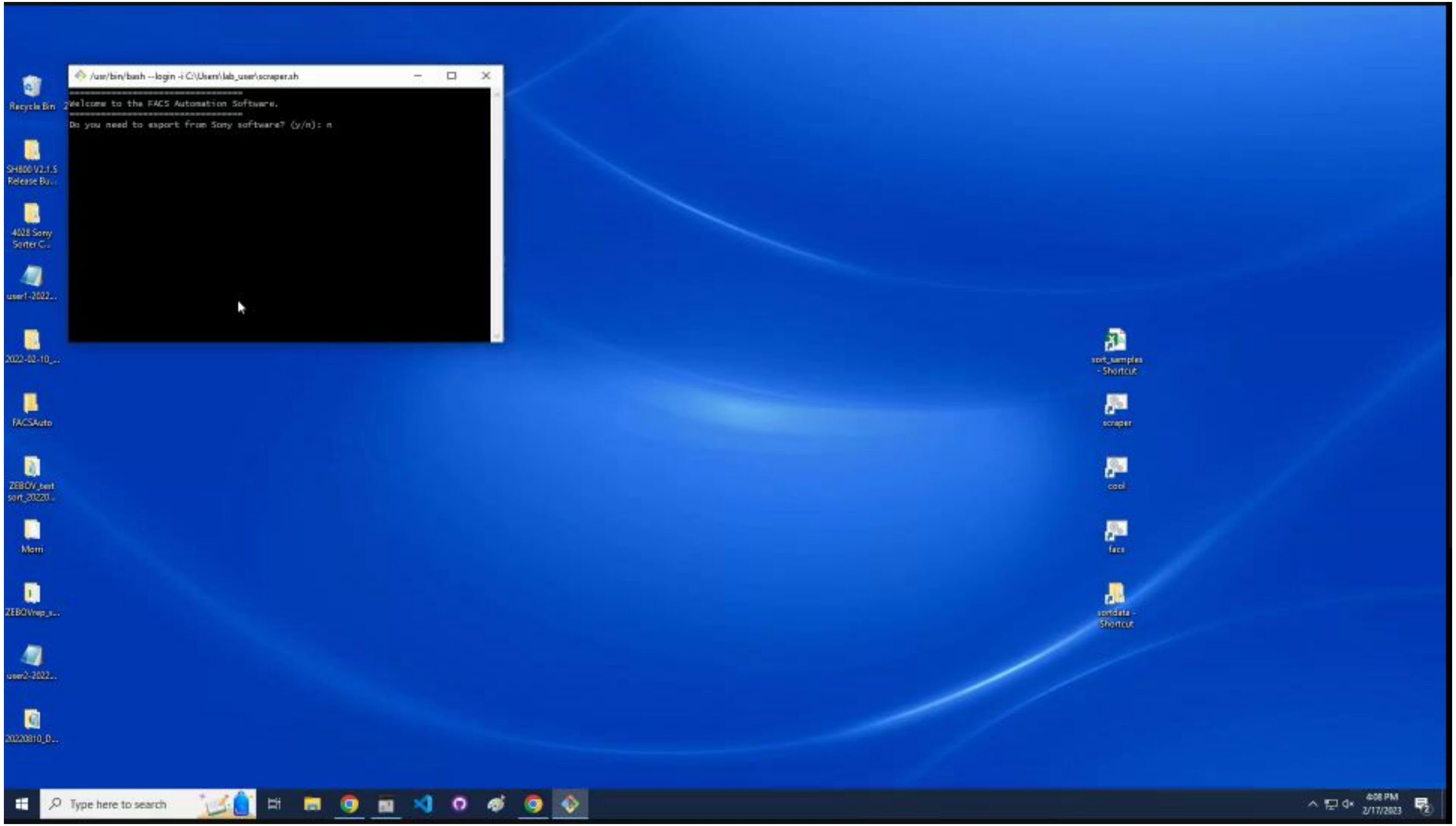

Align the experiment to the top.

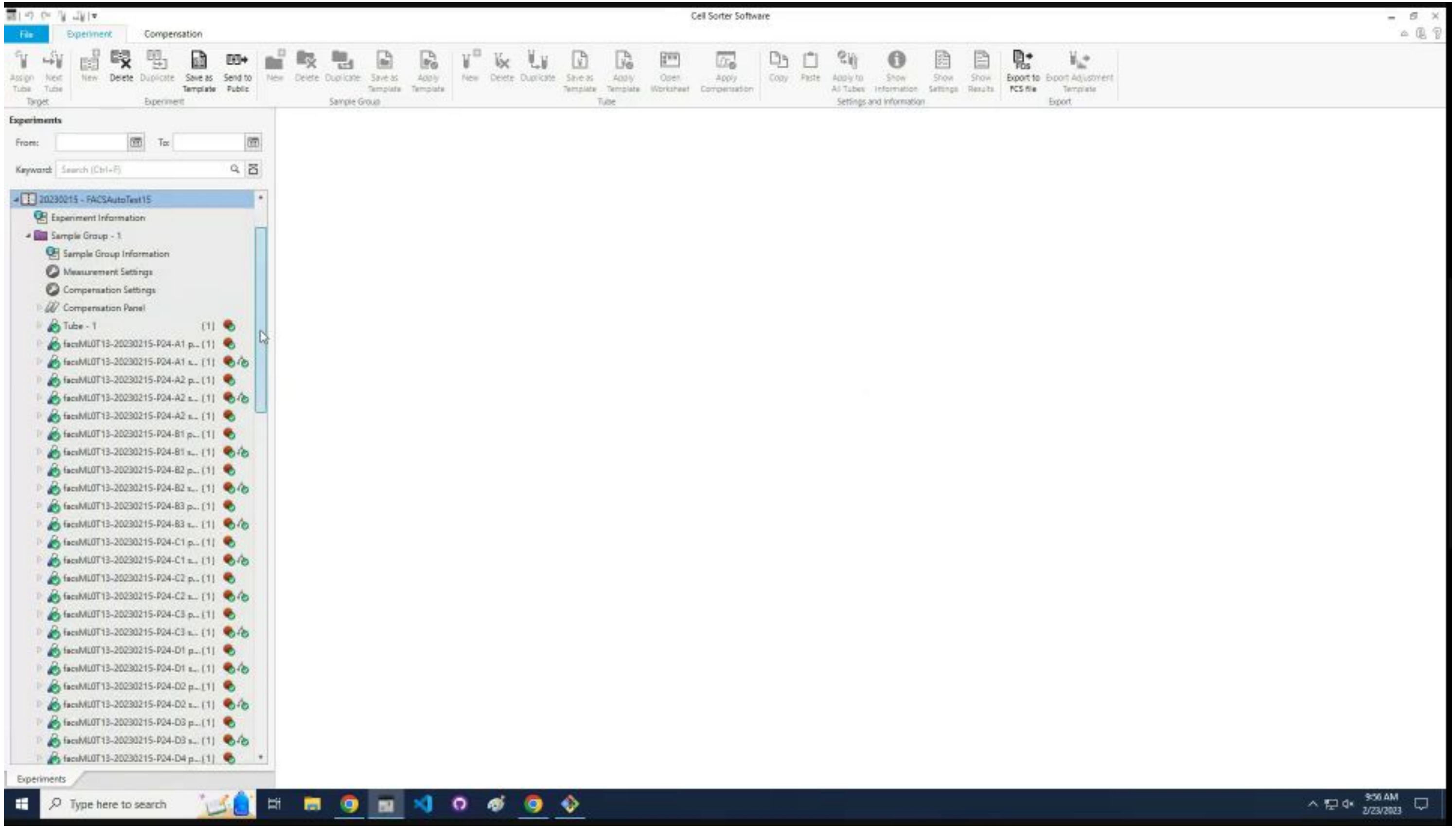

Enter `y` to start the exports.

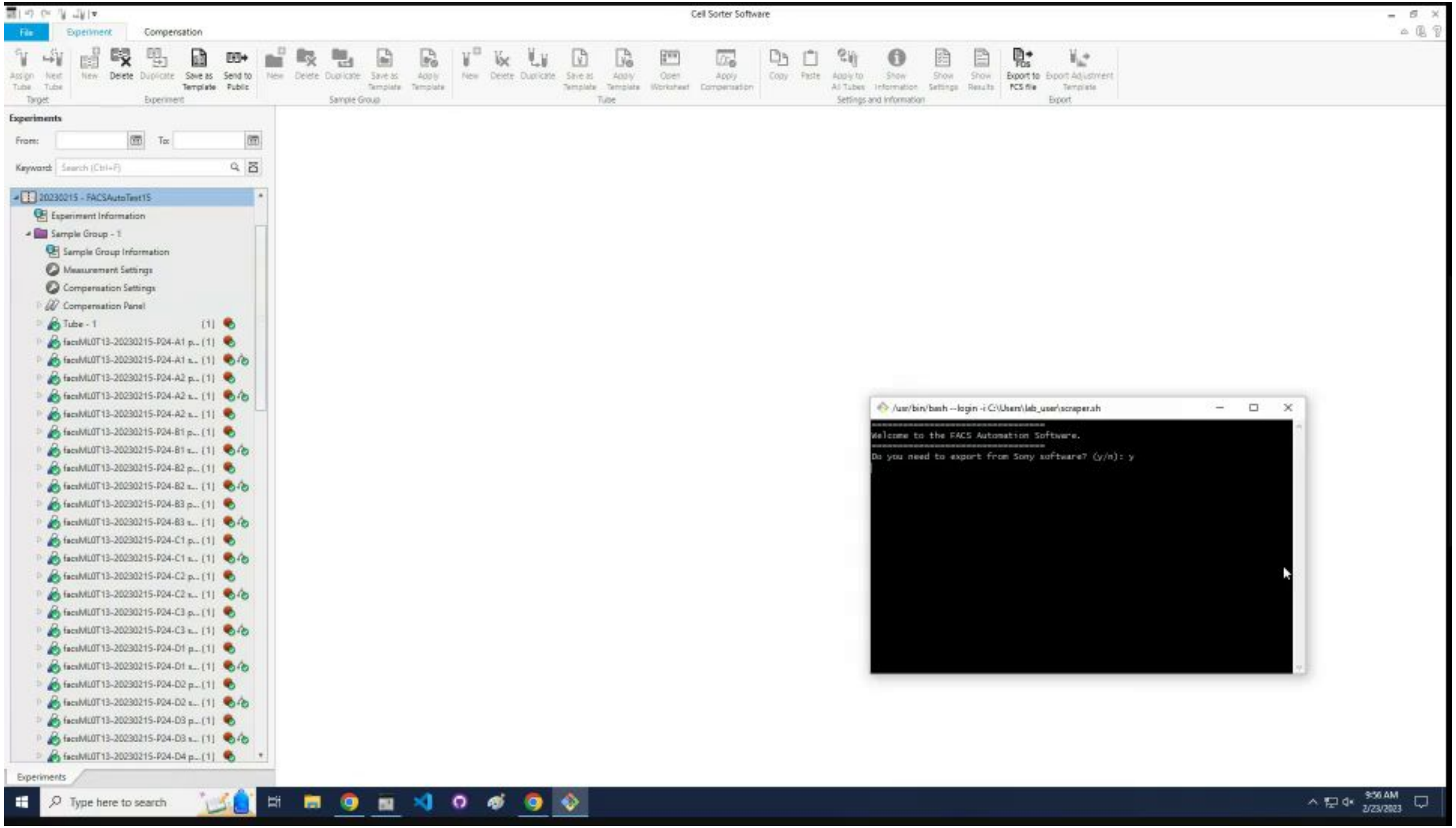

The program finds the sorted samples.

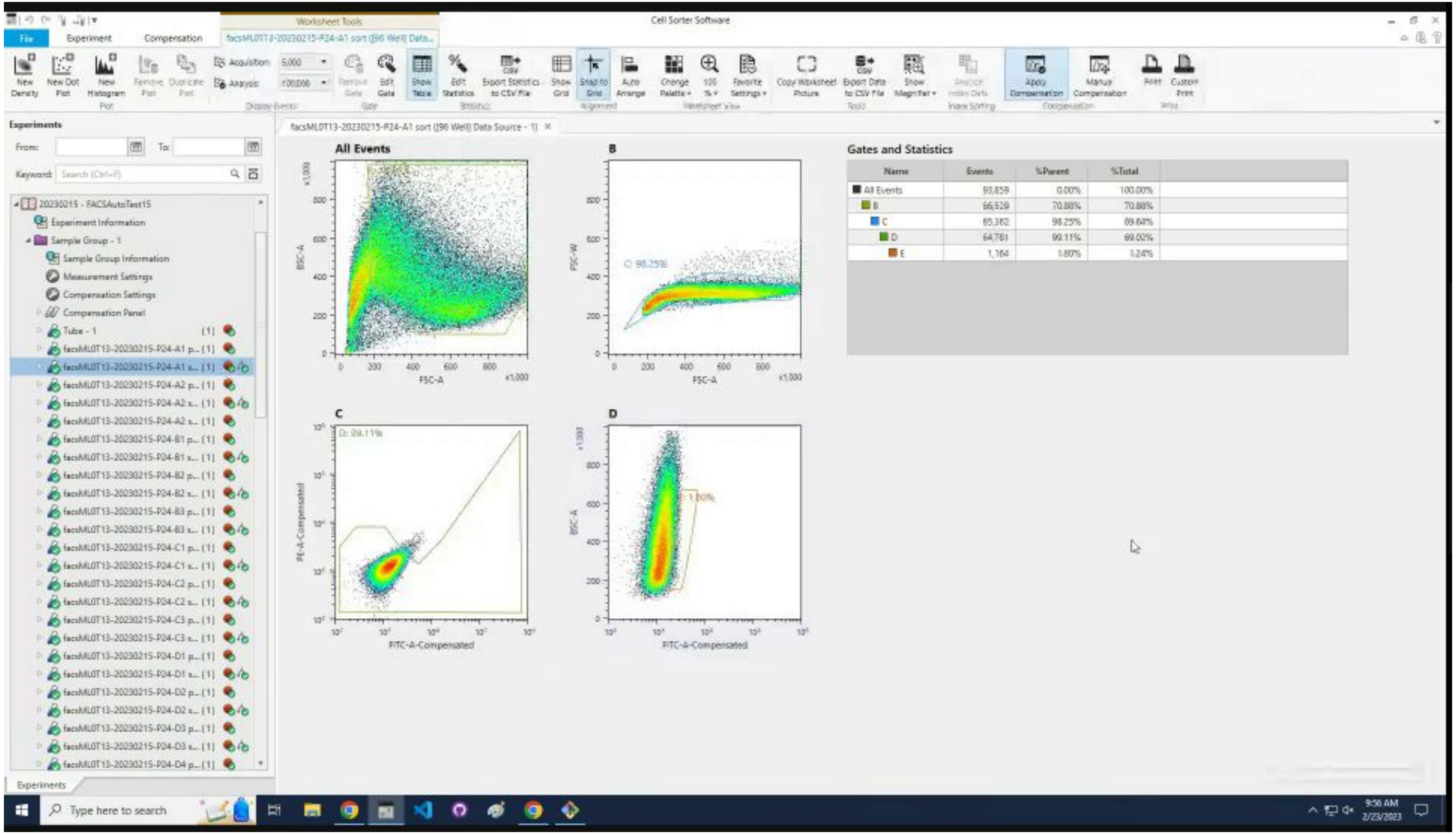

The filename is updated and the data is exported.

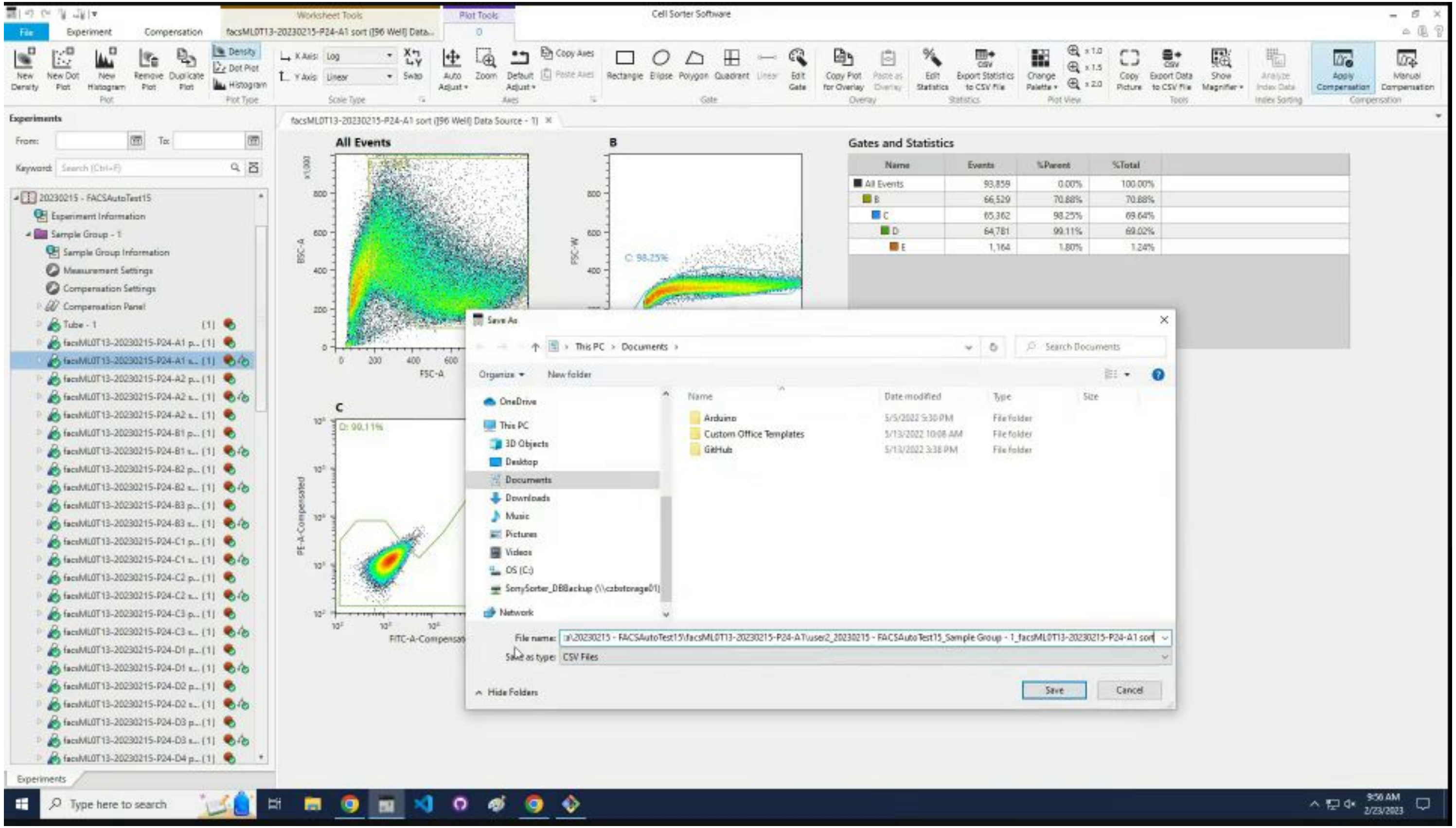

The program scrolls through the full list of experiments.

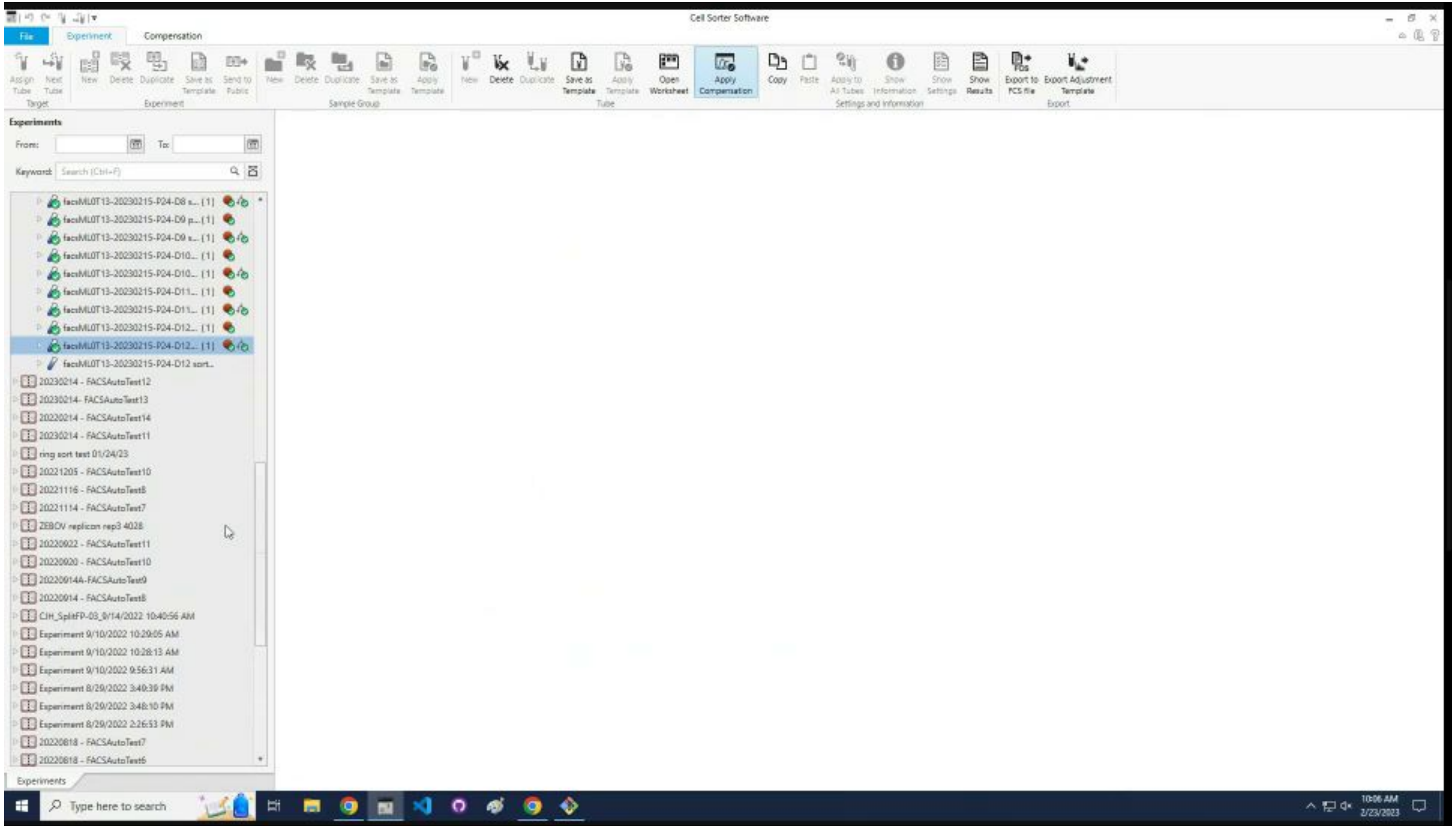

The program asks for the folder with the data.

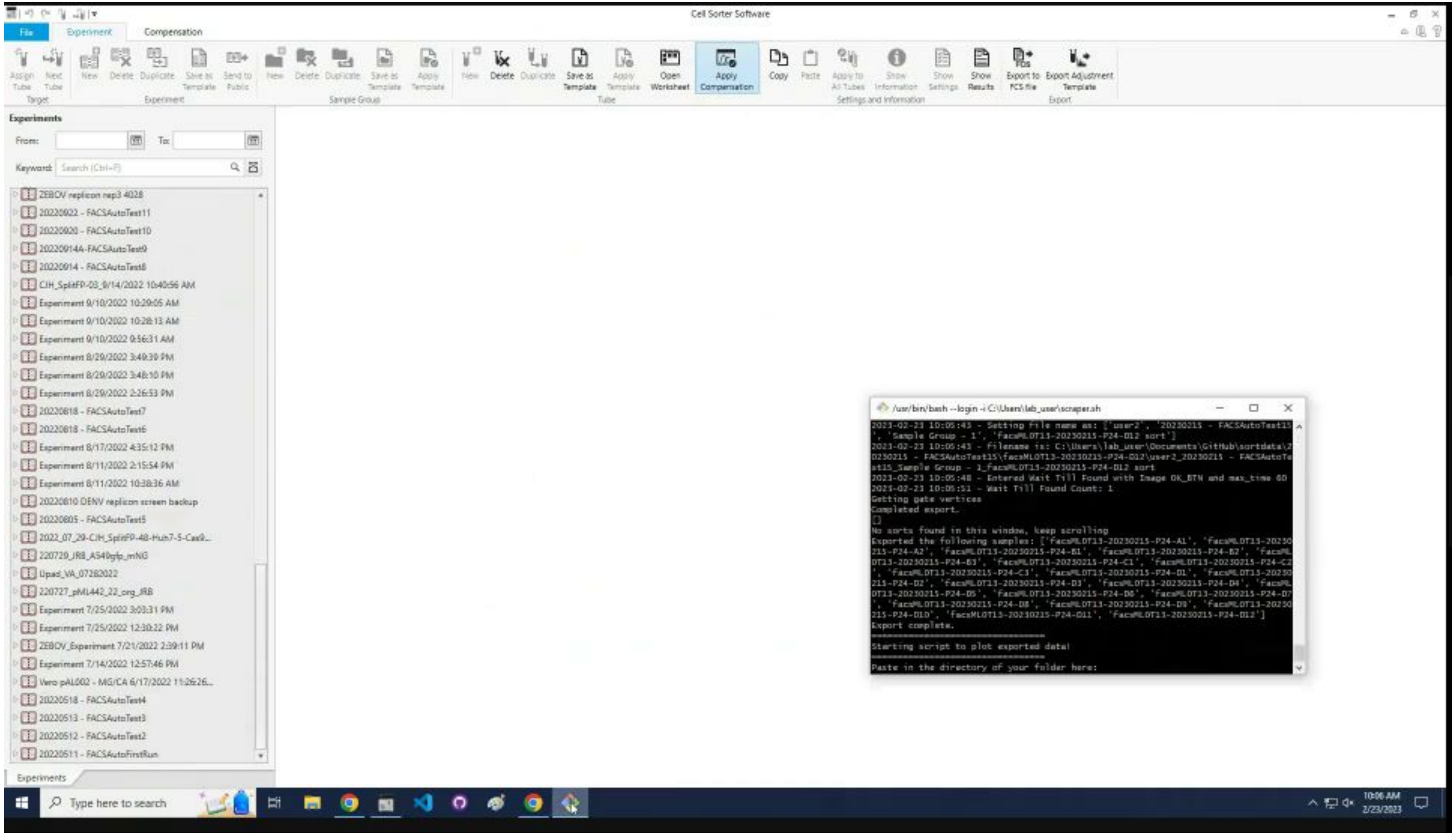

Navigate to the `sortdata` folder.

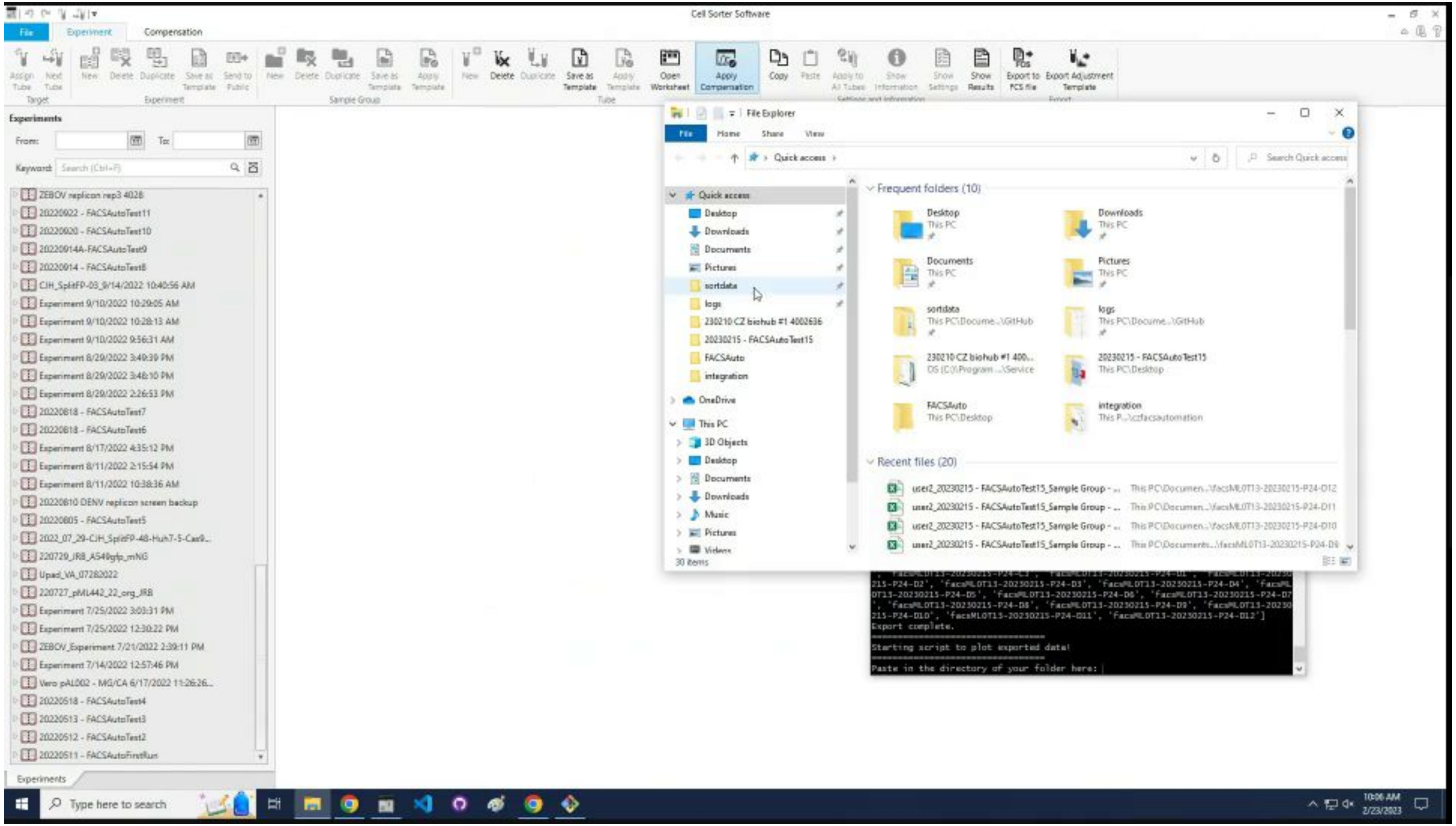

Copy the file path for the folder containing the data.

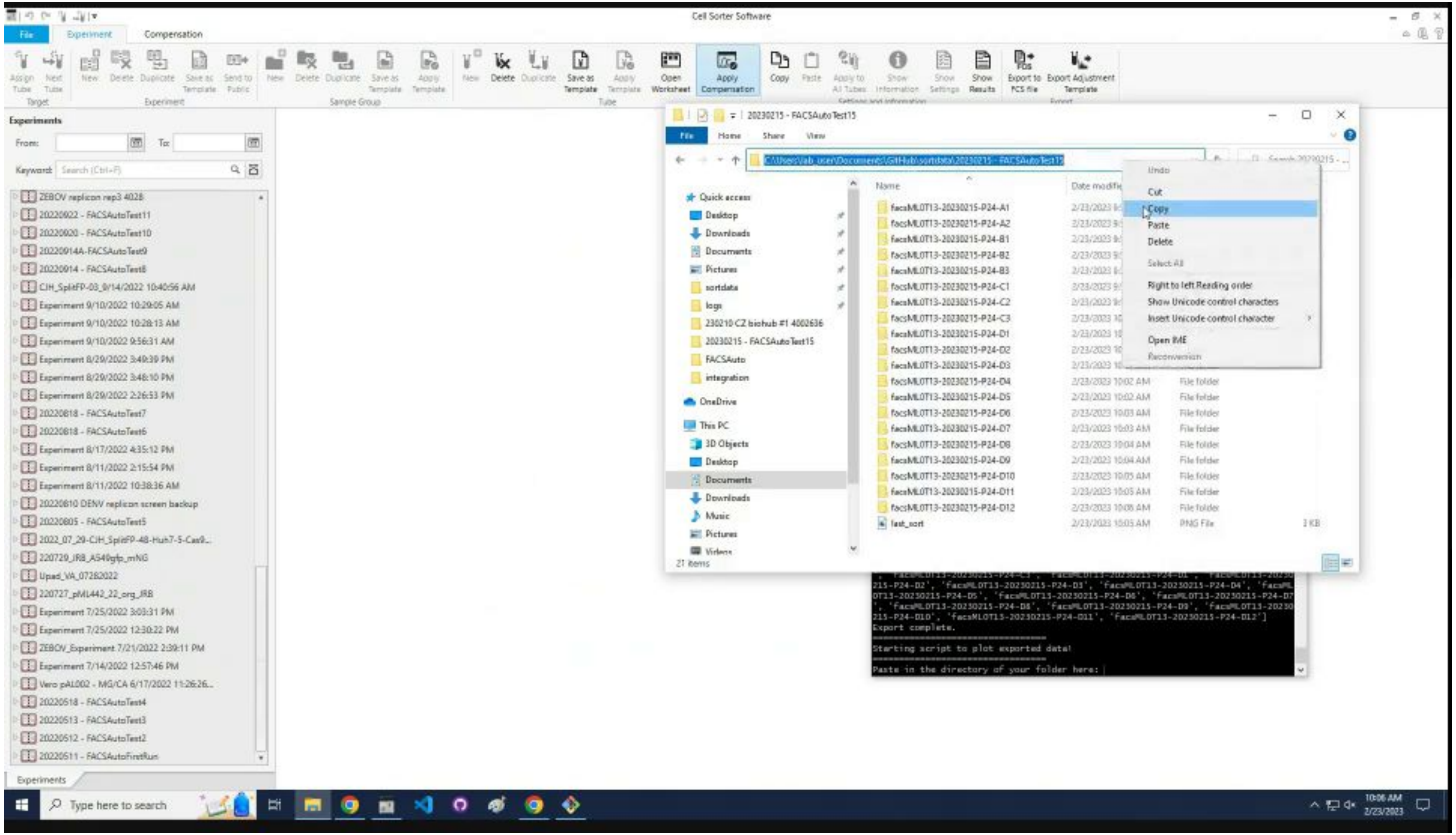

Use `Right Click + Paste + Enter` to paste it.

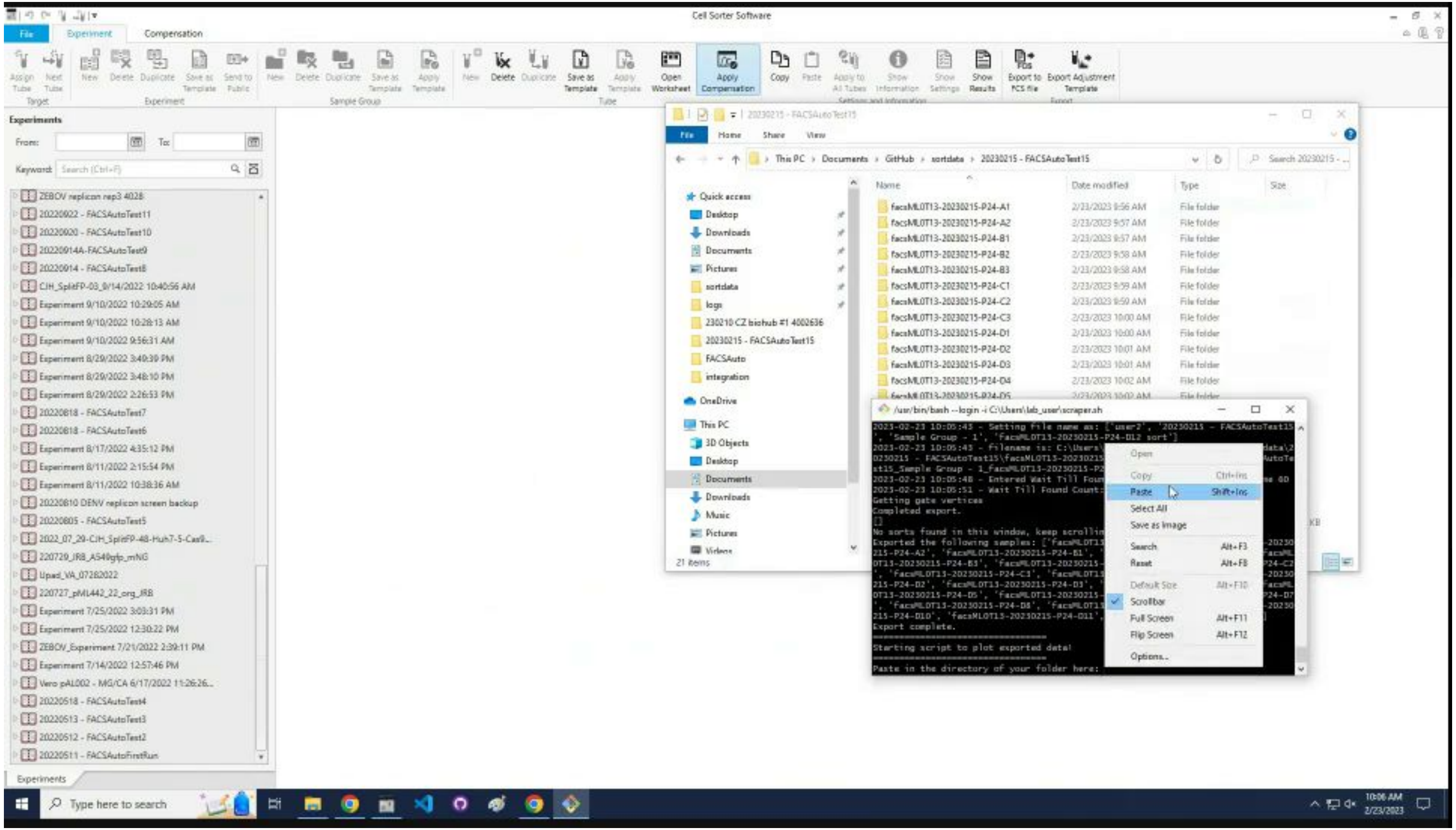

Select which data to plot, `Enter` for all of it.

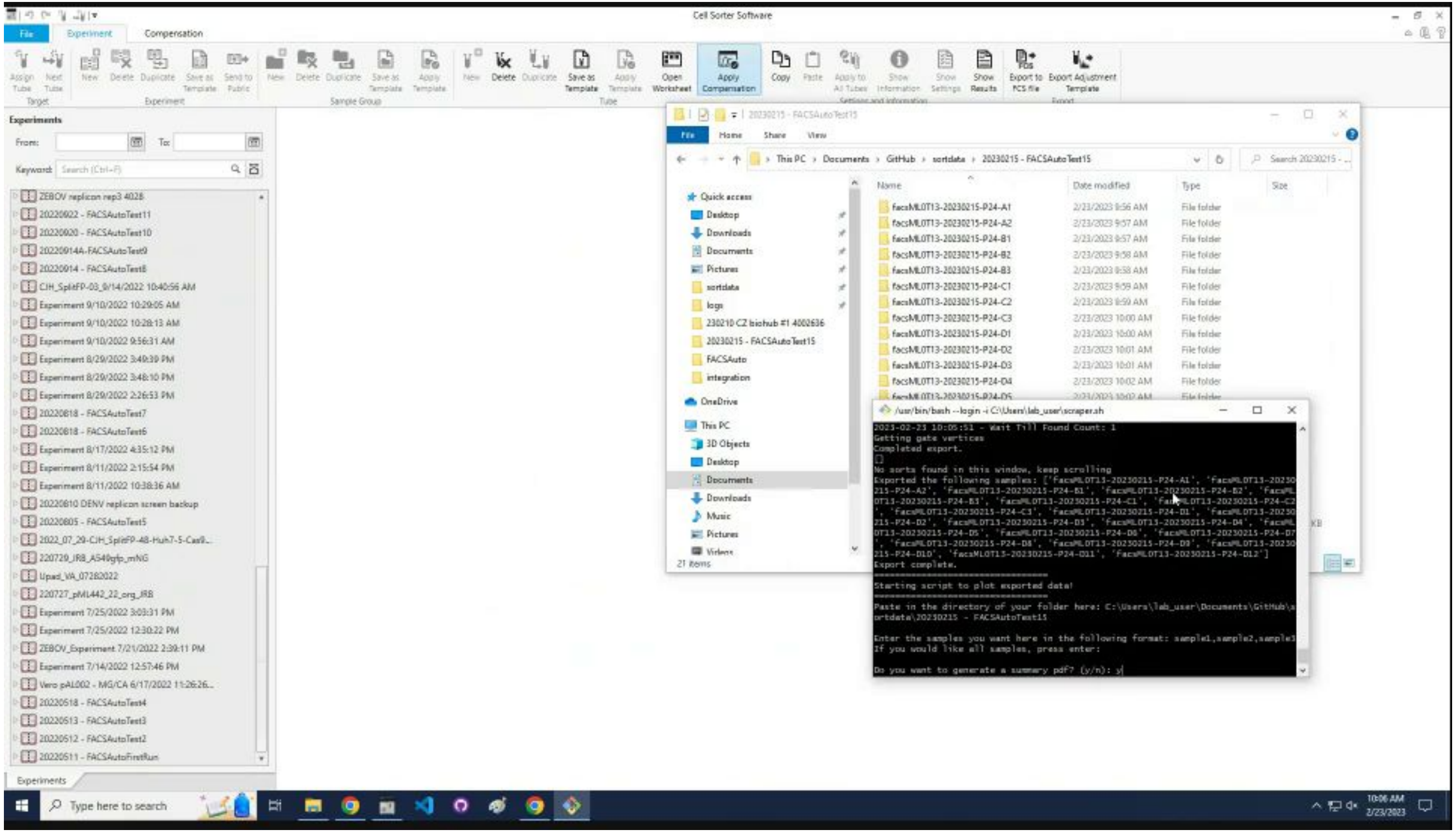

Select `y/n` to generate a summary PDF.

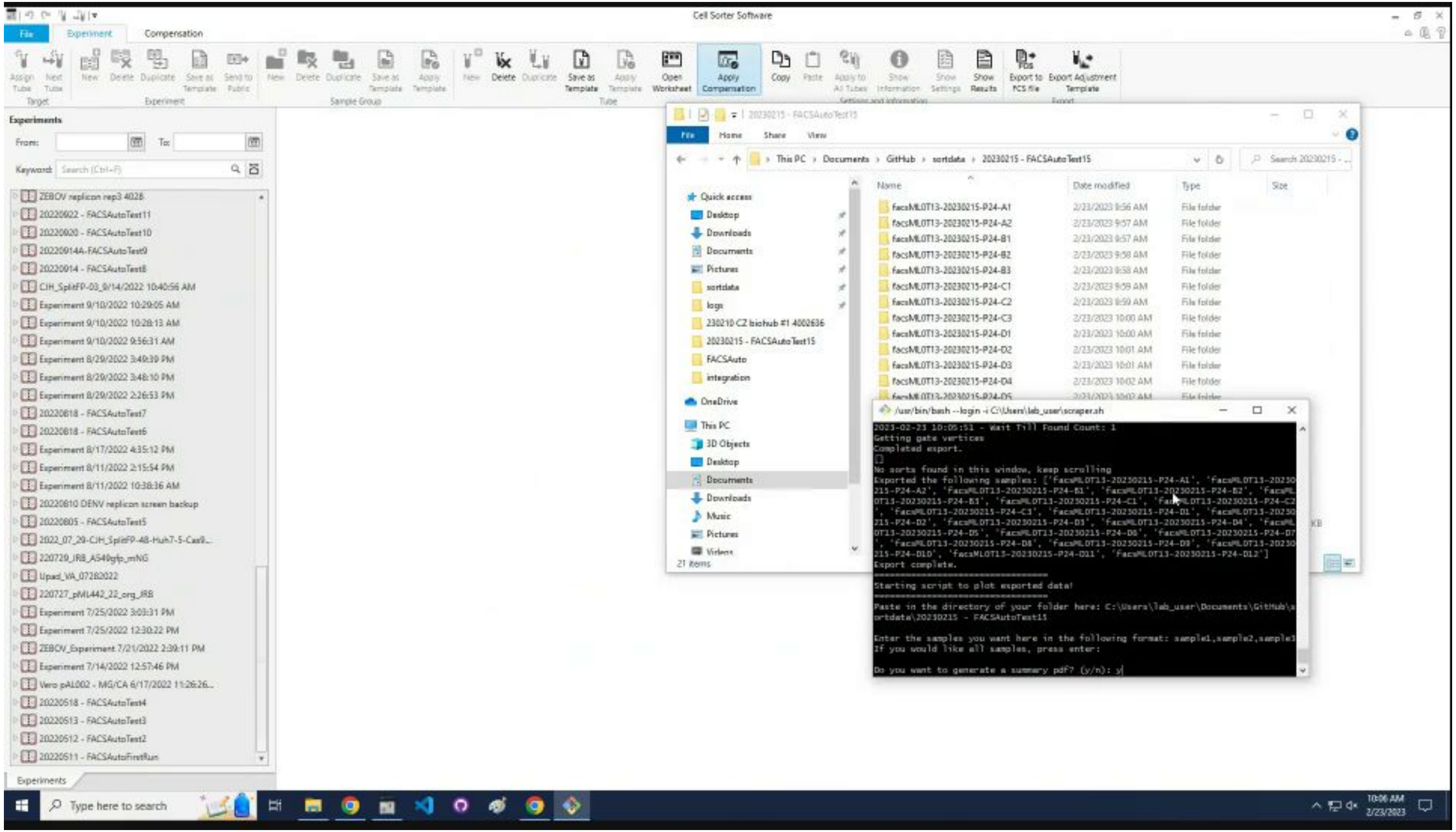

The summary PDF is in the folder; the program exits.

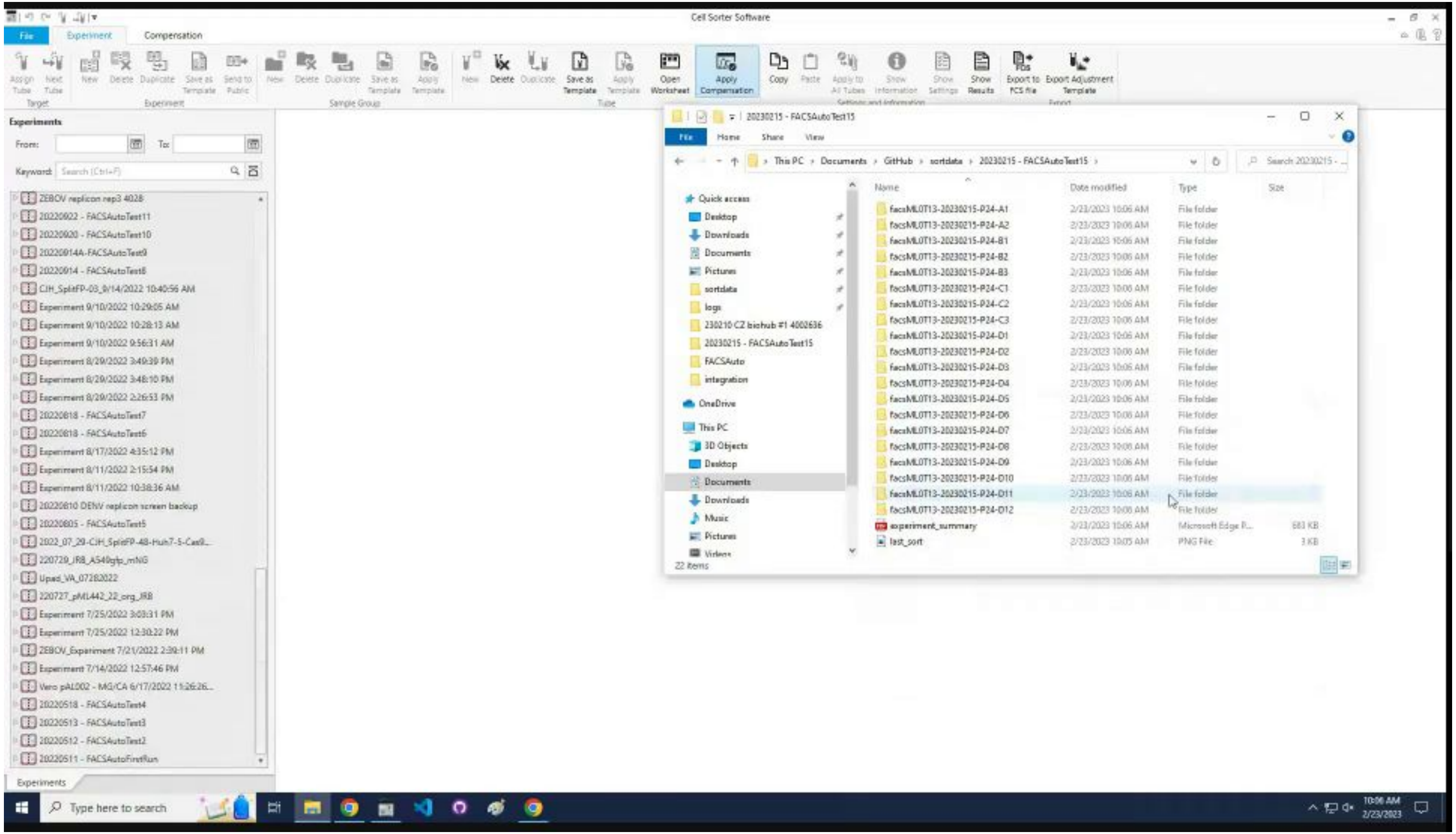

Supplement: S6 File — (PDF) [file pone.0299402.s010.pdf]
